# Cu(II) Coordination Polymers Containing Mixed Ligands with Different Flexibilities: Structural Diversity and Iodine Adsorption

**DOI:** 10.3390/molecules29020311

**Published:** 2024-01-08

**Authors:** Shu-Yu Lin, Yi-Lin Shen, Wei-Hao Chen, Manivannan Govindaraj, Jhy-Der Chen

**Affiliations:** 1 Department of Chemistry, Chung Yuan Christian University, Chung Li, Taoyuan City 320, Taiwan; kikimy123kikimy123@yahoo.com.tw (S.-Y.L.); lin740240@gmail.com (Y.-L.S.); a88q1127@gmail.com (W.-H.C.); 2 Department of Chemistry, Periyar Maniammai Institute of Science & Technology (Deemed to be University), Vallam, Thanjavur 613 403, Tamil Nadu, India

**Keywords:** coordination polymer, crystal structure analysis, tricarboxylate, coordination mode

## Abstract

Reactions of *N,N*′-*bis*(3-methylpyridyl)oxalamide (**L^1^**), *N*,*N*’-*bis*(3-methylpyridyl)adipoamide (**L^2^**) and *N*,*N*’-*bis*(3-methylpyridyl)sebacoamide (**L^3^**) with tricarboxylic acids and Cu(II) salts afforded {[Cu(**L^1^**)(1,3,5-HBTC)]·H_2_O}_n_ (1,3,5-H_3_BTC = 1,3,5-benzenetricarboxylic acid), **1**, {[Cu_1.5_(**L^2^**)_1.5_(1,3,5-BTC)(H_2_O)_2_]·6.5H_2_O}_n_, **2**, [Cu(**L^2^**)_0.5_(1,3,5-HBTB)]_n_ (1,3,5-H_3_BTB = 1,3,5-tri(4-carboxyphenyl)benzene), **3**, [Cu_4_(**L^3^**)(OH)_2_(1,3,5-BTC)_2_]_n_, **4**, {[Cu_3_(**L^3^**)_2_(1,3,5-BTB)_2_]·2.5MeOH·2H_2_O}_n_, **5**, and {[Cu_3_(**L^3^**)_2_(1,3,5-BTB)_2_ ]·DMF·2H_2_O}_n_, **6**, which have been structurally characterized by using single crystal X-ray crystallography. Complexes **1–4** form a 2D layer with the {4^4^.6^2^}-**sql** topology, a 2D layer with the (4.6^2^)_2_(4^2^.6^2^.8^2^)-**bex** topology, a three-fold interpenetrated 3D net with the (4^12^·6^3^)-**pcu** topology and a 3D framework with the (4^10^·6^32^·8^3^)(4^2^·6)_2_(4^3^·6^3^) topology, respectively, whereas **5** and **6** are 3D frameworks with the (6^3^)_2_(6^4^·8^2^)(6^8^·8^5^·10^2^) topology. Complex **5** shows a better iodine adsorption factor of 290.0 mg g^−1^ at 60 °C for 360 min than the other ones, revealing that the flexibility of the spacer ligand governs the structural diversity and the adsorption capacity.

## 1. Introduction

Coordination polymers (CPs) have shown crucial applications in many different areas due to their diverse structures and variable functions [1,2,3,4,5,6,7,8]. CPs can be constructed by the coordination of the designable spacer ligands to the metal ions, and through the self-assembly process, one- (1D), two- (2D) or three-dimensional (3D) network structures can be prepared. 

The –(CH_2_)_n_– group of the bis-pyridyl-bis-amide (bpba) possesses suitable flexibility that may adopt the coordination environment of different metal ions, whereas the two amide groups play important roles as abundant potential hydrogen bond sites, affording CPs with remarkable topologies. On the other hand, polycarboxylate ligands that show distinct coordination modes involving chelating and bridging are also important in the organization of CPs in a mixed system [9]. Benzene-1,3,5-tricarboxylic acid (1,3,5-H_3_BTC) is a planar molecule with *C_3_*-symmetry that may give anions of the types, BTC^3−^ and HBTC^2−^, and intriguing structural types have been found in the bpba-based CPs supported by these anions [10]. Extension of 1,3,5-H_3_BTC to the larger 1,3,5-tri(4-carboxyphenyl)benzene (1,3,5-H_3_BTB) may thus afford CPs with different structural topology. 

**Figure 1:**
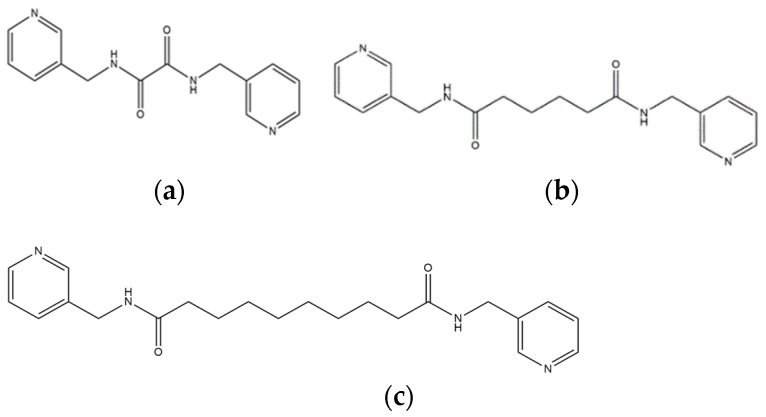
Structures of (**a**) **L^1^**, (**b**) **L^2^** and (**c**) **L^3^**.

**Figure 2:**
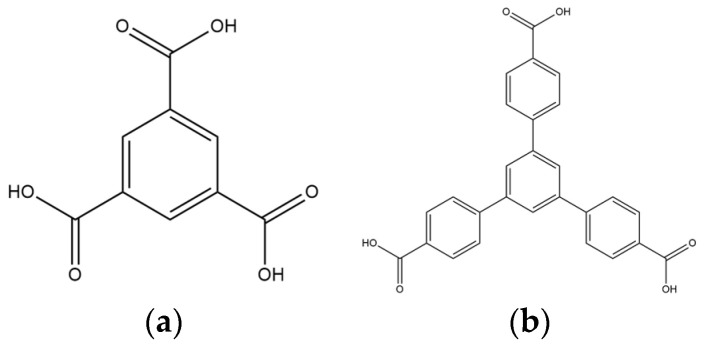
Structures of (**a**) 1,3,5-H_3_BTC and (**b**) 1,3,5-H_3_BTB.

We are dedicated to illuminate the factors that may direct the structural diversity and govern the adsorption property of the flexible bpba-based CPs by exploring the variations in ligand conformation and coordination mode of the spacer ligands. In this study, flexible *N*,*N*′-*bis*(3-pyridylmethyl)oxalamide (**L**^1^), *N*,*N*′-*bis*(3-methylpyridyl)adipoamide (**L**^2^) and *N*,*N*′-*bis*(3-methylpyridyl)sebacoamide (**L**^3^), Figure 1, were reacted with the Cu(II) metal salts and the tricarboxylic acids, 1,3,5-H_3_BTC, and 1,3,5-H_3_BTB, Figure 2, to yield {[Cu(**L**^1^)(1,3,5-HBTC)]·H_2_O}_n_ (1,3,5-H_3_BTC = 1,3,5-benzenetricarboxylic acid), **1**, {[Cu_1.5_(**L**^2^)_1.5_(1,3,5-BTC)(H_2_O)_2_]·6.5H_2_O}_n_, **2**, [Cu(**L**^2^)_0.5_(1,3,5-HBTB)]_n_ (1,3,5-H_3_BTB = 1,3,5-tri(4-carboxyphenyl)benzene), **3**, [Cu_4_(**L**^3^)(OH)_2_(1,3,5-BTC)_2_]_n_, **4**, {[Cu_3_(**L**^3^)_2_(1,3,5-BTB)_2_]·2.5MeOH·2H_2_O}_n_, **5**, and {[Cu_3_(**L**^3^)_2_(1,3,5-BTB)_2_]·DMF·2H_2_O}_n_, **6**. The synthesis and structures of 1–6 as well as their iodine adsorptions form the subject of this report. 

## 2. Results and Discussion

### 2.1. Structure of ***1***

**Figure 3:**
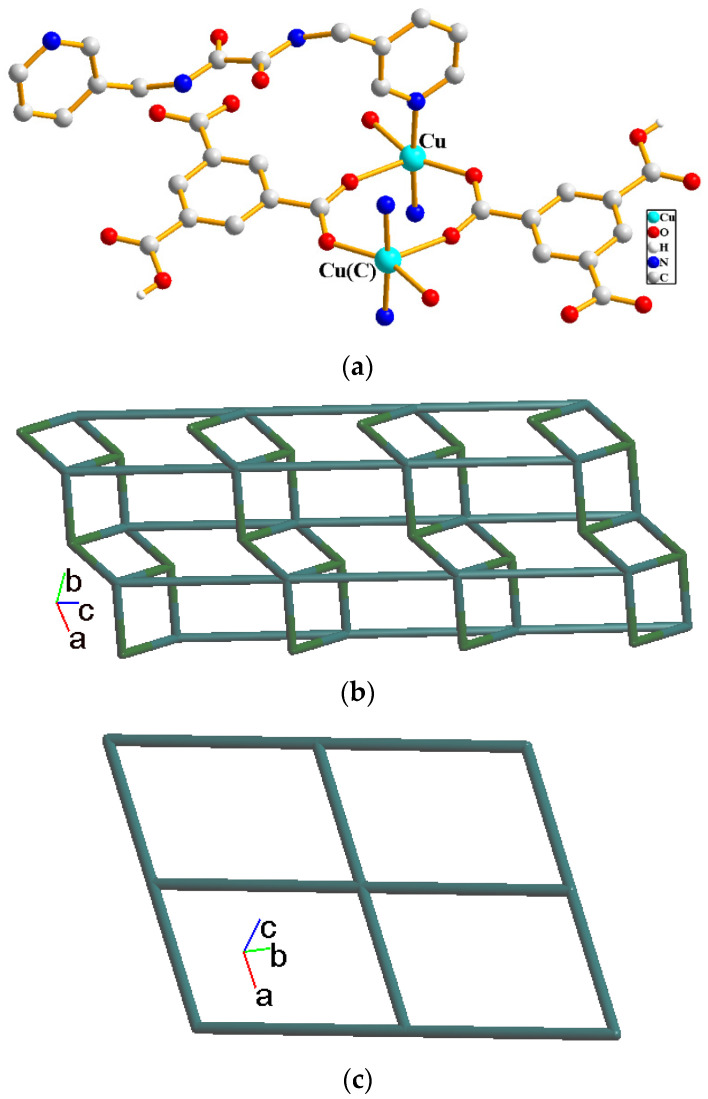
(**a**) Coordination environments of the Cu(II) cation in **1**. Symmetry transformations used to generate equivalent atoms: (C) −x + 1, −y + 2 and −z + 1. (**b**) A drawing showing the 2D net with (4^2^·6^7^·8)(4^2^·6)-3,5L2 topology. (**c**) A drawing showing the 2D net with (4^4^·6^2^)-**sql** topology.

A single-crystal X-ray diffraction analysis shows that complex **1** crystallizes in the triclinic space group *P*ī. There is one Cu(II) cation, one **L^1^** ligand, one 1,3,5-HBTC^2−^ ligand and one co-crystallized water molecule in the asymmetric unit. The Cu(II) cation is coordinated by two nitrogen atoms from **L^1^** ligands [Cu-N = 2.001(3)–2.007(3) Å] and three oxygen atoms from three 1,3,5-HBTC^2−^ ligands [Cu-O = 1.966(3)–2.237(3) Å], resulting in a trigonal bipyramidal geometry, and a dicopper unit is bridged by the 1,3,5-HBTC^2−^ ligands, Figure 3a. The Cu(II) cations are linked together by 1,3,5-HBTC^2−^ and **L^1^** ligands to afford a 2D structure. If the 1,3,5-HBTC^2−^ ligands are considered as three-connected nodes and the Cu(II) cations as five-connected nodes, the structure of **1** can be simplified as a 3,5-connected binodal 2D net with (4^2^·6^7^·8)(4^2^·6)-3,5L2 topology (standard representation), Figure 3b, determined by using ToposPro program [11]. If the dinuclear Cu(II) units are defined as four connected nodes, the structure can be simplified as a four-connected net with (4^4^·6^2^)-**sql** topology (cluster representation), Figure 3c.

### 2.2. Structure of ***2***

**Figure 4:**
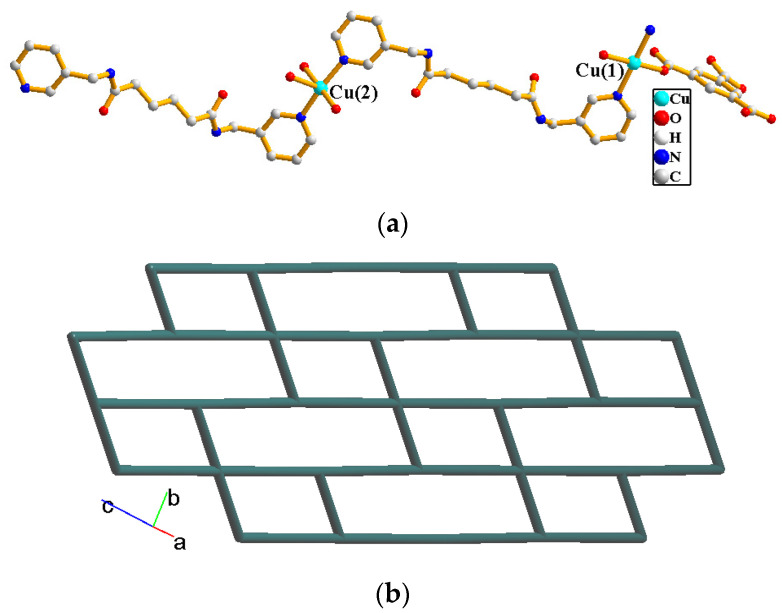
(**a**) Coordination environment of Cu(II) cations in **2**. (**b**) A drawing showing the 2D net with (4.6^2^)_2_(4^2^.6^2^.8^2^)-**bex** topology.

The crystals of complex **2** conform to the triclinic space group *P*ī and each asymmetric unit consists of two Cu(II) cations, one and a half **L^2^** ligands, one 1,3,5-BTC^3−^ ligand, two coordinated water molecules, and six and a half of a co-crystallized water molecules. The Cu(1) and Cu(2) metal centers are four- and five-coordinated, respectively, Figure 4a. While the Cu(1) atom is coordinated by two nitrogen atoms from the **L^2^** ligand [Cu-N = 2.051(2) Å] and two oxygen atoms from two 1,3,5-BTC^3−^ ligands [Cu-O = 1.960(2) Å], resulting in a distorted square geometry, the Cu(2) atom is coordinated by two nitrogen atoms from two **L^2^** ligands [Cu-N = 2.000(3)–2.006(3) Å], one oxygen atom from the 1,3,5-BTC^3−^ ligand [Cu-O = 1.978(2) Å] and two oxygen atoms from two coordinated water molecules [Cu-O = 2.023(2)–2.200(2) Å], giving a square pyramidal geometry. The Cu(II) cations are linked together by the 1,3,5-BTC^3−^ and **L^2^** ligands to afford a 2D layer. If the Cu(1) cations are defined as four-connected nodes and Cu(2) cations as three-connected nodes, the structure can be simplified as a 3,4-connected 2D net with (4.6^2^)_2_(4^2^.6^2^.8^2^)-**bex** topology, Figure 4b. 

### 2.3. Structure of ***3***

**Figure 5:**
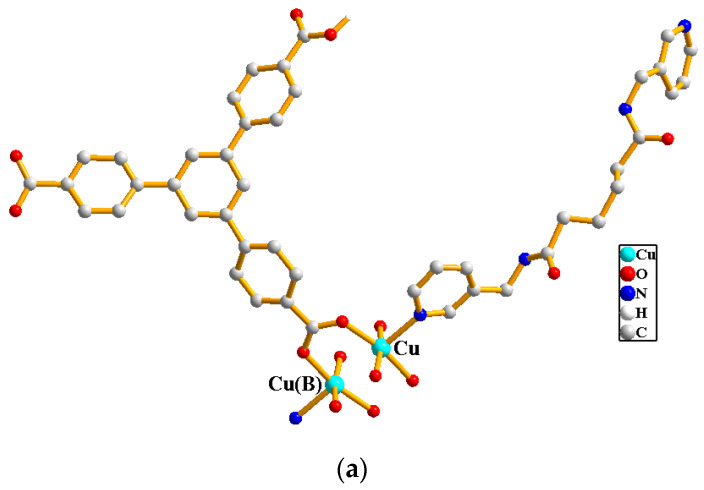

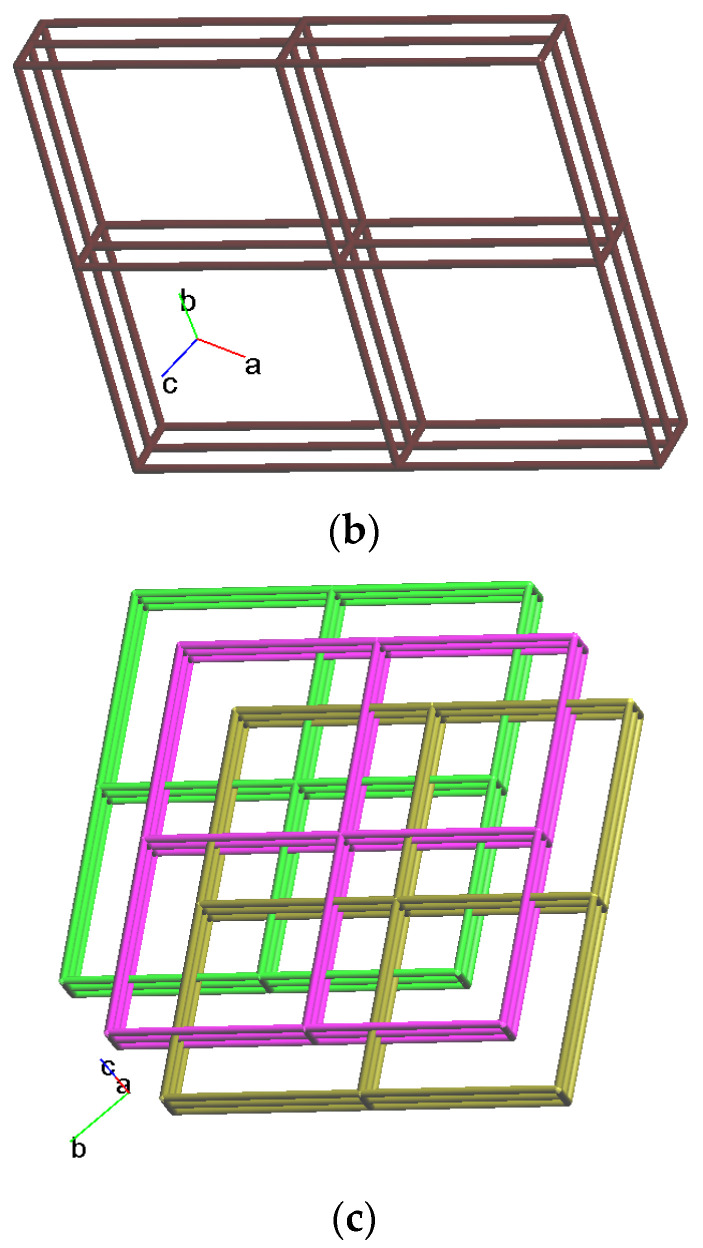
(**a**) Coordination environment of Cu(II) cations in **3**. Symmetry transformations used to generate equivalent atoms: (B) −x + 1, −y + 1 and −z + 2. (**b**) A drawing showing the structure with **pcu** topology. (**c**) A drawing showing the 3-fold interpenetrated net.

Complex **3** crystallizes in the monoclinic space group *C*2/*c*, and the asymmetric unit comprises one Cu(II) cation, a half of an **L^2^** ligand and one 1,3,5-HBTB^2−^ ligand. The Cu(II) cation is coordinated by one nitrogen atom from the **L^2^** ligand [Cu-N = 2.170(2) Å] and four oxygen atoms from four 1,3,5-HBTB^2−^ ligands [Cu-O = 1.9623(19)–1.9763(18) Å], resulting in a distorted square pyramidal geometry, Figure 5a. Two Cu(II) cations are bridged by the 1,3,5-HBTB^2−^ ligand to form a dinuclear unit with a Cu---Cu distance of 2.6516(6) Å that is shorter than the sum of two van der Waals radius of Cu (2.8 Å), suggesting the presence of weak intermolecular forces. The Cu(II) cations are linked together by 1,3,5-HBTB^2−^ and **L^2^** ligands to afford a 3D structure. If the dinuclear Cu(II) units are defined as six-connected nodes, the structure can be simplified as a six-connected net with (4^12^·6^3^)-**pcu** topology, Figure 5b. The 3D nets penetrate into the neighbors to form a threefold 3D interpenetration structure, Figure 5c, demonstrating that the combination of the flexible **L^2^** and 1,3,5-HBTB^2−^ may lead to the formation of the entangled CP [12].

### 2.4. Structure of ***4***

**Figure 6:**
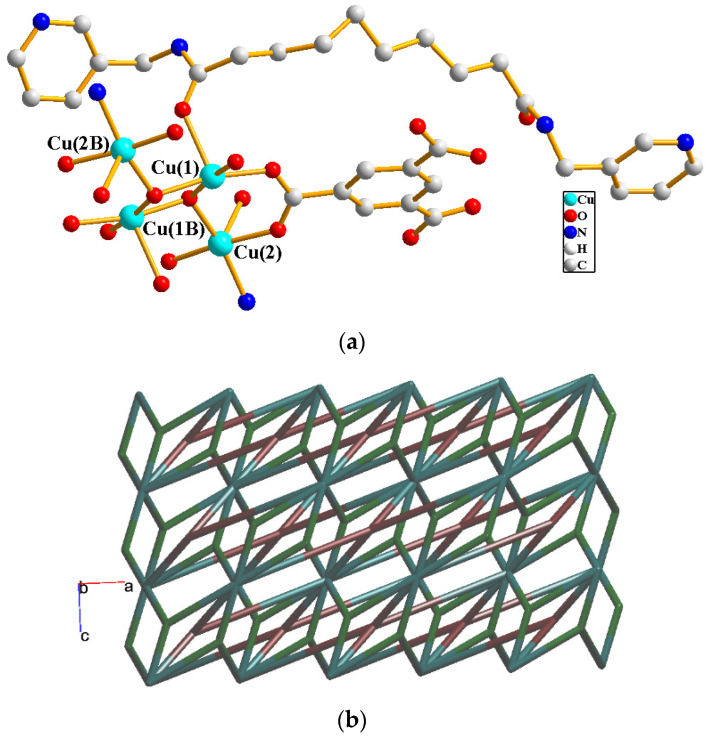
(**a**) Coordination environment of Cu(II) cations in **4**. Symmetry transformations used to generate equivalent atoms: (B) −x + 3/2, −y + 3/2 and −z + 1. (**b**) A drawing showing the 3D framework with (4^10^·6^32^·8^3^)(4^2^·6)_2_(4^3^·6^3^) topology.

The crystals of complex **4** conform to the monoclinic space group *C*2/*c*. The asymmetric unit consists of two Cu(II) cations, a half of an **L^3^** ligand, one 1,3,5-BTC^3−^ ligand and one hydroxide ion. Figure 6a depicts the coordination environments of the Cu(II) cations. The Cu(1) and Cu(1B) atoms are symmetrically -related by the inversion center, and each atom is coordinated by one oxygen atom from the **L^3^** ligand [Cu-O = 2.2720(16) Å], two oxygen atoms from two 1,3,5-BTC^3−^ ligands [Cu-O = 1.9377(14)–19434(14) Å] and two oxygen atoms from two hydroxy groups [Cu-O = 1.9551(13)–1.9620(13) Å], forming the square pyramidal geometry. On the other hand, each of the Cu(2) and Cu(2B) atoms is coordinated by one nitrogen atom from the **L^3^** ligand [Cu-N = 2.0164(17) Å], three oxygen atoms from three 1,3,5-BTC^3−^ ligands [Cu-O = 1.9473(13)–2.2909(15) Å] and one oxygen atom from the hydroxy group [Cu-O = 1.9482(13) Å], resulting in a distorted pentagonal bipyramidal geometry. The Cu(II) cations are linked together by 1,3,5-BTC^3−^ and **L^3^** ligands to afford a 3D structure. If the tetranuclear Cu(II) units are defined as ten-connected nodes, 1,3,5-BTC^3−^ ligands as three-connected nodes and **L^3^** as four-connected nodes, the structure can be simplified as a 3,4,10-connected trinodal net with the point symbol of (4^10^·6^32^·8^3^)(4^2^·6)_2_(4^3^·6^3^) topology, Figure 6b.

### 2.5. Structure of ***5*** and ***6***

**Figure 7:**
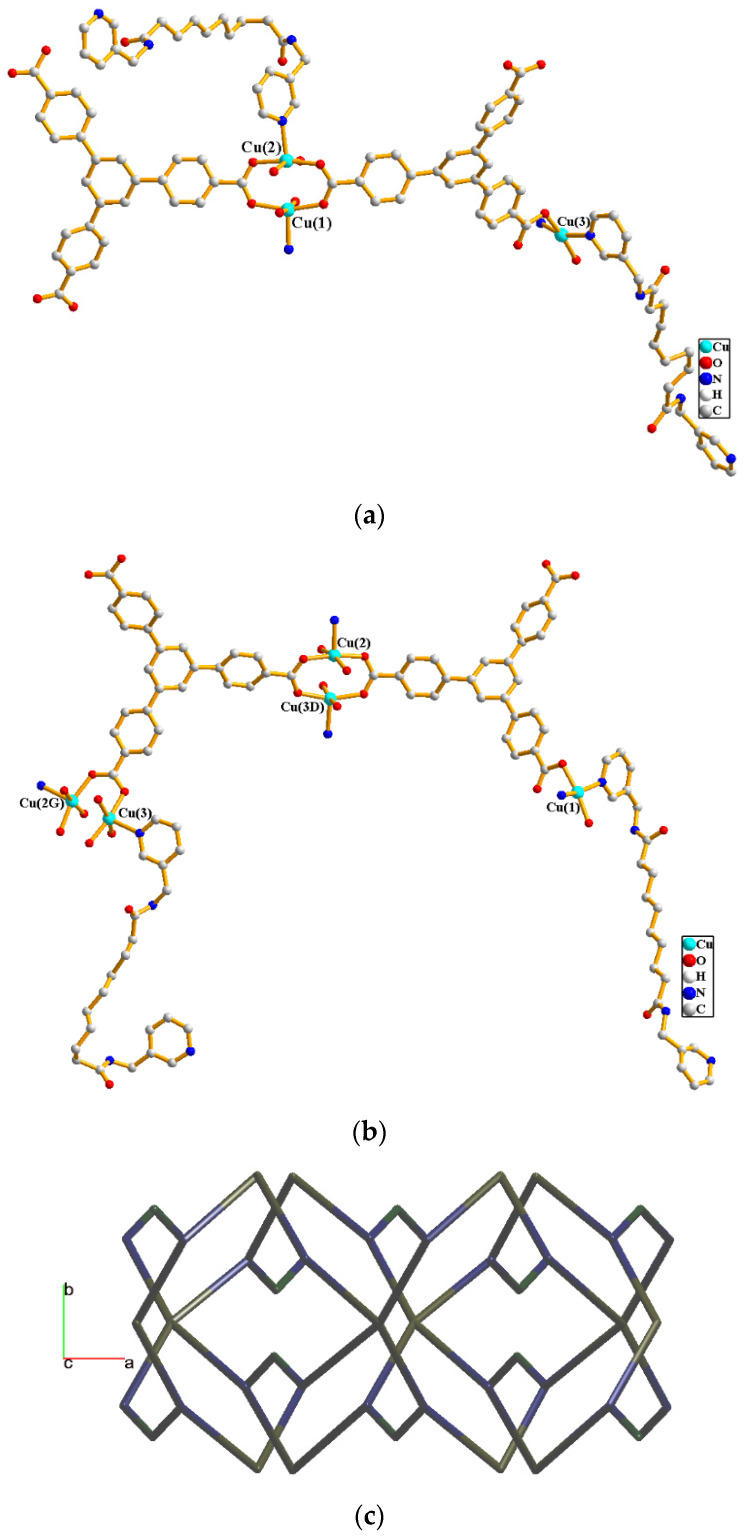
(**a**) Coordination environments of Cu(II) cations in **5**. (**b**) Coordination environments of Cu(II) cations in **6**. Symmetry transformations used to generate equivalent atoms: (D) x + 1/2, −y + 1/2 and z; (G) x − 1/2, −y + 1/2 and z. (**c**) A drawing showing the 3,4,6-connected net with the point symbol (6^3^)_2_(6^4^·8^2^)(6^8^·8^5^·10^2^).

Complexes **5** and **6** crystallize in the orthorhombic space group *P*na2**_1_**. Each of the asymmetric units of **5** and **6** comprise three Cu(II) cations, two **L^3^** ligands and two 1,3,5-BTB^3−^ ligands, with an additional two and a half co-crystallized methanol molecules and two co-crystallized water molecules in **5**, and two cocrystallized DMF molecules and two co-crystallized water molecules in **6**, respectively. Figure 7a shows the coordination environments of the Cu(II) cations in **5**. While the Cu(1) atom is coordinated by one nitrogen atom from the **L^3^** ligand [Cu(1)-N = 2.164(6) Å] and four oxygen atoms from four 1,3,5-BTB^3−^ ligands [Cu-O = 1.940(4)–2.005(4) Å], the Cu(2) atom is coordinated by one nitrogen atom from the **L^3^** ligand [Cu(2)-N = 2.189(6) Å] and four oxygen atoms from four 1,3,5-BTB^3−^ ligands [Cu-O = 1.933(5)–2.189(6) Å], resulting in square pyramidal geometries of both of the Cu(1) and Cu(2) atoms. The Cu(1) and Cu(2) atoms are bridged by the 1,3,5-BTB^3−^ ligands to form a dinuclear unit with a Cu---Cu distance of 2.6843(9) Å, indicating the presence of weak intermolecular forces. The Cu(3) atom is coordinated by two nitrogen atoms from two **L^3^** ligands [Cu-N = 2.054(7) and 2.079(7) Å] and two oxygen atoms from two 1,3,5-BTB^3−^ ligands [Cu-O = 1.913(6) and 1.917(5) Å], displaying a distorted square planar geometry.

Figure 7b shows the coordination environments of the Cu(II) cations in **6**. The Cu(1) atom is coordinated by two nitrogen atoms from the **L^3^** ligand [Cu-N = 2.027(6) and 2.034(6) Å] and two oxygen atoms from two 1,3,5-BTB^3−^ ligands [Cu-O = 1.918(5) and 1.941(5) Å], resulting in a distorted square planar geometry. Each of the Cu(2) and Cu(3) atoms is coordinated by one nitrogen atom from the **L^3^** ligand [Cu(2)-N = 2.183(5) Å; Cu(3)-N = 2.181(6) Å] and four oxygen atoms from four 1,3,5-BTB^3−^ ligands [Cu(2)-O = 1.929(4)–1.987(4) Å; Cu(3)-O = 1.929(4)–2.016(4) Å], resulting in square pyramidal geometries for Cu(2) and Cu(3). The Cu---Cu distance of 2.6922(9) Å between Cu(2) and Cu(3) is longer that in complex 5, indicating that the Cu(II)---Cu(II) interaction is subject to the nature of the co-crystallized solvent molecules. The Cu(II) cations in **5** and **6** are linked together by 1,3,5-BTB^3−^ and **L^3^** ligands to afford 3D structures. If the dinuclear Cu(II) units are defined as six-connected nodes, the mononuclear cations as four-connected nodes and 1,3,5-BTB^3−^ as three-connected nodes, while the **L^3^** ligands are defined as linkers, the structures of **5** and **6** can be simplified as 3,4,6-connected 3D nets with the point symbol of (6^3^)_2_(6^4^·8^2^)(6^8^·8^5^·10^2^), Figure 7c.

### 2.6. Ligand Conformations and Coordination Modes

The ligand conformations of the bpba ligands have been proposed based on the torsion angles (θ) of their methylene carbon atoms [0 ≤ θ ≤ 90°, gauche (G), and 90 < θ ≤ 180°, anti (A)]. On the other hand, *cis* and *trans* are given if the two C=O groups are in the same and opposite directions, respectively. Three orientations, *syn*-*syn*, *syn-anti* and *anti*-*anti*, are also defined based on the relative position of pyridyl nitrogen and amide oxygen atoms. Accordingly, the ligand conformations of **L^1^**–**L^3^** in **1**–**6** are listed in Table 1. It is also noted that while the bpba ligands in **1**, **2**, **3**, **5** and **6** bridge two Cu(II) cations through the two pyridyl nitrogen atoms, those in **4** bridge four Cu(II) cations through two pyridyl nitrogen and two amide oxygen atoms. Noticeably, although complexes **5** and **6** adopt the same structural type, the ligand conformations of the **L^3^** ligands are significantly different, presumably due to the difference in the co-crystallized solvents. Moreover, the tricarboxylate ligands in **1**–**6** bridge two to five Cu(II) cations through various coordination modes, which are also listed in Table 1. 

### 2.7. Powder X-ray Analysis

In order to check the phase purity of the products, powder X-ray diffraction (PXRD) experiments were carried out for all complexes. As shown in Figures S7–S12, the peak positions of the experimental and simulated PXRD patterns are in agreement with each other, which demonstrates that the crystal structures are truly representative of the bulk materials. The differences in intensity may be owing to the preferred orientation of the powder samples.

### 2.8. Thermal Properties

Thermal gravimetric analysis (TGA) was carried out to examine the thermal decomposition from 30 to 800 or 900 °C. The samples were heated in nitrogen gas at a pressure of 1 atm with heating rate of 10 °C min^−1^, and heating finished at 800 °C or 900 °C, Table 2, Figures S13–S18 indicate that complexes **1**–**6** display two-step weight loss involving a loss of solvent and a loss of organic ligands on heating.

### 2.9. Iodine Adsorption 

Radioactive iodine such as ^129^I represents one of the most critical nuclear wastes which is harmful to human health and has to be captured and disposed of effectively [13,14,15]. On the other hand, CPs possessing porous structures may facilitate iodine adsorption through noncovalent interactions involving iodine and various sorption sites. Iodine adsorption experiments were thus carried out for complexes **1**–**6** to evaluate the degree of adsorption of iodine vapor at 25 and 60 °C within time intervals of 30, 60, 120, 180 and 360 min, respectively. For each experiment, 10 mg of the complex was placed in a 4 mL sample bottle inside a 20 mL sample bottle containing 100 mg of iodine, which was then sealed and kept in the oven. Each experiment was repeated three times and the results averaged. It can be found that the colors of the complexes are different at different temperatures and time intervals, Figures S19–S30. 

Tables S1–S12 summarize the I_2_ adsorption of **1**–**6**, followed by the plots displaying iodine vapor adsorption rates. With the increase in temperature from 25 to 60 °C, the absorption rate of iodine also has a good increase for each complex, giving the best adsorption factor of 290.0 mg g^−1^ at 60 °C for 360 min for **5**. In order to confirm whether the structures of the iodine-adsorbed complexes remain unchanged, their powder X-ray diffraction (PXRD) patterns were measured. As shown in Figures S31–S42, most of the experimental patterns are consistent with the theoretical ones, but **2** at 60 °C for 30 and 60 min, and **5** and **6** at 60 °C for 60 min display some changes.

The ability of the CPs to adapt iodine molecules to the voids of the network structures may govern the iodine adsorption capacity [16,17,18,19]. The solvent accessible volumes calculated by using the PLATON program [20] for **1**–**6** were 1.5, 17.3, 34.4, 2.9, 11.8 and 10.4%, respectively, of the unit cell volume, indicating that complex **3,** which displays the three-fold interpenetrated 3D net with (4^12^·6^3^)-**pcu** topology, may accommodate more iodine than the other complexes. However, the best adsorption factor of 290.0 mg g^−1^ at 60 °C for 360 min was observed for **5**, demonstrating the important role of the flexibility of the neutral spacer ligands, **L^1^**, **L^2^** and **L^3^**, in determining the iodine adsorption capacities of **1**–**6**. The 3D framework of **5** containing the flexible **L^3^** may be more susceptible to the changes in the ligand conformation upon the attack of the iodine molecules and thus may be more appropriate to accommodate the iodine molecules, which can probably be verified by the subtle change in the PXRD pattern of **5**, Figure S40, upon iodine adsorption at 60 °C. On the other hand, the framework of the entangled **3** comprising **L^2^** is not vulnerable to the iodine attack, thus allowing for less iodine adsorption. The different performances in iodine adsorption between **5** and **6** are presumably due to the different co-crystallized solvents. The cavities of these complexes are small and thus most of the adsorptions are, as the reviewer suggested, due to surface uptakes.

We were not able to obtain the crystals suitable for single-crystal X-ray crystallography for the iodine-adsorbed samples. The iodine-adsorbed samples are usually anomalous, and energy dispersive X-ray (EDX) analysis was adopted to confirm iodine adsorption. The EDX experiments demonstrate the existence of the iodine atom rather than the identity of the iodine atom, *vide infra*. It has been reported that the iodine molecules can be transformed into anionic I_3_^−^ or I_5_^−^ in the iodine-adsorbed CPs [21]. Thus, it is not probable to determine whether the adsorption is reversible or irreversible at this moment.

Although the solvent accessible volumes (or the cavity sizes) of the CPs may govern such performances, the identities of the metal centers and the supporting ligands and stabilities of the CPs may also play important roles. The bpba ligands used in this report are well known for their amide groups that may interact with the incoming molecules through hydrogen bonds originating from the amine hydrogen atoms or the carbonyl oxygen atoms. By fixing the similar factors, we have shown that the best adsorption factor of 290.0 mg g^−1^ at 60 °C for 360 min was observed for **5**, demonstrating the important role of the flexibility of the neutral spacer ligands, **L^1^**, **L^2^** and **L^3^**, in determining the iodine adsorption capacities of **1**–**6**. For comparison, it is noted that the interpenetrated Th-SINAP-16 and Th-SINAP-21 appear to exhibit uptake amounts of 354 and 375 mg g^−1^, respectively, after 0.5 h of iodine adsorption [21], whereas the Ni(II) CP {[Ni(**L^3^**)(OBA)(H_2_O)_2_]·2H_2_O}_n_ encapsulates 166.55 mg g^−1^ iodine at 60 °C [16].

### 2.10. Energy Dispersive X-ray (EDX) Analysis

EDX analyses were performed for complexes **1**–**6** after iodine adsorption to investigate their iodine uptakes, Figures S43–S48. Three positions of the iodine-adsorbed samples of complexes **1**–**6** were selected for each measurement, and the amount of iodine was different for each position, indicating the inhomogeneous distribution of iodine in the iodine-adsorbed samples.

### 2.11. Gas Adsorption

Low-pressure N_2_ adsorption and desorption measurements were performed at 77 K for complexes **1**–**6**, which were heated at 120 °C for 24 h to obtain fully activated samples before the measurements. Figures S49–S54 demonstrate that the complexes **1** and **3**–**6** remain stable upon the removal of the solvent molecules, while the structure of **2** has changed. The Brunauer–Emmet–Teller (BET) surface areas are 7.49, 13.60, 12.82, 12.60, 11.73 and 7.98 m^2^/g, and the N_2_ uptake capacities are 6.93, 18.33, 12.70, 11.28, 10.83 and 8.12 cm^3^/g, respectively, for 1–6, Figures S55–S60, indicating larger surface area and N_2_ uptake for **2** upon desolvation. Moreover, pore-sized distribution curves show that the pore sizes are 2.9, 3.4, 3.4, 3.7, 3.4 and 3.4 nm, respectively, for **1**–**6**, Figures S61–S66.

As demonstrated by the experiments, the BET surface area and N_2_ uptakes of **1**–**6** derived from the low-pressure N_2_ adsorption and desorption measurements are not closely related to their iodine adsorption capacities. Therefore, the iodine adsorption capacity may also depend on the characteristics of the CPs and their surface features.

## 3. Materials and Methods

### 3.1. Materials

The reagent Cu(CH_3_COO)_2_·H_2_O was purchased from Showa, 1,3,5-benzenetricarboxylic acid (1,3,5-H_3_BTC) from Alfa Aesar and 1,3,5-tri(4-carboxyphenyl)benzene (1,3,5-H_3_BTB) from Alfa Aesar. The ligands *N,N*′-*bis*(3-methylpyridyl)oxalamide (**L^1^**), *N,N*′-*bis*(3-methylpyridyl)adipoamide (**L^2^**) and *N,N*′-*bis*(3-methylpyridyl)sebacoamide (**L^3^**) were prepared according to published procedures [22].

### 3.2. Preparations

#### 3.2.1. {[Cu(**L**^1^)(1,3,5-HBTC)]·H_2_O}_n_, **1**

A mixture of Cu(CH_3_COO)_2_·H_2_O (0.020 g, 0.10 mmol), **L^1^** (0.027 g, 0.10 mmol) and 1,3,5-H_3_BTC (0.021 g, 0.10 mmol) in 10 mL of H_2_O was sealed in a 23 mL Teflon-lined steel autoclave, which was heated under autogenous pressure to 120 °C for two days, and then cooled down to room temperature for two days. Blue crystals suitable for single-crystal X-ray diffraction were obtained. Yield: 0.081 g (72%). Anal. Calcd for C_23_H_20_CuN_4_O_9_ (MW = 599.97): C, 49.33; N, 10.00; H, 3.60%. Found: C, 49.39; N, 9.85; H, 3.48%. FT-IR (cm^−1^): 3313 (s), 2357 (m), 1621 (s), 1519 (m), 1430 (m), 1351 (s), 1099 (m), 860 (w), 756 (m), 609 (w) and 512 (w).

#### 3.2.2. {[Cu_1.5_(**L**^2^)_1.5_(1,3,5-BTC)(H_2_O)_2_]·6.5H_2_O}_n_, **2**

Complex **2** was prepared by following similar procedures for **1**, except that Cu(CH_3_COO)_2_·H_2_O (0.020 g, 0.10 mmol), **L^2^** (0.033 g, 0.10 mmol) and 1,3,5-H_3_BTC (0.021 g, 0.10 mmol) in 10 mL of NaOH (0.01 M) aqueous solution were used, which was heated to 100 °C. Blue crystals were obtained. Yield: 0.054 g (86%). Anal. Calcd for C_36_H_53_Cu_1.5_N_6_O_17.50_ (MW = 945.15): C, 45.75; N, 8.89; H, 5.65%. Found: C, 46.19; N, 8.69; H, 5.62%. FT-IR (cm^−1^): 3054 (m), 2359 (m), 1606 (s), 1427 (m), 1352 (s), 1196 (m), 1058 (w), 811 (w) and 710 (m).

#### 3.2.3. [Cu(**L**^2^)_0.5_(1,3,5-HBTB)]_n_, **3**

Complex **3** was prepared by following similar procedures for **1**, except that Cu(CH_3_COO)_2_·H_2_O (0.020 g, 0.10 mmol), **L^2^** (0.033 g, 0.10 mmol) and 1,3,5-H_3_BTB (0.044 g, 0.10 mmol) in 7 mL of H_2_O and 3 mL of DMA were used. Green crystals were obtained. Yield: 0.032 g (42%). Anal Calcd for C_36_H_27_CuN_2_O_7_ (MW = 663.13): C, 65.20; N, 4.22; H, 4.10%. Anal Calcd for C_36_H_27_CuN_2_O_7_·0.5DMA·3H_2_O (MW = 760.72): C, 59.99; N, 4.60; H, 4.97%. Found: C, 59.94; N, 4.84; H, 4.73%. FT-IR (cm^−1^): 2360 (m), 1604 (s), 1390 (s), 1176 (w), 1015 (m), 853 (w), 774 (s) and 669 (w).

#### 3.2.4. [Cu_4_(**L**^3^)(OH)_2_(1,3,5-BTC)_2_]_n_, **4**

Complex **4** was prepared by following similar procedures for **1**, except that Cu(CH_3_COO)_2_·H_2_O (0.020 g, 0.10 mmol), **L^3^** (0.038 g, 0.10 mmol) and 1,3,5-H_3_BTC (0.021 g, 0.10 mmol) in 10 mL of H_2_O were used. Blue crystals were obtained. Yield: 0.016 g (60%). Anal. Calcd for C_40_H_38_Cu_4_N_4_O_16_ (MW = 1084.90): C, 44.28; N, 5.16; H, 3.53%. Found: C, 44.49; N, 5.22; H, 3.87%. FT-IR (cm^−1^): 3238 (m), 2363 (m), 1583 (s), 1442 (m), 1353 (s), 1094 (w), 761 (m), 716 (m) and 586 (w).

#### 3.2.5. {[Cu_3_(**L**^3^)_2_(1,3,5-BTB)_2_]·2.5MeOH·2H_2_O}_n_, 5, and {[Cu_3_(**L**^3^)_2_(1,3,5-BTB)_2_]·DMF·2H_2_O}_n_, **6**

Complexes **5** and **6** were prepared by following similar procedures for **1** but in different solvent systems. While complex **5** was prepared from a reaction mixture of Cu(CH_3_COO)_2_·H_2_O (0.020 g, 0.10 mmol), **L^3^** (0.038 g, 0.10 mmol) and 1,3,5-H_3_BTB (0.044 g, 0.10 mmol) in 3 mL of H_2_O and 7 mL of MeOH, complex **6** was obtained in 7 mL of H_2_O and 3 mL of DMF. Green crystals were obtained for **5**. Yield: 0.018 g (28%). Anal. Calcd for C_100.50_H_104_Cu_3_N_8_O_20.50_ (MW = 1942.53): C, 62.14; N, 5.77; H, 5.40%. Found: C, 61.93; N, 5.59; H, 4.86%. FT-IR (cm^−1^): 3068 (m), 2925 (m), 2360 (w), 1596 (s), 1406 (s), 1179 (w), 1015 (w), 854 (w), 778 (m) and 700 (w). Green crystals were obtained for **6**. Yield: 0.036 g (56%). Anal. Calcd for C_101_H_101_Cu_3_N_9_O_19_ (MW = 1935.52): C, 62.67; N, 6.51; H, 5.26%. Anal. Calcd for **6** + 3 H_2_O, C_101_H_107_Cu_3_N_9_O_22_ (MW = 1989.52): C, 60.97; N, 6.34; H, 5.42%. Found: C, 60.67; N, 6.33; H, 5.02%. FT-IR (cm^−1^): 3065 (m), 2357 (w), 1599 (s), 1393 (s), 1177 (w), 1015 (m), 851 (m), 775 (s), 702 (m) and 668 (w).

The IR spectra of complexes **1**–**6** are provided as Figures S1–S6 in the Supplementary Materials.

### 3.3. X-ray Crystallography

A Bruker AXS SMART APEX II CCD diffractometer, equipped with a graphite-monochromated MoKα radiation (0.71073 Å), was used to collect diffraction data for complexes **1**–**6**. The diffraction data were then reduced by using standard methods [23], followed by empirical absorption corrections based on a “multi-scan”. The positions of some of the heavier atoms were located by the direct method or Patterson method, and the remaining atoms were found in a series of alternating difference Fourier maps and least-square refinements. The hydrogen atoms, except those of the water molecules, were added by using the HADD command in SHELXTL 6.1012 [24]. Due to the serious disordering, the solvent molecules in **3** were squeezed by using the PLATON program [20] and their diffraction data were reported without solvent contribution. Table 3 lists the crystal and structure refinement parameters for **1**–**6**. The CCDC no. 2311169-2311174 contains the supplementary crystallographic data for this paper. These data can be obtained free of charge via http://www.ccdc.cam.ac.uk/conts/retrieving.html or from the Cambridge Crystallographic Data Centre, 12 Union Road, Cambridge CB2 1EZ, UK; fax: +44 1223 336 033; e-mail: deposit@ccdc.cam.ac.uk; or at http://www.ccdc.cam.ac.uk.

## 4. Conclusions

Six new CPs supported by the mixed ligands with different flexibilities have been synthesized. Complexes **1**–**4** form a 2D layer with {4^4^.6^2^}-sql topology, a 2D layer with (4.6^2^)_2_(4^2^.6^2^.8^2^)-**bex** topology, a three-fold interpenetrated 3D net with (4^12^·6^3^)-**pcu** topology and a 3D net with (4^10^·6^32^·8^3^)(4^2^·6)_2_(4^3^·6^3^) topology, respectively, whereas **5** and **6** are 3D nets with the same (6^3^)_2_(6^4^·8^2^)(6^8^·8^5^·10^2^) topology, showing that the use of the extended 1,3,5-H_3_BTB afforded different structural types as compared with those derived from 1,3,5-H_3_BTC, and a combination of the flexible **L^2^** with 1,3,5-H_3_BTB gave an entangled CP. Among the six CPs, complex **5** reveals the best iodine adsorption capacity. This report offers an insight into understanding the roles of flexibility of the bpba and tricarboxylate ligands in determining the structural diversity as well as the iodine adsorption capacity.

## Figures and Tables

**Table 1:** Ligand conformations and bonding modes of complexes **1**–**6**.

	Ligand Conformation	Coordination Mode
**1**	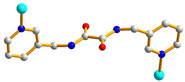 *trans syn*-*syn*	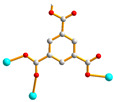 *μ*_3_-κO: κO′: κO″
**2**	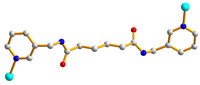 AAA *trans syn*-*syn*	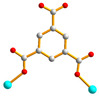 *μ*_2_-κO:κO′
**3**	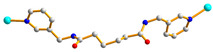 AGA *cis anti*-*anti*	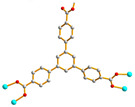 *μ*_4_-κO: κO′: κO″: κO‴
**4**	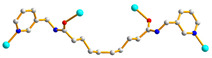 AAAAAAA *cis anti*-*anti*	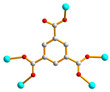 *μ*_5_-κO:κO′:O″:O‴:O″″
**5**	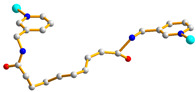 GGAAAAA *cis syn-syn*	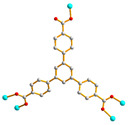 *μ*_5_-κO:κO′:O″:O‴:O″″
**6**	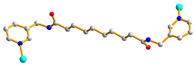 AAAAAAA *trans anti*-*anti*	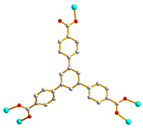 *μ*_5_-κO:κO′:O″:O‴:O″″

**Table 2:** Thermal properties of complexes **1**–**6**.

Complex	Weight Loss of Solvent °C (Calc/Found), %	Weight Loss of Ligand °C (Calc/Found), %
**1**	H_2_O 30–250 (3.21/4.38)	**L^1^** + 1,3,5-HBTC^2−^ 250–800 (85.14/85.15)
**2**	6.5 H_2_O 30–120 (16.19/14.38)	1.5 (**L^2^**) + 1,3,5-BTC^3−^ 120–900 (73.64/70.97)
**3**	0.5 DMA + 3 H_2_O 30–250 (12.83/7.86)	0.5 (**L^2^**) + 1,3,5-HBTB^2−^ 250–800 (78.75/81.97)
**4**	-	**L^3^** + 2 (1,3,5-BTC^3−^) + 2 (OH^−^) 270–800 (76.50/80.31)
**5**	2.5 MeOH + 2 H_2_O 30–270 (5.97/4.28)	2 (**L^3^**) + 2 (1,3,5-BTB^3−^) 270–800 (84.12/85.60)
**6**	DMF + H_2_O 30–250 (4.71/6.03)	2 (**L^3^**) + 2 (1,3,5-BTB^3−^) 250–800 (84.42/84.54)

**Table 3:** Crystal data for complexes **1**–**6**.

**Complex**	**1**	**2**	**3**
CCDC No.	2311169	2311170	2311171
Formula	C_23_H_20_CuN_4_O_9_	C_36_H_53_Cu_1.5_N_6_O_17.50_	C_36_H_27_CuN_2_O_7_
Formula weight	599.97	945.15	663.13
Crystal system	Triclinic	Triclinic	Monoclinic
Space group	*P*ī	*P*ī	*C*2/c
a, Å	10.1482(9)	8.9062(2)	18.8531(6)
b, Å	11.1855(10)	11.2565(3)	25.8032(8)
c, Å	11.7055(11)	22.7418(5)	17.4503(6)
α, °	111.285(3)	99.7718(14)	90
*β*, °	97.429(3)	94.3827(15)	99.4362(19)
γ,°	108.425(3)	105.8728(14)	90
V, Å^3^	1128.97(18)	2142.95(9)	8374.2(5)
Z	2	2	8
D_calc_, Mg/m^3^	1.647	1.465	1.052
F(000)	574	989	2736
µ(Mo K_α_), mm^−1^	1.032	0.831	0.561
Range(2θ) for data collection, deg	3.88 ≤ 2θ ≤ 51.99	3.66 ≤ 2θ ≤ 56.62	3.34 ≤ 2θ ≤ 56.62
Independent reflection	4415 [R(Int) = 0.0643]	10315 [R(Int) = 0.0539]	10067 [R(Int) = 0.0745]
Data/restraint/parameter	4415/0/338	10315/0/556	10067/0/432
quality-of-fit indicator ^c^	1.054	1.015	0.990
Final R indices [I > 2σ(I)] ^a,b^	R_1_ = 0.0555, wR_2_ = 0.1419	R_1_ = 0.0550, wR_2_ = 0.1228	R_1_ = 0.0536, wR_2_ = 0.1164
R indices (all data)	R_1_ = 0.0755, wR_2_ = 0.1668	R_1_ = 0.1158, wR_2_ = 0.1452	R_1_ = 0.1022, wR_2_ = 0.1345
**Complex**	**4**	**5**	**6**
CCDC No.	2311172	2311173	2311174
Formula	C_40_H_38_Cu_4_N_4_O_16_	C_100.50_H_104_Cu_3_N_8_O_20.50_	C_101_H_101_Cu_3_N_9_O_19_
Formula weight	1084.90	1942.53	1935.52
Crystal system	Monoclinic	Orthorhombic	Orthorhombic
Space group	*C*2/c	*P*na2_1_	*P*na2_1_
a, Å	16.5969(9)	20.9682(10)	22.0739(18)
b, Å	13.9067(4)	25.3489(11)	24.3392(18)
c, Å	17.6110(5)	18.1699(8)	17.9481(15)
α, °	90	90	90
*β*, °	90.2248(9)	90	90
γ,°	90	90	90
V, Å^3^	4064.73(19)	9657.7(8)	9642.8(13)
Z	4	4	4
D_calc_, Mg/m^3^	1.773	1.336	1.333
F(000)	2200	4056	4036
µ(Mo K_α_), mm^−1^	2.145	0.728	0.728
Range (2θ) for data collection, deg	3.82 ≤ 2θ ≤ 56.59	2.75 ≤ 2θ ≤ 51.99	2.82 ≤ 2θ ≤ 56.63
Independent reflection	5049 [R(Int) = 0.0283]	18994 [R(Int) = 0.0510]	20288 [R(Int) = 0.0765]
Data/restraint/parameter	5049/0/311	18994/2119/1157	20288/1/1181
quality-of-fit indicator ^c^	1.085	1.026	1.002
Final R indices [I > 2σ(I)] ^a,b^	R_1_ = 0.0265, wR_2_ = 0.0690	R_1_ = 0.0526, wR_2_ = 0.1408	R_1_ = 0.0571, wR_2_ = 0.1019
R indices (all data)	R_1_ = 0.0323, wR_2_ = 0.0747	R_1_ = 0.0664, wR_2_ = 0.1503	R_1_ = 0.1364, wR_2_ = 0.1244

^a^ R_1_ = ∑‖F_o_| − |F_c_‖/∑|F_o_|. ^b^ wR_2_ = [∑w(F_o_^2^ − F_c_^2^)^2^/∑w(F_o_^2^)^2^]^1/2^. w = 1/[σ^2^(F_o_^2^) + (ap)^2^ + (bp)]. *p* = [max(F_o_^2^ or 0) + 2(F_c_^2^)]/3. a = 0.1039, b = 1.1215 for 1; a = 0.0650, b = 0 for 2; a = 0.0585, b = 0 for 3; a = 0.0330, b = 9.2346 for 4; a = 0.0856, b = 10.2363 for 5; a = 0.0496, b = 0 for 6. ^c^ quality of fit = [∑w(|F_o_^2^| − |F_c_^2^|)^2^/N_observed_ − N_parameters_)]^1/2^.

## Data Availability

Data are contained within the article or Supplementary Materials.

## Supplementary Materials

The following supporting information can be downloaded at: https://www.mdpi.com/article/10.3390/molecules29020311/s1.
