# Supplementary material for: Cu(II) Coordination Polymers Containing Mixed Ligands with Different Flexibilities: Structural Diversity and Iodine Adsorption
Source: Molecules. 2024 Jan 8;29(2):311. doi: 10.3390/molecules29020311 (PMC10819037; doi:10.3390/molecules29020311)

checkCIF/PLATON report

Structure factors have been supplied for datablock(s) 1, 2, 3, 4, 5, 6

THIS REPORT IS FOR GUIDANCE ONLY. IF USED AS PART OF A REVIEW PROCEDURE FOR PUBLICATION, IT SHOULD NOT REPLACE THE EXPERTISE OF AN EXPERIENCED CRYSTALLOGRAPHIC REFEREE.

No syntax errors found. CIF dictionary Interpreting this report

Datablock: 1

```
Bond precision:   C-C = 0.0064 A          Wavelength=0.71073

Cell:          a=10.1482(9)       b=11.1855(10)     c=11.7055(11)
               alpha=111.285(3)   beta=97.429(3)    gamma=108.425(3)
Temperature:   296 K

                Calculated                  Reported
Volume          1128.98(18)                 1128.97(18)
Space group     P -1                        P -1
Hall group      -P 1                        -P 1
Moiety formula  C23 H18 Cu N4 O8, H2 O      ?
Sum formula     C23 H20 Cu N4 O9            C23 H20 Cu N4 O9
Mr              559.98                      559.97
Dx,g cm-3       1.647                       1.647
Z               2                           2
Mu (mm-1)       1.032                       1.032
F000            574.0                       574.0
F000'           574.92
h,k,lmax        12,13,14                    12,13,14
Nref            4437                        4415
Tmin,Tmax       0.899,0.961                 0.591,0.746
Tmin'           0.801

Correction method= # Reported T Limits: Tmin=0.591 Tmax=0.746
AbsCorr = MULTI_SCAN

Data completeness= 0.995          Theta(max)= 25.997

R(reflections)= 0.0555( 3489)                      wR2(reflections)=
                                                   0.1668( 4415)
S = 1.054                  Npar= 338
```

The following ALERTS were generated. Each ALERT has the format
test-name_ALERT_alert-type_alert-level.
Click on the hyperlinks for more details of the test.

Alert level C

```
PLAT341_ALERT_3_C Low Bond Precision on  C-C Bonds ...............    0.00645 Ang.
PLAT369_ALERT_2_C Long   C(sp2)-C(sp2) Bond  C7       - C8       .       1.55 Ang.
PLAT905_ALERT_3_C Negative K value in the Analysis of Variance ...     -1.362 Report
PLAT911_ALERT_3_C Missing FCF Refl Between Thmin & STh/L=    0.600         19 Report
```

Alert level G

```
PLAT004_ALERT_5_G Polymeric Structure Found with Maximum Dimension          2 Info
PLAT007_ALERT_5_G Number of Unrefined Donor-H Atoms .............           4 Report
PLAT154_ALERT_1_G The s.u.'s on the Cell Angles are Equal ..(Note)      0.003 Degree
PLAT232_ALERT_2_G Hirshfeld Test Diff (M-X)  Cu       --O4_e     .        5.1 s.u.
PLAT794_ALERT_5_G Tentative Bond Valency for Cu        (II)      .       2.20 Info
PLAT804_ALERT_5_G Number of ARU-Code Packing Problem(s) in PLATON          17 Info
PLAT883_ALERT_1_G No Info/Value for _atom_sites_solution_primary .     Please Do !
PLAT912_ALERT_4_G Missing # of FCF Reflections Above STh/L=  0.600          4 Note
PLAT967_ALERT_5_G Note: Two-Theta Cutoff Value in Embedded .res ..       52.0 Degree
PLAT978_ALERT_2_G Number C-C Bonds with Positive Residual Density.          4 Info
```

0 **ALERT level A** = Most likely a serious problem - resolve or explain
0 **ALERT level B** = A potentially serious problem, consider carefully
4 **ALERT level C** = Check. Ensure it is not caused by an omission or oversight
10 **ALERT level G** = General information/check it is not something unexpected

2 ALERT type 1 CIF construction/syntax error, inconsistent or missing data
3 ALERT type 2 Indicator that the structure model may be wrong or deficient
3 ALERT type 3 Indicator that the structure quality may be low
1 ALERT type 4 Improvement, methodology, query or suggestion
5 ALERT type 5 Informative message, check

Datablock: 2

Bond precision:	C-C = 0.0050 A		Wavelength=0.71073
Cell:	a=8.9062(2)	b=11.2565(3)	c=22.7418(5)
	alpha=99.7718(14)	beta=94.3827(15)	gamma=105.8728(14)
Temperature:	296 K		

	Calculated	Reported
Volume	2142.96(9)	2142.95(9)
Space group	P -1	P -1
Hall group	-P 1	-P 1
Moiety formula	C72 H88 Cu3 N12 O26, 10(H O0.50)	?
Sum formula	C72 H106 Cu3 N12 O35	C36 H53 Cu1.50 N6 O17.50
Mr	1890.34	945.15
Dx,g cm-3	1.465	1.465
Z	1	2
Mu (mm-1)	0.831	0.831
F000	989.0	989.0
F000'	990.46	
h,k,lmax	11,15,30	11,15,30
Nref	10661	10315
Tmin,Tmax	0.819,0.920	0.685,0.746
Tmin'	0.717	

Correction method= # Reported T Limits: Tmin=0.685 Tmax=0.746
AbsCorr = MULTI_SCAN

Data completeness= 0.968 Theta(max)= 28.310

R(reflections)= 0.0550(6028) wR2(reflections)= 0.1452(10315)

S = 1.015 Npar= 556

The following ALERTS were generated. Each ALERT has the format
test-name_ALERT_alert-type_alert-level.
Click on the hyperlinks for more details of the test.

Alert level C

```
PLAT220_ALERT_2_C NonSolvent   Resd 1  O   Ueq(max)/Ueq(min) Range        3.2 Ratio
PLAT260_ALERT_2_C Large Average Ueq of Residue Including      O18      0.181 Check
PLAT314_ALERT_2_C Small Angle for H2O: Metal-O12      -H12D      .      90.63 Degree
PLAT314_ALERT_2_C Small Angle for H2O: Metal-O15      -H15D      .      47.99 Degree
PLAT314_ALERT_2_C Small Angle for H2O: Metal-O15      -H15E      .      87.51 Degree
PLAT906_ALERT_3_C Large K Value in the Analysis of Variance ......      3.190 Check
PLAT911_ALERT_3_C Missing FCF Refl Between Thmin & STh/L=    0.600         66 Report
PLAT975_ALERT_2_C Check Calcd Resid. Dens.  0.41Ang From O14     .       0.52 eA-3
PLAT976_ALERT_2_C Check Calcd Resid. Dens.  0.57Ang From O13     .      -0.53 eA-3
PLAT976_ALERT_2_C Check Calcd Resid. Dens.  0.97Ang From O16     .      -0.46 eA-3
PLAT976_ALERT_2_C Check Calcd Resid. Dens.  1.07Ang From O16     .      -0.44 eA-3
PLAT976_ALERT_2_C Check Calcd Resid. Dens.  0.92Ang From O11     .      -0.42 eA-3
PLAT977_ALERT_2_C Check Negative Difference Density on H11D      .      -0.32 eA-3
PLAT977_ALERT_2_C Check Negative Difference Density on H16B      .      -0.42 eA-3
PLAT977_ALERT_2_C Check Negative Difference Density on H16C      .      -0.44 eA-3
```

Alert level G

```
PLAT004_ALERT_5_G Polymeric Structure Found with Maximum Dimension          2 Info
PLAT007_ALERT_5_G Number of Unrefined Donor-H Atoms .............          21 Report
PLAT045_ALERT_1_G Calculated and Reported Z Differ by a Factor ...      0.500 Check
PLAT300_ALERT_4_G Atom Site Occupancy of O18       Constrained at        0.5 Check
PLAT300_ALERT_4_G Atom Site Occupancy of H18D      Constrained at        0.5 Check
PLAT300_ALERT_4_G Atom Site Occupancy of H18E      Constrained at        0.5 Check
PLAT302_ALERT_4_G Anion/Solvent/Minor-Residue Disorder (Resd  6  )       100% Note
PLAT415_ALERT_2_G Short Inter D-H..H-X       H18D     ..H26A     .       2.07 Ang.
                                                1-x,2-y,2-z  =      2_677 Check
PLAT417_ALERT_2_G Short Inter D-H..H-D       H14B     ..H18E     .       2.11 Ang.
                                                      x,y,z  =      1_555 Check
PLAT794_ALERT_5_G Tentative Bond Valency for Cu1       (II)      .       2.04 Info
PLAT794_ALERT_5_G Tentative Bond Valency for Cu2       (II)      .       2.10 Info
PLAT804_ALERT_5_G Number of ARU-Code Packing Problem(s) in PLATON          9 Info
PLAT883_ALERT_1_G No Info/Value for _atom_sites_solution_primary .     Please Do !
PLAT899_ALERT_4_G SHELXL2018 is Deprecated and Succeeded by SHELXL     2019/3 Note
PLAT910_ALERT_3_G Missing # of FCF Reflection(s) Below Theta(Min).          1 Note
PLAT912_ALERT_4_G Missing # of FCF Reflections Above STh/L=  0.600        280 Note
PLAT933_ALERT_2_G Number of HKL-OMIT Records in Embedded .res File          2 Note
PLAT941_ALERT_3_G Average HKL Measurement Multiplicity ..........         3.1 Low
PLAT978_ALERT_2_G Number C-C Bonds with Positive Residual Density.          0 Info
```

---

0 **ALERT level A** = Most likely a serious problem - resolve or explain
0 **ALERT level B** = A potentially serious problem, consider carefully
15 **ALERT level C** = Check. Ensure it is not caused by an omission or oversight
19 **ALERT level G** = General information/check it is not something unexpected

2 ALERT type 1 CIF construction/syntax error, inconsistent or missing data
17 ALERT type 2 Indicator that the structure model may be wrong or deficient
4 ALERT type 3 Indicator that the structure quality may be low
6 ALERT type 4 Improvement, methodology, query or suggestion
5 ALERT type 5 Informative message, check

Datablock: 3

Bond precision: C-C = 0.0047 A Wavelength=0.71073

Cell:	a=18.8531(6)	b=25.8032(8)	c=17.4503(6)
	alpha=90	beta=99.4362(19)	gamma=90
Temperature:	191 K		

	Calculated	Reported
Volume	8374.2(5)	8374.2(5)
Space group	C 2/c	C 2/c
Hall group	-C 2yc	-C 2yc
Moiety formula	C36 H27 Cu N2 O7 [+ solvent]	?
Sum formula	C36 H27 Cu N2 O7 [+ solvent]	C36 H27 Cu N2 O7
Mr	663.15	663.13
Dx,g cm-3	1.052	1.052
Z	8	8
Mu (mm-1)	0.561	0.561
F000	2736.0	2736.0
F000'	2739.75	
h,k,lmax	25,34,23	25,34,23
Nref	10430	10067
Tmin,Tmax	0.935,0.945	0.674,0.746
Tmin'	0.845	

Correction method= # Reported T Limits: Tmin=0.674 Tmax=0.746
AbsCorr = MULTI_SCAN

Data completeness= 0.965 Theta(max)= 28.311

R(reflections)= 0.0536(6062) wR2(reflections)= 0.1345(10067)

S = 0.990 Npar= 432

The following ALERTS were generated. Each ALERT has the format
test-name_ALERT_alert-type_alert-level.
Click on the hyperlinks for more details of the test.

Alert level C

```
PLAT213_ALERT_2_C Atom C35              has ADP max/min Ratio .....        3.2 prolat
PLAT213_ALERT_2_C Atom C36              has ADP max/min Ratio .....        3.9 prolat
PLAT220_ALERT_2_C NonSolvent   Resd 1  C   Ueq(max)/Ueq(min) Range        4.6 Ratio
PLAT220_ALERT_2_C NonSolvent   Resd 1  O   Ueq(max)/Ueq(min) Range        4.1 Ratio
PLAT222_ALERT_3_C NonSolvent Resd 1  H   Uiso(max)/Uiso(min) Range        5.0 Ratio
PLAT241_ALERT_2_C High   'MainMol' Ueq as Compared to Neighbors of         N2 Check
PLAT242_ALERT_2_C Low    'MainMol' Ueq as Compared to Neighbors of         N1 Check
PLAT242_ALERT_2_C Low    'MainMol' Ueq as Compared to Neighbors of         C4 Check
PLAT242_ALERT_2_C Low    'MainMol' Ueq as Compared to Neighbors of        C27 Check
PLAT250_ALERT_2_C Large U3/U1 Ratio for Average U(i,j) Tensor ....        2.3 Note
PLAT360_ALERT_2_C Short  C(sp3)-C(sp3) Bond  C8       - C9       .       1.42 Ang.
PLAT420_ALERT_2_C D-H Bond Without Acceptor  N2       --H2A      .     Please Check
PLAT905_ALERT_3_C Negative K value in the Analysis of Variance ...     -2.133 Report
PLAT911_ALERT_3_C Missing FCF Refl Between Thmin & STh/L=    0.600        144 Report
```

Alert level G

```
PLAT007_ALERT_5_G Number of Unrefined Donor-H Atoms ..............          1 Report
PLAT171_ALERT_4_G The CIF-Embedded .res File Contains EADP Records          2 Report
PLAT301_ALERT_3_G Main Residue  Disorder ..............(Resd  1  )        20% Note
PLAT410_ALERT_2_G Short Intra H...H Contact  H18A     ..H31A     .       2.14 Ang.
                                                      x,y,z  =     1_555 Check
PLAT410_ALERT_2_G Short Intra H...H Contact  H18A     ..H31B     .       1.98 Ang.
                                                      x,y,z  =     1_555 Check
PLAT410_ALERT_2_G Short Intra H...H Contact  H20A     ..H36A     .       1.93 Ang.
                                                      x,y,z  =     1_555 Check
PLAT606_ALERT_4_G Solvent Accessible VOID(S) in Structure ........          ! Info
PLAT720_ALERT_4_G Number of Unusual/Non-Standard Labels ..........          3 Note
PLAT773_ALERT_2_G Check long C-C Bond in CIF: C2       --C3              1.90 Ang.
PLAT804_ALERT_5_G Number of ARU-Code Packing Problem(s) in PLATON           1 Info
PLAT869_ALERT_4_G ALERTS Related to the Use of SQUEEZE  Suppressed          ! Info
PLAT883_ALERT_1_G No Info/Value for _atom_sites_solution_primary .     Please Do !
PLAT899_ALERT_4_G SHELXL2018 is Deprecated and Succeeded by SHELXL     2019/3 Note
PLAT910_ALERT_3_G Missing # of FCF Reflection(s) Below Theta(Min).          2 Note
PLAT912_ALERT_4_G Missing # of FCF Reflections Above STh/L=  0.600        217 Note
PLAT913_ALERT_3_G Missing # of Very Strong Reflections in FCF ....          1 Note
PLAT941_ALERT_3_G Average HKL Measurement Multiplicity ...........        3.0 Low
PLAT978_ALERT_2_G Number C-C Bonds with Positive Residual Density.          3 Info
```

---

0 **ALERT level A** = Most likely a serious problem - resolve or explain
0 **ALERT level B** = A potentially serious problem, consider carefully
14 **ALERT level C** = Check. Ensure it is not caused by an omission or oversight
18 **ALERT level G** = General information/check it is not something unexpected

1 ALERT type 1 CIF construction/syntax error, inconsistent or missing data
16 ALERT type 2 Indicator that the structure model may be wrong or deficient
7 ALERT type 3 Indicator that the structure quality may be low
6 ALERT type 4 Improvement, methodology, query or suggestion
2 ALERT type 5 Informative message, check

Datablock: 4

Bond precision:	C-C = 0.0032 A		Wavelength=0.71073
Cell:	a=16.5969(4)	b=13.9067(4)	c=17.6110(5)
	alpha=90	beta=90.2248(9)	gamma=90
Temperature:	296 K		

	Calculated	Reported
Volume	4064.73(19)	4064.73(19)
Space group	C 2/c	C 2/c
Hall group	-C 2yc	-C 2yc
Moiety formula	C20 H19 Cu2 N2 O8	?
Sum formula	C20 H19 Cu2 N2 O8	C40 H38 Cu4 N4 O16
Mr	542.47	1084.90
Dx,g cm-3	1.773	1.773
Z	8	4
Mu (mm-1)	2.145	2.145
F000	2200.0	2200.0
F000'	2206.04	
h,k,lmax	22,18,23	22,18,23
Nref	5056	5049
Tmin,Tmax	0.652,0.778	0.689,0.740
Tmin'	0.639	

Correction method= # Reported T Limits: Tmin=0.689 Tmax=0.740
AbsCorr = MULTI_SCAN

Data completeness= 0.999 Theta(max)= 28.298

R(reflections)= 0.0265(4531) wR2(reflections)= 0.0747(5049)

S = 1.085 Npar= 311

The following ALERTS were generated. Each ALERT has the format
test-name_ALERT_alert-type_alert-level.
Click on the hyperlinks for more details of the test.

Alert level B

```
PLAT430_ALERT_2_B Short Inter D...A Contact  O4       ..N1       .       2.69 Ang.
                                              1+x,y,z  =      1_655 Check
```

Alert level C

```
PLAT329_ALERT_4_C Carbon Atom Hybridisation Unclear for ..........        C11 Check
PLAT329_ALERT_4_C Carbon Atom Hybridisation Unclear for ..........       C11' Check
PLAT911_ALERT_3_C Missing FCF Refl Between Thmin & STh/L=    0.600          8 Report
PLAT975_ALERT_2_C Check Calcd Resid. Dens.  1.01Ang From C11'    .       0.55 eA-3
```

Alert level G

```
PLAT004_ALERT_5_G Polymeric Structure Found with Maximum Dimension          3 Info
PLAT007_ALERT_5_G Number of Unrefined Donor-H Atoms ..............          1 Report
PLAT045_ALERT_1_G Calculated and Reported Z Differ by a Factor ...          2 Check
PLAT083_ALERT_2_G SHELXL Second Parameter in WGHT  Unusually Large       9.23 Why ?
```

```
PLAT300_ALERT_4_G Atom Site Occupancy of C10       Constrained at        0.5 Check
PLAT300_ALERT_4_G Atom Site Occupancy of C10'      Constrained at        0.5 Check
PLAT300_ALERT_4_G Atom Site Occupancy of C11       Constrained at        0.5 Check
PLAT300_ALERT_4_G Atom Site Occupancy of C11'      Constrained at        0.5 Check
PLAT300_ALERT_4_G Atom Site Occupancy of H10A      Constrained at        0.5 Check
PLAT300_ALERT_4_G Atom Site Occupancy of H10B      Constrained at        0.5 Check
PLAT300_ALERT_4_G Atom Site Occupancy of H10C      Constrained at        0.5 Check
PLAT300_ALERT_4_G Atom Site Occupancy of H10D      Constrained at        0.5 Check
PLAT300_ALERT_4_G Atom Site Occupancy of H11A      Constrained at        0.5 Check
PLAT300_ALERT_4_G Atom Site Occupancy of H11B      Constrained at        0.5 Check
PLAT300_ALERT_4_G Atom Site Occupancy of H11C      Constrained at        0.5 Check
PLAT300_ALERT_4_G Atom Site Occupancy of H11D      Constrained at        0.5 Check
PLAT301_ALERT_3_G Main Residue  Disorder ..............(Resd  1  )         6% Note
PLAT303_ALERT_2_G Full Occupancy Atom H8C       with # Connections       2.00 Check
PLAT367_ALERT_2_G Long?  C(sp?)-C(sp?) Bond  C8       - C9       .       1.52 Ang.
PLAT410_ALERT_2_G Short Intra H...H Contact  H9B      ..H10C     .       1.99 Ang.
                                                     x,y,z  =     1_555 Check
PLAT432_ALERT_2_G Short Inter X...Y Contact  O4       ..C1       .       2.86 Ang.
                                                   1+x,y,z  =     1_655 Check
PLAT764_ALERT_4_G Overcomplete CIF Bond List Detected (Rep/Expd) .       1.20 Ratio
PLAT794_ALERT_5_G Tentative Bond Valency for Cu1       (II)      .       2.13 Info
PLAT794_ALERT_5_G Tentative Bond Valency for Cu2       (II)      .       2.12 Info
PLAT883_ALERT_1_G No Info/Value for _atom_sites_solution_primary .     Please Do !
PLAT912_ALERT_4_G Missing # of FCF Reflections Above STh/L=  0.600          1 Note
PLAT913_ALERT_3_G Missing # of Very Strong Reflections in FCF ....          3 Note
PLAT965_ALERT_2_G The SHELXL WEIGHT Optimisation has not Converged     Please Check
PLAT978_ALERT_2_G Number C-C Bonds with Positive Residual Density.         10 Info
```

---

   0 **ALERT level A** = Most likely a serious problem - resolve or explain
   1 **ALERT level B** = A potentially serious problem, consider carefully
   4 **ALERT level C** = Check. Ensure it is not caused by an omission or oversight
  29 **ALERT level G** = General information/check it is not something unexpected

   2 ALERT type 1 CIF construction/syntax error, inconsistent or missing data
   9 ALERT type 2 Indicator that the structure model may be wrong or deficient
   3 ALERT type 3 Indicator that the structure quality may be low
  16 ALERT type 4 Improvement, methodology, query or suggestion
   4 ALERT type 5 Informative message, check

Datablock: 5

Bond precision:	C-C = 0.0114 A	Wavelength=0.71073	
Cell:	a=20.9682(10)	b=25.3489(11)	c=18.1699(8)
	alpha=90	beta=90	gamma=90
Temperature:	165 K		

	Calculated	Reported
Volume	9657.7(8)	9657.7(8)
Space group	P n a 21	P n a 21
Hall group	P 2c -2n	P 2c -2n
Moiety formula	2(C98 H84 Cu3 N8 O16), 6(C0.50 H2 O0.50), 4(H2 O)	?
Sum formula	C201 H196 Cu6 N16 O41	C100.50 H104 Cu3 N8 O20.50
Mr	3873.05	1942.53
Dx,g cm-3	1.332	1.336
Z	2	4
Mu (mm-1)	0.728	0.728
F000	4032.0	4056.0
F000'	4037.53	
h,k,lmax	25,31,22	25,31,22
Nref	19005[9820]	18994
Tmin,Tmax	0.840,0.930	0.672,0.746
Tmin'	0.695	

Correction method= # Reported T Limits: Tmin=0.672 Tmax=0.746
AbsCorr = MULTI_SCAN

Data completeness= 1.93/1.00 Theta(max)= 25.999

R(reflections)= 0.0526(15949) wR2(reflections)= 0.1503(18994)

S = 1.026 Npar= 1157

The following ALERTS were generated. Each ALERT has the format
test-name_ALERT_alert-type_alert-level.
Click on the hyperlinks for more details of the test.

Alert level C

```
PLAT041_ALERT_1_C Calc. and Reported SumFormula    Strings  Differ     Please Check
PLAT043_ALERT_1_C Calculated and Reported Mol. Weight Differ by ..      12.01 Check
PLAT068_ALERT_1_C Reported F000 Differs from Calcd (or Missing)...     Please Check
PLAT214_ALERT_2_C Atom C99       (Anion/Solvent) ADP max/min Ratio        4.1 prolat
PLAT220_ALERT_2_C NonSolvent   Resd 1  C   Ueq(max)/Ueq(min) Range        5.1 Ratio
PLAT220_ALERT_2_C NonSolvent   Resd 1  N   Ueq(max)/Ueq(min) Range        3.2 Ratio
PLAT220_ALERT_2_C NonSolvent   Resd 1  O   Ueq(max)/Ueq(min) Range        4.6 Ratio
PLAT222_ALERT_3_C NonSolvent Resd 1  H   Uiso(max)/Uiso(min) Range        4.2 Ratio
PLAT241_ALERT_2_C High   'MainMol' Ueq as Compared to Neighbors of         C8 Check
PLAT241_ALERT_2_C High   'MainMol' Ueq as Compared to Neighbors of        C39 Check
PLAT242_ALERT_2_C Low    'MainMol' Ueq as Compared to Neighbors of         N7 Check
PLAT242_ALERT_2_C Low    'MainMol' Ueq as Compared to Neighbors of         N8 Check
PLAT260_ALERT_2_C Large Average Ueq of Residue Including      O17      0.205 Check
PLAT260_ALERT_2_C Large Average Ueq of Residue Including      O18      0.121 Check
PLAT260_ALERT_2_C Large Average Ueq of Residue Including      O21      0.102 Check
PLAT309_ALERT_2_C Single Bonded Oxygen (C-O > 1.3 Ang) ..........         O2 Check
```

```
PLAT341_ALERT_3_C Low Bond Precision on  C-C Bonds ...............    0.01138 Ang.
PLAT911_ALERT_3_C Missing FCF Refl Between Thmin & STh/L=    0.600          5 Report
PLAT975_ALERT_2_C Check Calcd Resid. Dens.  0.81Ang From O19     .       0.88 eA-3
PLAT975_ALERT_2_C Check Calcd Resid. Dens.  1.03Ang From O4      .       0.43 eA-3
```

Alert level G

```
FORMU01_ALERT_2_G  There is a discrepancy between the atom counts in the
            _chemical_formula_sum and the formula from the _atom_site* data.
            Atom count from _chemical_formula_sum:C100.5 H104 Cu3 N8 O20.5
            Atom count from the _atom_site data:  C100.5 H98 Cu3 N8 O20.5
CELLZ01_ALERT_1_G Difference between formula and atom_site contents detected.
CELLZ01_ALERT_1_G WARNING: H atoms missing from atom site list. Is this intentional?
           From the CIF: _cell_formula_units_Z    4
           From the CIF: _chemical_formula_sum  C100.50 H104 Cu3 N8 O20.50
           TEST: Compare cell contents of formula and atom_site data

           atom    Z*formula  cif sites diff
           C        402.00    402.00    0.00
           H        416.00    392.00   24.00
           Cu        12.00     12.00    0.00
           N         32.00     32.00    0.00
           O         82.00     82.00    0.00
PLAT002_ALERT_2_G Number of Distance or Angle Restraints on AtSite         12 Note
PLAT003_ALERT_2_G Number of Uiso or Uij Restrained non-H Atoms ...        110 Report
PLAT004_ALERT_5_G Polymeric Structure Found with Maximum Dimension          3 Info
PLAT007_ALERT_5_G Number of Unrefined Donor-H Atoms .............          11 Report
PLAT045_ALERT_1_G Calculated and Reported Z Differ by a Factor ...      0.500 Check
PLAT083_ALERT_2_G SHELXL Second Parameter in WGHT  Unusually Large      10.24 Why ?
PLAT171_ALERT_4_G The CIF-Embedded .res File Contains EADP Records          2 Report
PLAT172_ALERT_4_G The CIF-Embedded .res File Contains DFIX Records          4 Report
PLAT178_ALERT_4_G The CIF-Embedded .res File Contains SIMU Records          1 Report
PLAT188_ALERT_3_G A Non-default SIMU Restraint Value has been used     0.0100 Report
PLAT300_ALERT_4_G Atom Site Occupancy of O2        Constrained at         0.5 Check
PLAT300_ALERT_4_G Atom Site Occupancy of O2'       Constrained at         0.5 Check
PLAT300_ALERT_4_G Atom Site Occupancy of C9        Constrained at         0.5 Check
PLAT300_ALERT_4_G Atom Site Occupancy of C9'       Constrained at         0.5 Check
PLAT300_ALERT_4_G Atom Site Occupancy of C10       Constrained at         0.5 Check
PLAT300_ALERT_4_G Atom Site Occupancy of C10'      Constrained at         0.5 Check
PLAT300_ALERT_4_G Atom Site Occupancy of C11       Constrained at         0.5 Check
PLAT300_ALERT_4_G Atom Site Occupancy of C11'      Constrained at         0.5 Check
PLAT300_ALERT_4_G Atom Site Occupancy of C12       Constrained at         0.5 Check
PLAT300_ALERT_4_G Atom Site Occupancy of C12'      Constrained at         0.5 Check
PLAT300_ALERT_4_G Atom Site Occupancy of C13       Constrained at         0.5 Check
PLAT300_ALERT_4_G Atom Site Occupancy of C13'      Constrained at         0.5 Check
PLAT300_ALERT_4_G Atom Site Occupancy of C14       Constrained at         0.5 Check
PLAT300_ALERT_4_G Atom Site Occupancy of C14'      Constrained at         0.5 Check
PLAT300_ALERT_4_G Atom Site Occupancy of C15       Constrained at         0.5 Check
PLAT300_ALERT_4_G Atom Site Occupancy of C15'      Constrained at         0.5 Check
PLAT300_ALERT_4_G Atom Site Occupancy of C16       Constrained at         0.5 Check
PLAT300_ALERT_4_G Atom Site Occupancy of C16'      Constrained at         0.5 Check
PLAT300_ALERT_4_G Atom Site Occupancy of C33       Constrained at         0.5 Check
PLAT300_ALERT_4_G Atom Site Occupancy of C33'      Constrained at         0.5 Check
PLAT300_ALERT_4_G Atom Site Occupancy of C34       Constrained at         0.5 Check
PLAT300_ALERT_4_G Atom Site Occupancy of C34'      Constrained at         0.5 Check
PLAT300_ALERT_4_G Atom Site Occupancy of C35       Constrained at         0.5 Check
PLAT300_ALERT_4_G Atom Site Occupancy of C35'      Constrained at         0.5 Check
```

```
PLAT300_ALERT_4_G Atom Site Occupancy of C36        Constrained at        0.5 Check
PLAT300_ALERT_4_G Atom Site Occupancy of C36’       Constrained at        0.5 Check
PLAT300_ALERT_4_G Atom Site Occupancy of H9’A       Constrained at        0.5 Check
PLAT300_ALERT_4_G Atom Site Occupancy of H9’B       Constrained at        0.5 Check
PLAT300_ALERT_4_G Atom Site Occupancy of H9A        Constrained at        0.5 Check
PLAT300_ALERT_4_G Atom Site Occupancy of H9B        Constrained at        0.5 Check
PLAT300_ALERT_4_G Atom Site Occupancy of H10A       Constrained at        0.5 Check
PLAT300_ALERT_4_G Atom Site Occupancy of H10B       Constrained at        0.5 Check
PLAT300_ALERT_4_G Atom Site Occupancy of H10C       Constrained at        0.5 Check
PLAT300_ALERT_4_G Atom Site Occupancy of H10D       Constrained at        0.5 Check
PLAT300_ALERT_4_G Atom Site Occupancy of H11A       Constrained at        0.5 Check
PLAT300_ALERT_4_G Atom Site Occupancy of H11B       Constrained at        0.5 Check
PLAT300_ALERT_4_G Atom Site Occupancy of H11C       Constrained at        0.5 Check
PLAT300_ALERT_4_G Atom Site Occupancy of H11D       Constrained at        0.5 Check
PLAT300_ALERT_4_G Atom Site Occupancy of H12A       Constrained at        0.5 Check
PLAT300_ALERT_4_G Atom Site Occupancy of H12B       Constrained at        0.5 Check
PLAT300_ALERT_4_G Atom Site Occupancy of H12C       Constrained at        0.5 Check
PLAT300_ALERT_4_G Atom Site Occupancy of H12D       Constrained at        0.5 Check
PLAT300_ALERT_4_G Atom Site Occupancy of H13A       Constrained at        0.5 Check
PLAT300_ALERT_4_G Atom Site Occupancy of H13B       Constrained at        0.5 Check
PLAT300_ALERT_4_G Atom Site Occupancy of H13C       Constrained at        0.5 Check
PLAT300_ALERT_4_G Atom Site Occupancy of H13D       Constrained at        0.5 Check
PLAT300_ALERT_4_G Atom Site Occupancy of H14A       Constrained at        0.5 Check
PLAT300_ALERT_4_G Atom Site Occupancy of H14B       Constrained at        0.5 Check
PLAT300_ALERT_4_G Atom Site Occupancy of H14C       Constrained at        0.5 Check
PLAT300_ALERT_4_G Atom Site Occupancy of H14D       Constrained at        0.5 Check
PLAT300_ALERT_4_G Atom Site Occupancy of H15A       Constrained at        0.5 Check
PLAT300_ALERT_4_G Atom Site Occupancy of H15B       Constrained at        0.5 Check
PLAT300_ALERT_4_G Atom Site Occupancy of H15C       Constrained at        0.5 Check
PLAT300_ALERT_4_G Atom Site Occupancy of H15D       Constrained at        0.5 Check
PLAT300_ALERT_4_G Atom Site Occupancy of H33A       Constrained at        0.5 Check
PLAT300_ALERT_4_G Atom Site Occupancy of H33B       Constrained at        0.5 Check
PLAT300_ALERT_4_G Atom Site Occupancy of H33C       Constrained at        0.5 Check
PLAT300_ALERT_4_G Atom Site Occupancy of H33D       Constrained at        0.5 Check
PLAT300_ALERT_4_G Atom Site Occupancy of H34A       Constrained at        0.5 Check
PLAT300_ALERT_4_G Atom Site Occupancy of H34B       Constrained at        0.5 Check
PLAT300_ALERT_4_G Atom Site Occupancy of H34C       Constrained at        0.5 Check
PLAT300_ALERT_4_G Atom Site Occupancy of H34D       Constrained at        0.5 Check
PLAT300_ALERT_4_G Atom Site Occupancy of H35A       Constrained at        0.5 Check
PLAT300_ALERT_4_G Atom Site Occupancy of H35B       Constrained at        0.5 Check
PLAT300_ALERT_4_G Atom Site Occupancy of H35C       Constrained at        0.5 Check
PLAT300_ALERT_4_G Atom Site Occupancy of H35D       Constrained at        0.5 Check
PLAT300_ALERT_4_G Atom Site Occupancy of H36A       Constrained at        0.5 Check
PLAT300_ALERT_4_G Atom Site Occupancy of H36B       Constrained at        0.5 Check
PLAT300_ALERT_4_G Atom Site Occupancy of H36C       Constrained at        0.5 Check
PLAT300_ALERT_4_G Atom Site Occupancy of H36D       Constrained at        0.5 Check
PLAT300_ALERT_4_G Atom Site Occupancy of O19        Constrained at        0.5 Check
PLAT300_ALERT_4_G Atom Site Occupancy of C101       Constrained at        0.5 Check
PLAT300_ALERT_4_G Atom Site Occupancy of H11E       Constrained at        0.5 Check
PLAT300_ALERT_4_G Atom Site Occupancy of H11F       Constrained at        0.5 Check
PLAT300_ALERT_4_G Atom Site Occupancy of H11G       Constrained at        0.5 Check
PLAT300_ALERT_4_G Atom Site Occupancy of H19O       Constrained at        0.5 Check
PLAT301_ALERT_3_G Main Residue  Disorder .............(Resd  1  )         10% Note
PLAT302_ALERT_4_G Anion/Solvent/Minor-Residue Disorder (Resd  4  )        100% Note
PLAT367_ALERT_2_G Long?  C(sp?)-C(sp?) Bond  C7       - C8       .        1.53 Ang.
PLAT367_ALERT_2_G Long?  C(sp?)-C(sp?) Bond  C31      - C32      .        1.50 Ang.
PLAT367_ALERT_2_G Long?  C(sp?)-C(sp?) Bond  C37      - C38      .        1.63 Ang.
```

```
PLAT413_ALERT_2_G Short Inter XH3 .. XHn     H10E     ..H14D     .       2.12 Ang.
                                      -1/2+x,-1/2-y,1+z  =      3_446 Check
PLAT414_ALERT_2_G Short Intra D-H..H-X       H7A      ..H36D     .       1.95 Ang.
                                                  x,y,z  =      1_555 Check
PLAT417_ALERT_2_G Short Inter D-H..H-D       H6A      ..H19O     .       2.08 Ang.
                                                  x,y,z  =      1_555 Check
PLAT417_ALERT_2_G Short Inter D-H..H-D       H19O     ..H21B     .       1.92 Ang.
                                            -x,-y,3/2+z  =      2_556 Check
PLAT417_ALERT_2_G Short Inter D-H..H-D       H19O     ..H21D     .       2.12 Ang.
                                            -x,-y,3/2+z  =      2_556 Check
PLAT720_ALERT_4_G Number of Unusual/Non-Standard Labels .........          2 Note
PLAT773_ALERT_2_G Check long C-C Bond in CIF: C34'     --C35'            1.71 Ang.
PLAT794_ALERT_5_G Tentative Bond Valency for Cu1       (II)      .       2.14 Info
PLAT794_ALERT_5_G Tentative Bond Valency for Cu2       (II)      .       2.20 Info
PLAT804_ALERT_5_G Number of ARU-Code Packing Problem(s) in PLATON         16 Info
PLAT860_ALERT_3_G Number of Least-Squares Restraints ............       2119 Note
PLAT883_ALERT_1_G No Info/Value for _atom_sites_solution_primary .     Please Do !
PLAT910_ALERT_3_G Missing # of FCF Reflection(s) Below Theta(Min).         1 Note
PLAT933_ALERT_2_G Number of HKL-OMIT Records in Embedded .res File         5 Note
PLAT965_ALERT_2_G The SHELXL WEIGHT Optimisation has not Converged     Please Check
PLAT967_ALERT_5_G Note: Two-Theta Cutoff Value in Embedded .res ..       52.0 Degree
PLAT978_ALERT_2_G Number C-C Bonds with Positive Residual Density.         1 Info
```

```
  0 ALERT level A = Most likely a serious problem - resolve or explain
  0 ALERT level B = A potentially serious problem, consider carefully
 20 ALERT level C = Check. Ensure it is not caused by an omission or oversight
111 ALERT level G = General information/check it is not something unexpected

  7 ALERT type 1 CIF construction/syntax error, inconsistent or missing data
 30 ALERT type 2 Indicator that the structure model may be wrong or deficient
  7 ALERT type 3 Indicator that the structure quality may be low
 81 ALERT type 4 Improvement, methodology, query or suggestion
  6 ALERT type 5 Informative message, check
```

Datablock: 6

Bond precision:	C-C = 0.0107 A		Wavelength=0.71073
Cell:	a=22.0739(18)	b=24.3392(18)	c=17.9481(15)
	alpha=90	beta=90	gamma=90
Temperature:	293 K		

	Calculated	Reported
Volume	9642.8(13)	9642.8(13)
Space group	P n a 21	P n a 21
Hall group	P 2c -2n	P 2c -2n
Moiety formula	C98 H88 Cu3 N8 O16, C3 H7 N O, 2(H2 O)	?
Sum formula	C101 H99 Cu3 N9 O19	C101 H101 Cu3 N9 O19
Mr	1933.55	1935.52
Dx,g cm-3	1.332	1.333
Z	4	4
Mu (mm-1)	0.728	0.728
F000	4028.0	4036.0
F000'	4033.48	
h,k,lmax	29,32,23	29,32,23
Nref	24020[12386]	20288
Tmin,Tmax	0.840,0.930	0.690,0.746
Tmin'	0.747	

Correction method= # Reported T Limits: Tmin=0.690 Tmax=0.746
AbsCorr = MULTI_SCAN

Data completeness= 1.64/0.84 Theta(max)= 28.316

R(reflections)= 0.0571(11504) wR2(reflections)= 0.1244(20288)

S = 1.002 Npar= 1181

The following ALERTS were generated. Each ALERT has the format
test-name_ALERT_alert-type_alert-level.
Click on the hyperlinks for more details of the test.

Alert level C

```
PLAT041_ALERT_1_C Calc. and Reported SumFormula    Strings  Differ     Please Check
PLAT068_ALERT_1_C Reported F000 Differs from Calcd (or Missing)...     Please Check
PLAT213_ALERT_2_C Atom N4              has ADP max/min Ratio .....        3.1 prolat
PLAT214_ALERT_2_C Atom C99       (Anion/Solvent) ADP max/min Ratio        4.4 prolat
PLAT220_ALERT_2_C NonSolvent   Resd 1  C   Ueq(max)/Ueq(min) Range        5.9 Ratio
PLAT220_ALERT_2_C NonSolvent   Resd 1  O   Ueq(max)/Ueq(min) Range        3.8 Ratio
PLAT222_ALERT_3_C NonSolvent Resd 1  H   Uiso(max)/Uiso(min) Range        5.0 Ratio
PLAT241_ALERT_2_C High   'MainMol' Ueq as Compared to Neighbors of        C11 Check
PLAT241_ALERT_2_C High   'MainMol' Ueq as Compared to Neighbors of        C15 Check
PLAT241_ALERT_2_C High   'MainMol' Ueq as Compared to Neighbors of        C59 Check
PLAT241_ALERT_2_C High   'MainMol' Ueq as Compared to Neighbors of        C93 Check
PLAT242_ALERT_2_C Low    'MainMol' Ueq as Compared to Neighbors of         C7 Check
PLAT242_ALERT_2_C Low    'MainMol' Ueq as Compared to Neighbors of        C12 Check
PLAT242_ALERT_2_C Low    'MainMol' Ueq as Compared to Neighbors of        C16 Check
PLAT242_ALERT_2_C Low    'MainMol' Ueq as Compared to Neighbors of        C56 Check
PLAT242_ALERT_2_C Low    'MainMol' Ueq as Compared to Neighbors of        C91 Check
```

```
PLAT244_ALERT_4_C Low    'Solvent' Ueq as Compared to Neighbors of         N9 Check
PLAT244_ALERT_4_C Low    'Solvent' Ueq as Compared to Neighbors of       C101 Check
PLAT245_ALERT_2_C U(iso) H97A      Smaller than U(eq) C98       by      0.038 Ang**2
PLAT245_ALERT_2_C U(iso) H97B      Smaller than U(eq) C98       by      0.038 Ang**2
PLAT260_ALERT_2_C Large Average Ueq of Residue Including       O19      0.242 Check
PLAT329_ALERT_4_C Carbon Atom Hybridisation Unclear for ..........        C60 Check
PLAT341_ALERT_3_C Low Bond Precision on  C-C Bonds ...............    0.01069 Ang.
```

Alert level G

```
FORMU01_ALERT_2_G  There is a discrepancy between the atom counts in the
            _chemical_formula_sum and the formula from the _atom_site* data.
            Atom count from _chemical_formula_sum:C101 H101 Cu3 N9 O19
            Atom count from the _atom_site data:  C101 H99 Cu3 N9 O19
CELLZ01_ALERT_1_G Difference between formula and atom_site contents detected.
CELLZ01_ALERT_1_G WARNING: H atoms missing from atom site list. Is this intentional?
           From the CIF: _cell_formula_units_Z    4
           From the CIF: _chemical_formula_sum  C101 H101 Cu3 N9 O19
           TEST: Compare cell contents of formula and atom_site data

           atom    Z*formula  cif sites diff
           C        404.00    404.00    0.00
           H        404.00    396.00    8.00
           Cu        12.00     12.00    0.00
           N         36.00     36.00    0.00
           O         76.00     76.00    0.00
PLAT004_ALERT_5_G Polymeric Structure Found with Maximum Dimension          3 Info
PLAT007_ALERT_5_G Number of Unrefined Donor-H Atoms ..............          8 Report
PLAT199_ALERT_1_G Reported _cell_measurement_temperature ..... (K)        293 Check
PLAT200_ALERT_1_G Reported   _diffrn_ambient_temperature ..... (K)        293 Check
PLAT300_ALERT_4_G Atom Site Occupancy of C19       Constrained at        0.5 Check
PLAT300_ALERT_4_G Atom Site Occupancy of C19'      Constrained at        0.5 Check
PLAT300_ALERT_4_G Atom Site Occupancy of C20       Constrained at        0.5 Check
PLAT300_ALERT_4_G Atom Site Occupancy of C20'      Constrained at        0.5 Check
PLAT300_ALERT_4_G Atom Site Occupancy of C21       Constrained at        0.5 Check
PLAT300_ALERT_4_G Atom Site Occupancy of C21'      Constrained at        0.5 Check
PLAT300_ALERT_4_G Atom Site Occupancy of C60       Constrained at        0.5 Check
PLAT300_ALERT_4_G Atom Site Occupancy of C60'      Constrained at        0.5 Check
PLAT300_ALERT_4_G Atom Site Occupancy of C95       Constrained at        0.5 Check
PLAT300_ALERT_4_G Atom Site Occupancy of C95'      Constrained at        0.5 Check
PLAT300_ALERT_4_G Atom Site Occupancy of C96       Constrained at        0.5 Check
PLAT300_ALERT_4_G Atom Site Occupancy of C96'      Constrained at        0.5 Check
PLAT300_ALERT_4_G Atom Site Occupancy of C97       Constrained at        0.5 Check
PLAT300_ALERT_4_G Atom Site Occupancy of C97'      Constrained at        0.5 Check
PLAT300_ALERT_4_G Atom Site Occupancy of C98       Constrained at        0.5 Check
PLAT300_ALERT_4_G Atom Site Occupancy of C98'      Constrained at        0.5 Check
PLAT300_ALERT_4_G Atom Site Occupancy of H19A      Constrained at        0.5 Check
PLAT300_ALERT_4_G Atom Site Occupancy of H19B      Constrained at        0.5 Check
PLAT300_ALERT_4_G Atom Site Occupancy of H20A      Constrained at        0.5 Check
PLAT300_ALERT_4_G Atom Site Occupancy of H20B      Constrained at        0.5 Check
PLAT300_ALERT_4_G Atom Site Occupancy of H21A      Constrained at        0.5 Check
PLAT300_ALERT_4_G Atom Site Occupancy of H21B      Constrained at        0.5 Check
PLAT300_ALERT_4_G Atom Site Occupancy of H60A      Constrained at        0.5 Check
PLAT300_ALERT_4_G Atom Site Occupancy of H60B      Constrained at        0.5 Check
PLAT300_ALERT_4_G Atom Site Occupancy of H60C      Constrained at        0.5 Check
PLAT300_ALERT_4_G Atom Site Occupancy of H60D      Constrained at        0.5 Check
PLAT300_ALERT_4_G Atom Site Occupancy of H95A      Constrained at        0.5 Check
```

```
PLAT300_ALERT_4_G Atom Site Occupancy of H95B       Constrained at       0.5 Check
PLAT300_ALERT_4_G Atom Site Occupancy of H95C       Constrained at       0.5 Check
PLAT300_ALERT_4_G Atom Site Occupancy of H95D       Constrained at       0.5 Check
PLAT300_ALERT_4_G Atom Site Occupancy of H96A       Constrained at       0.5 Check
PLAT300_ALERT_4_G Atom Site Occupancy of H96B       Constrained at       0.5 Check
PLAT300_ALERT_4_G Atom Site Occupancy of H96C       Constrained at       0.5 Check
PLAT300_ALERT_4_G Atom Site Occupancy of H96D       Constrained at       0.5 Check
PLAT300_ALERT_4_G Atom Site Occupancy of H97A       Constrained at       0.5 Check
PLAT300_ALERT_4_G Atom Site Occupancy of H97B       Constrained at       0.5 Check
PLAT300_ALERT_4_G Atom Site Occupancy of H97C       Constrained at       0.5 Check
PLAT300_ALERT_4_G Atom Site Occupancy of H97D       Constrained at       0.5 Check
PLAT300_ALERT_4_G Atom Site Occupancy of H98A       Constrained at       0.5 Check
PLAT300_ALERT_4_G Atom Site Occupancy of H98B       Constrained at       0.5 Check
PLAT300_ALERT_4_G Atom Site Occupancy of H98C       Constrained at       0.5 Check
PLAT300_ALERT_4_G Atom Site Occupancy of H98D       Constrained at       0.5 Check
PLAT301_ALERT_3_G Main Residue  Disorder .............(Resd  1  )         6% Note
PLAT367_ALERT_2_G Long?  C(sp?)-C(sp?) Bond  C58      - C59      .      1.52 Ang.
PLAT380_ALERT_4_G Incorrectly? Oriented X(sp2)-Methyl Moiety .....       C99 Check
PLAT380_ALERT_4_G Incorrectly? Oriented X(sp2)-Methyl Moiety .....      C100 Check
PLAT410_ALERT_2_G Short Intra H...H Contact  H17B     ..H19A     .      2.09 Ang.
                                                    x,y,z  =     1_555 Check
PLAT773_ALERT_2_G Check long C-C Bond in CIF: C59      --C60            1.90 Ang.
PLAT794_ALERT_5_G Tentative Bond Valency for Cu2       (II)      .      2.20 Info
PLAT794_ALERT_5_G Tentative Bond Valency for Cu3       (II)      .      2.16 Info
PLAT804_ALERT_5_G Number of ARU-Code Packing Problem(s) in PLATON         17 Info
PLAT850_ALERT_4_G Check Flack Parameter Exact Value 0.00 with s.u.      0.01 Check
PLAT883_ALERT_1_G No Info/Value for _atom_sites_solution_primary .    Please Do !
PLAT910_ALERT_3_G Missing # of FCF Reflection(s) Below Theta(Min).         1 Note
PLAT912_ALERT_4_G Missing # of FCF Reflections Above STh/L=  0.600        46 Note
PLAT915_ALERT_3_G No Flack x Check Done: Low Friedel Pair Coverage        68 %
PLAT978_ALERT_2_G Number C-C Bonds with Positive Residual Density.         2 Info
```

---

 0 **ALERT level A** = Most likely a serious problem - resolve or explain
 0 **ALERT level B** = A potentially serious problem, consider carefully
23 **ALERT level C** = Check. Ensure it is not caused by an omission or oversight
64 **ALERT level G** = General information/check it is not something unexpected

 7 ALERT type 1 CIF construction/syntax error, inconsistent or missing data
21 ALERT type 2 Indicator that the structure model may be wrong or deficient
 5 ALERT type 3 Indicator that the structure quality may be low
49 ALERT type 4 Improvement, methodology, query or suggestion
 5 ALERT type 5 Informative message, check

It is advisable to attempt to resolve as many as possible of the alerts in all categories. Often the minor alerts point to easily fixed oversights, errors and omissions in your CIF or refinement strategy, so attention to these fine details can be worthwhile. In order to resolve some of the more serious problems it may be necessary to carry out additional measurements or structure refinements. However, the purpose of your study may justify the reported deviations and the more serious of these should normally be commented upon in the discussion or experimental section of a paper or in the "special_details" fields of the CIF. checkCIF was carefully designed to identify outliers and unusual parameters, but every test has its limitations and alerts that are not important in a particular case may appear. Conversely, the absence of alerts does not guarantee there are no aspects of the results needing attention. It is up to the individual to critically assess their own results and, if necessary, seek expert advice.

Publication of your CIF in IUCr journals

A basic structural check has been run on your CIF. These basic checks will be run on all CIFs submitted for publication in IUCr journals (*Acta Crystallographica*, *Journal of Applied Crystallography*, *Journal of Synchrotron Radiation*); however, if you intend to submit to *Acta Crystallographica Section C* or *E* or *IUCrData*, you should make sure that full publication checks are run on the final version of your CIF prior to submission.

Publication of your CIF in other journals

Please refer to the *Notes for Authors* of the relevant journal for any special instructions relating to CIF submission.

PLATON version of 06/07/2023; check.def file version of 30/06/2023

Datablock 1 - ellipsoid plot

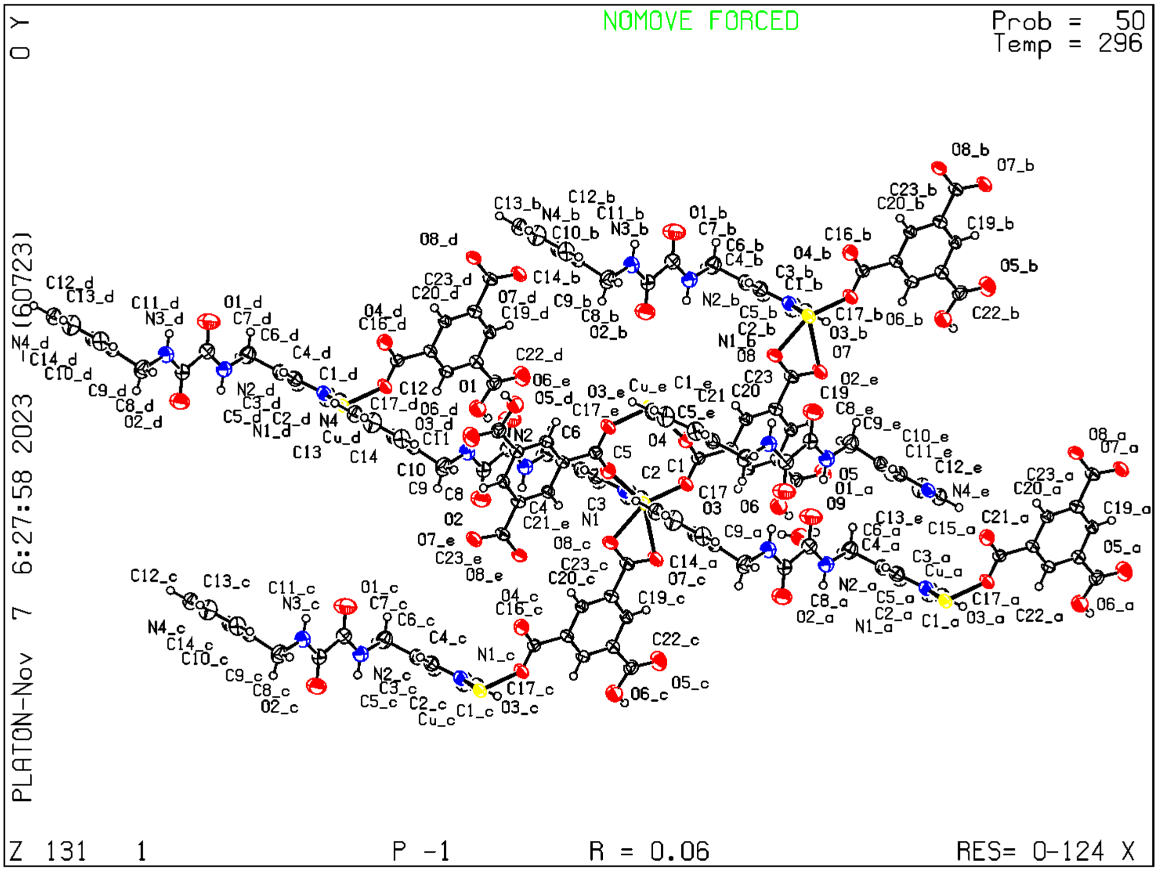

Datablock 2 - ellipsoid plot

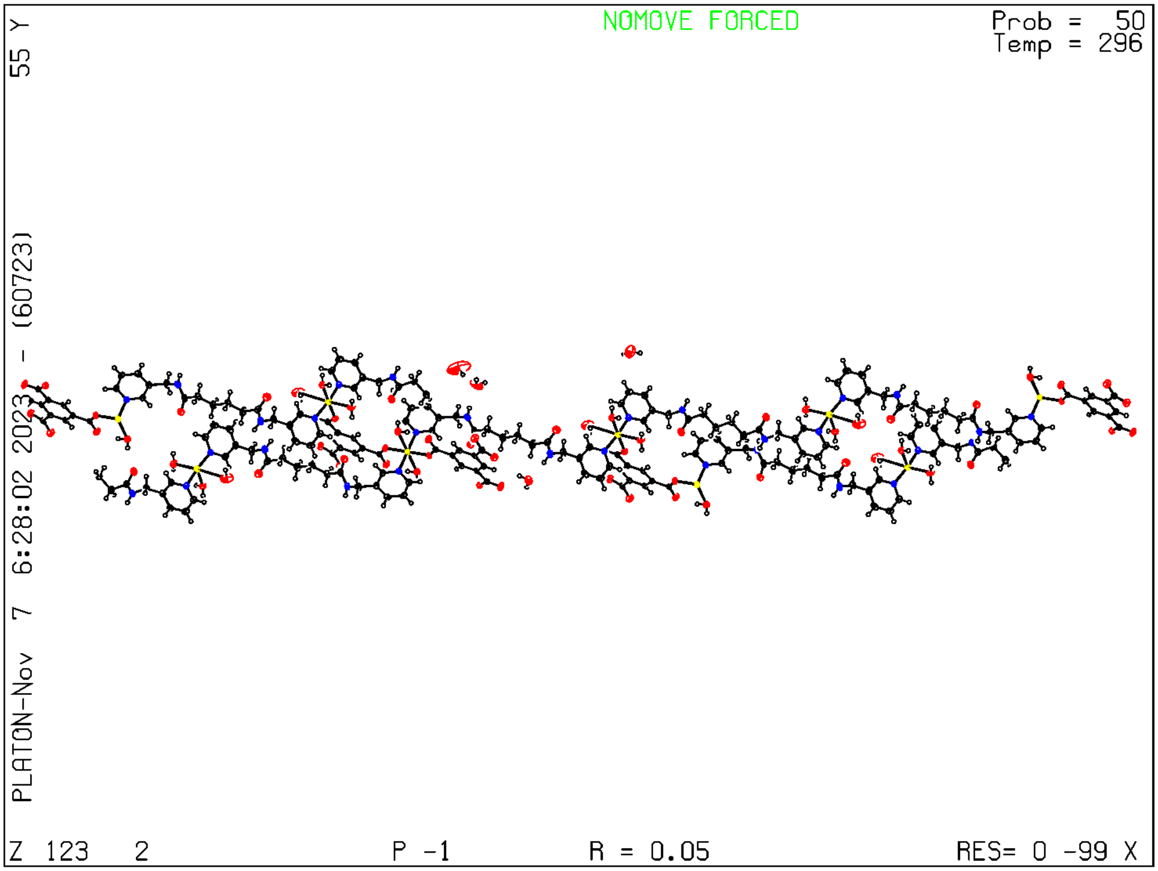

Datablock 3 - ellipsoid plot

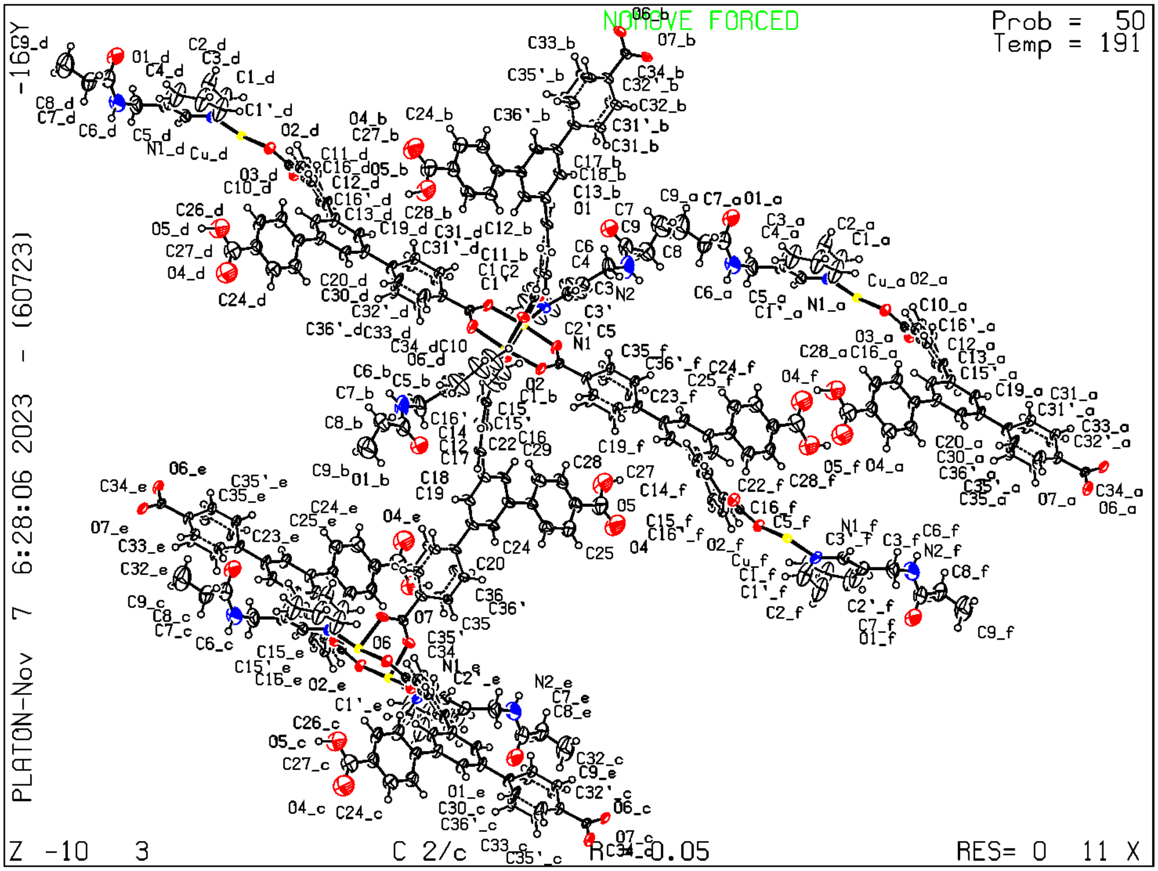

Datablock 4 - ellipsoid plot

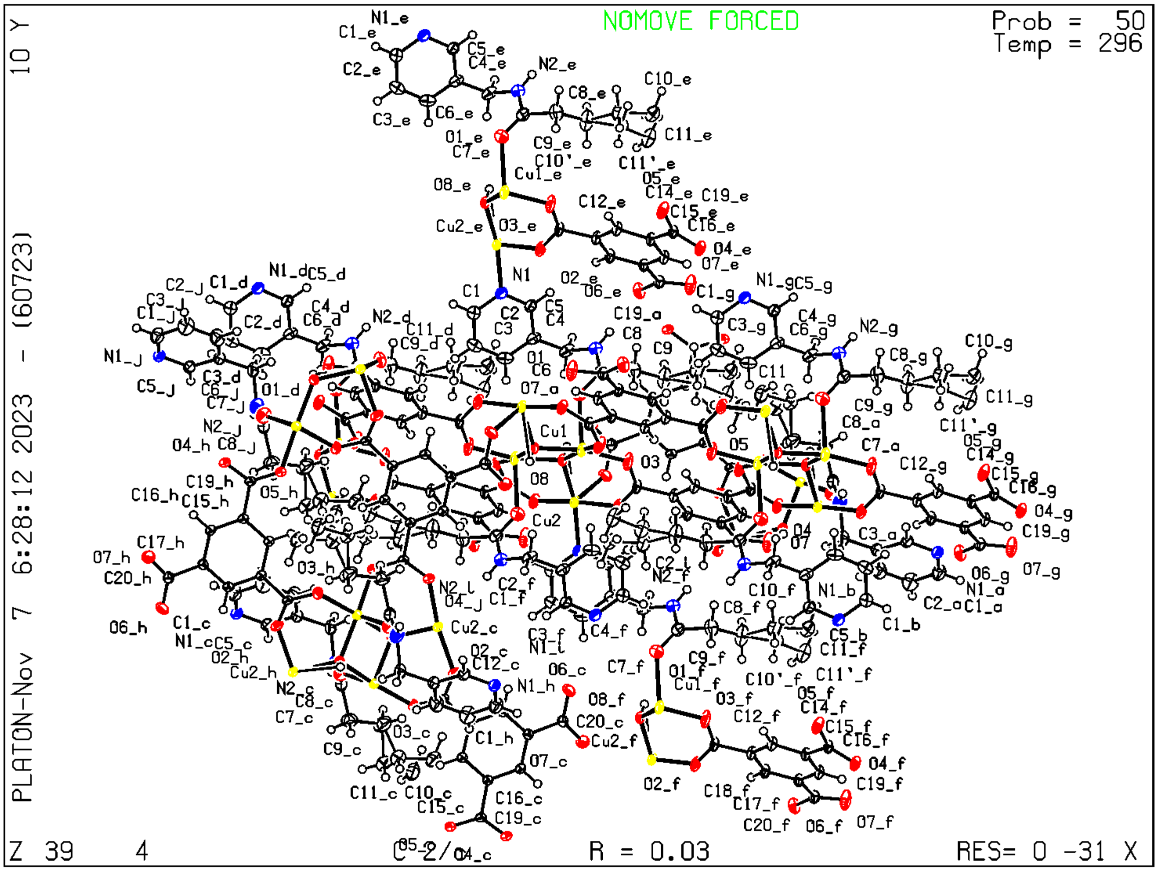

Datablock 5 - ellipsoid plot

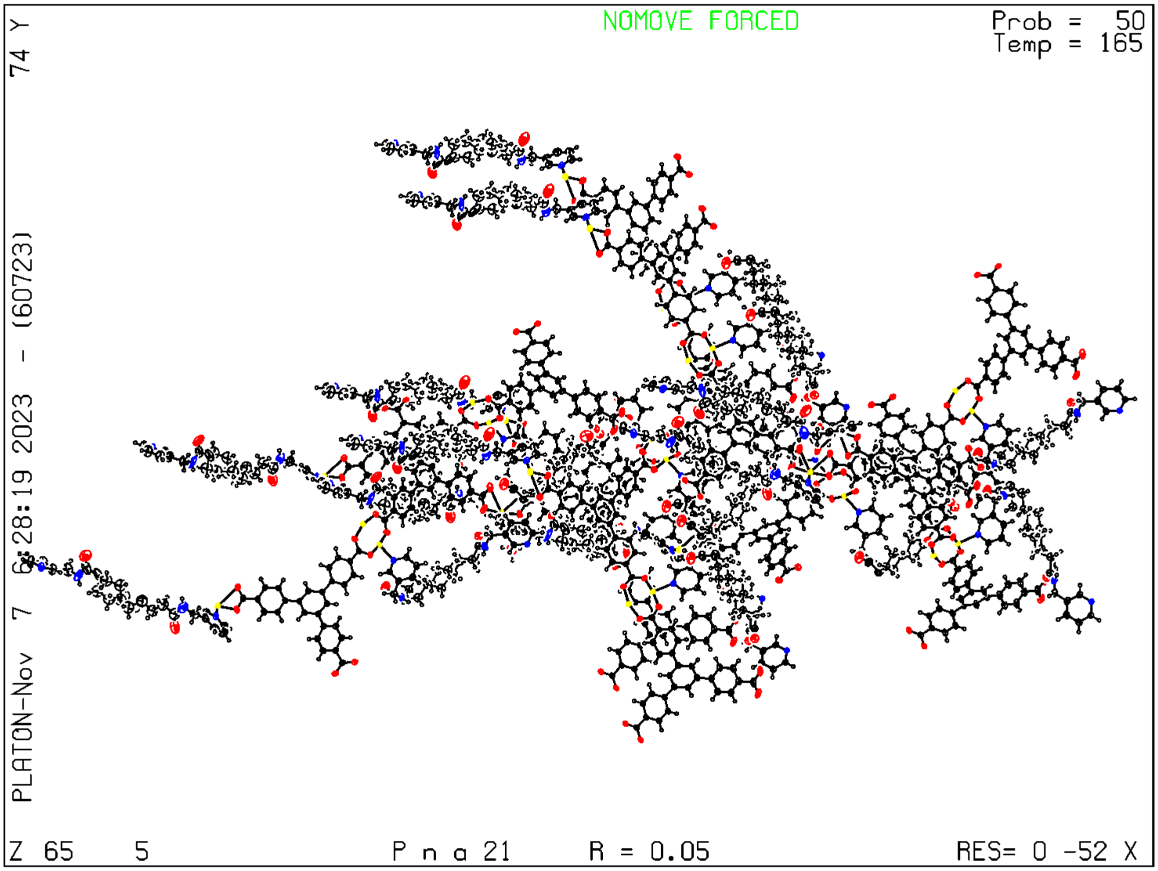

Datablock 6 - ellipsoid plot

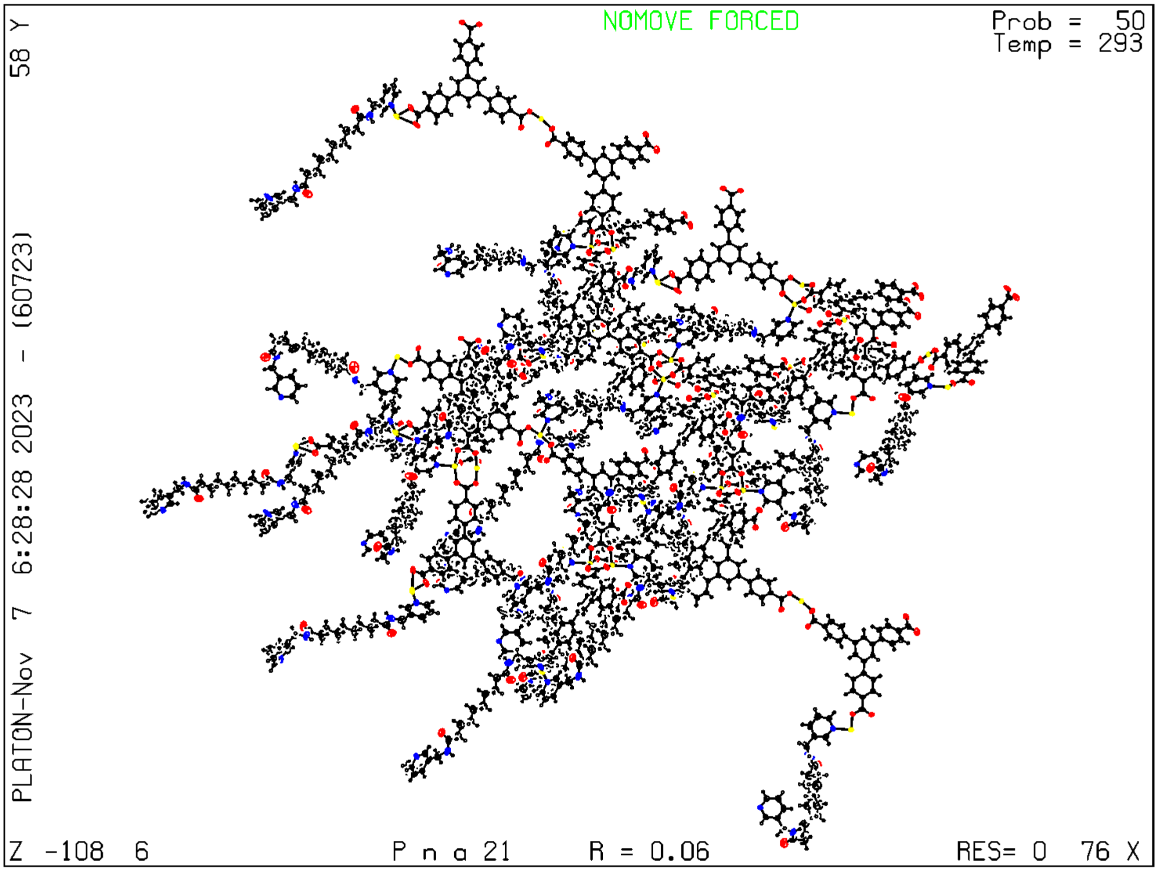

Supplement: Supplementary file 1 [file molecules-29-00311-s001.zip › checkcif.pdf]
