# Supplementary material for: Cu(II) Coordination Polymers Containing Mixed Ligands with Different Flexibilities: Structural Diversity and Iodine Adsorption
Source: Molecules. 2024 Jan 8;29(2):311. doi: 10.3390/molecules29020311 (PMC10819037; doi:10.3390/molecules29020311)

Supplementary materials

Shu-Yu Lin,[1] Yi-Lin Shen,[1] Wei-Hao Chen,[1]
Manivannan Govindaraj[2,*] and Jhy-Der Chen[1,*]

[1] Department of Chemistry, Chung Yuan Christian University, Chung Li, Taoyuan City 320, Taiwan;
[2] Department of Chemistry, Periyar Maniammai Institute of Science & Technology (Deemed to be University), Vallam, Thanjavur, 613 403, Tamil Nadu, India;
* Correspondence: manivannang@pmu.edu (M.G); jdchen@cycu.edu.tw (J.-D.C.); Tel.: +886-3-265-3351 (J.-D. C.)

Fig. S1. The infrared spectrum of complex **1**.

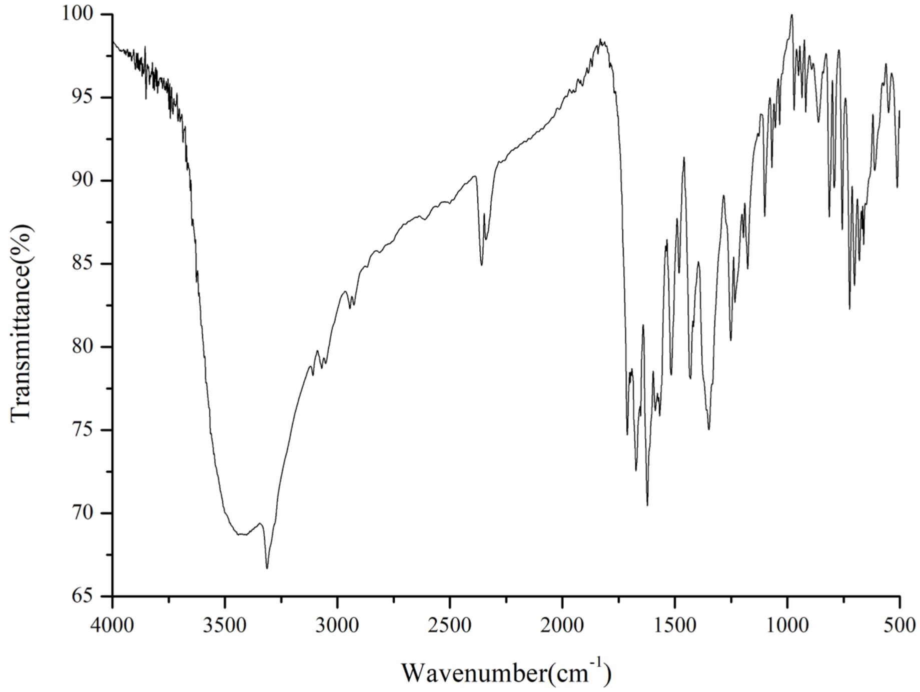

Fig. S2. The infrared spectrum of complex **2**.

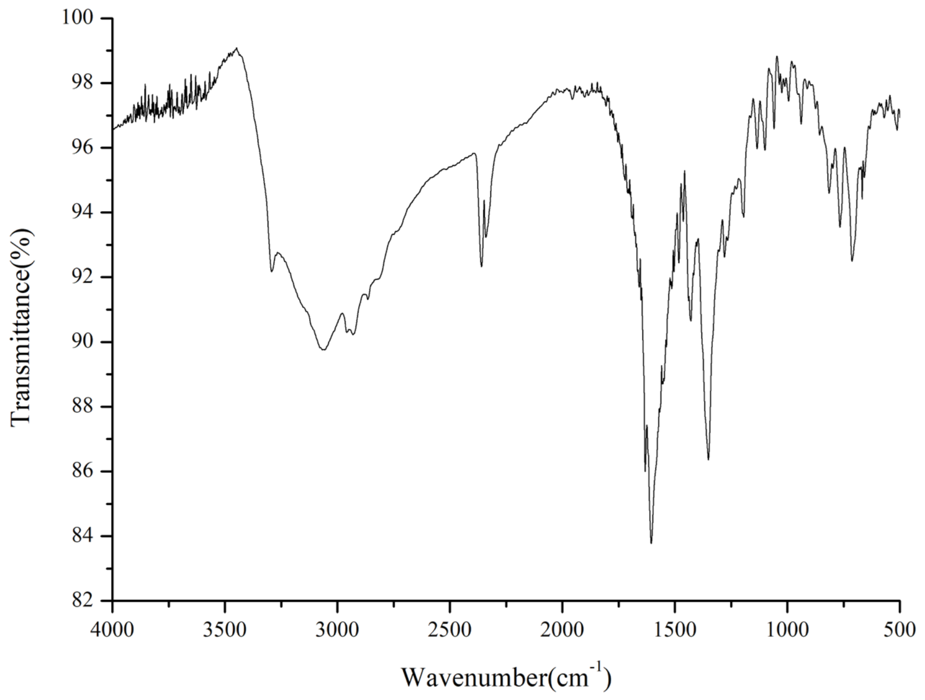

Fig. S3. The infrared spectrum of complex **3**.

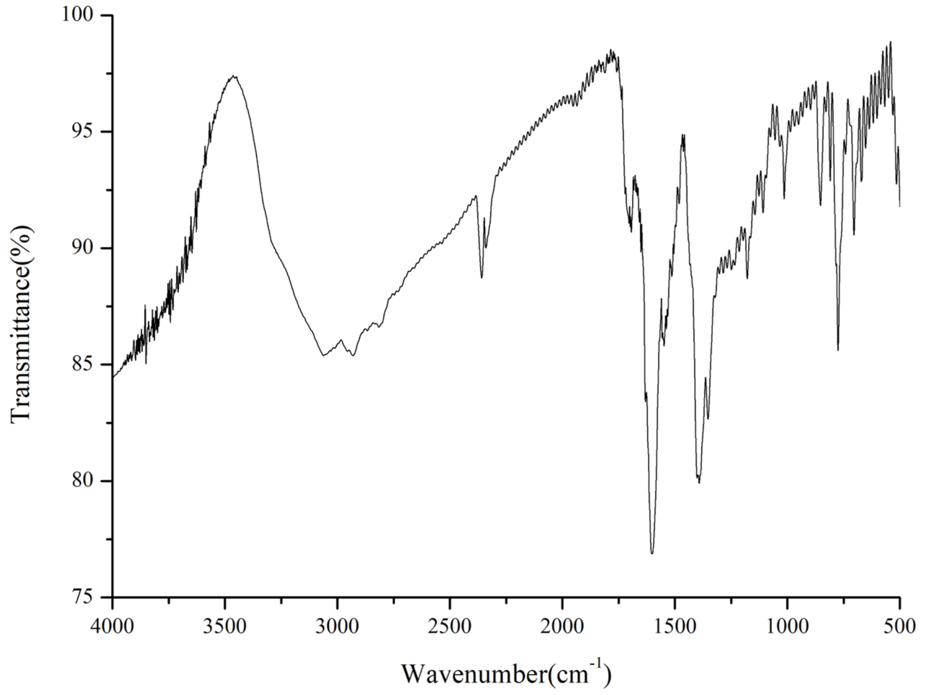

Fig. S4. The infrared spectrum of complex **4**.

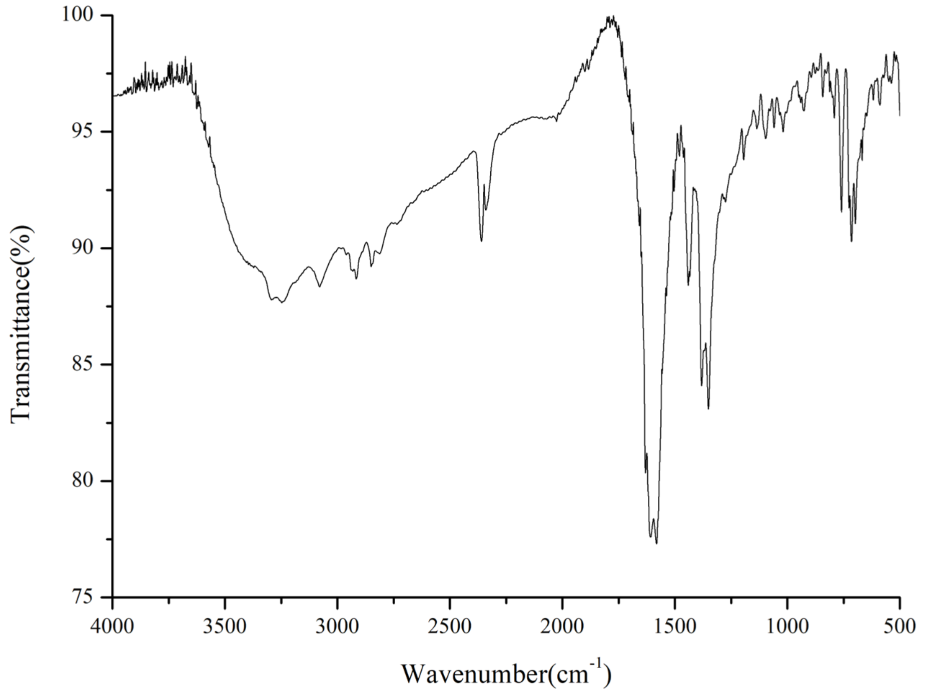

Fig. S5. The infrared spectrum of complex **5**.

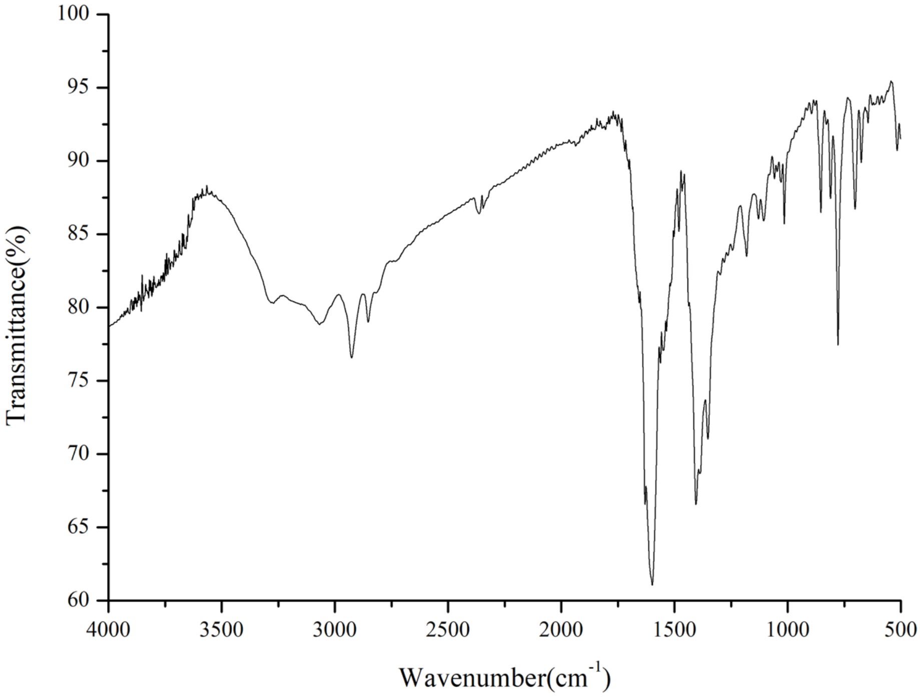

Fig. S6. The infrared spectrum of complex **6**.

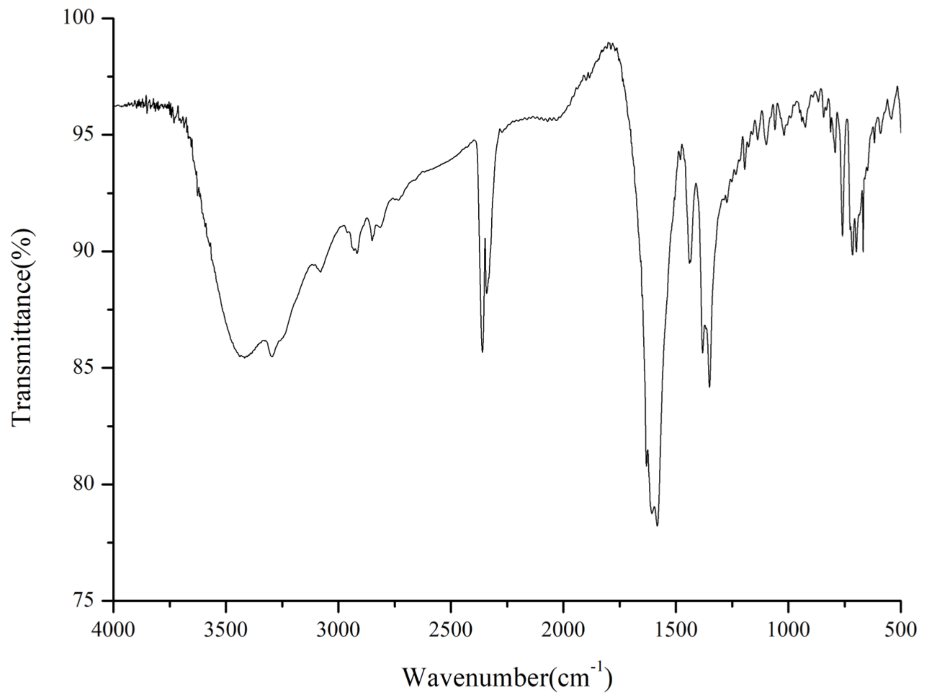

Fig. S7. Simulated and experimental PXRD patterns of **1**.

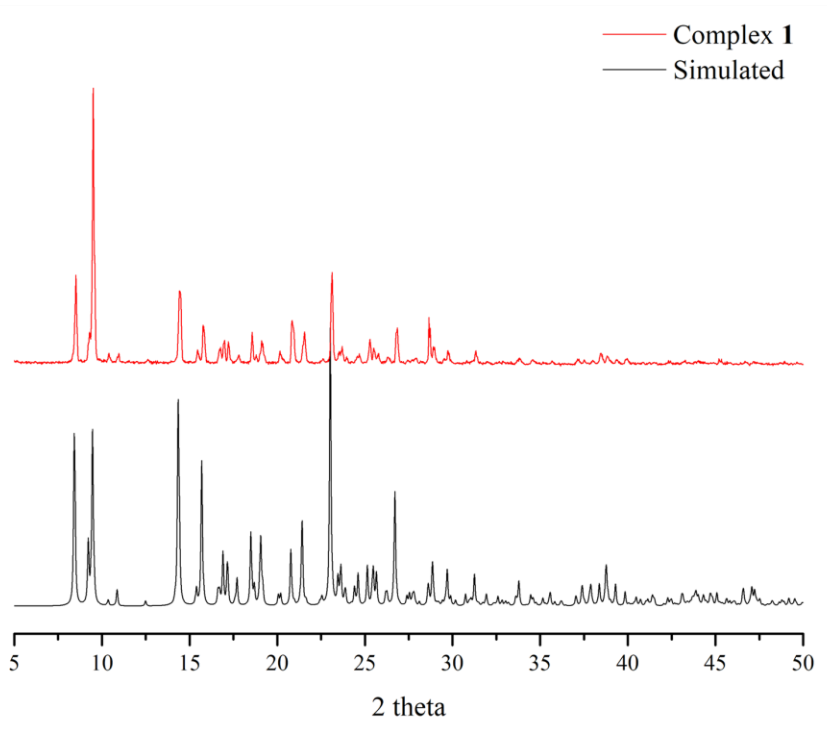

Fig. S8. Simulated and experimental PXRD patterns of **2.**

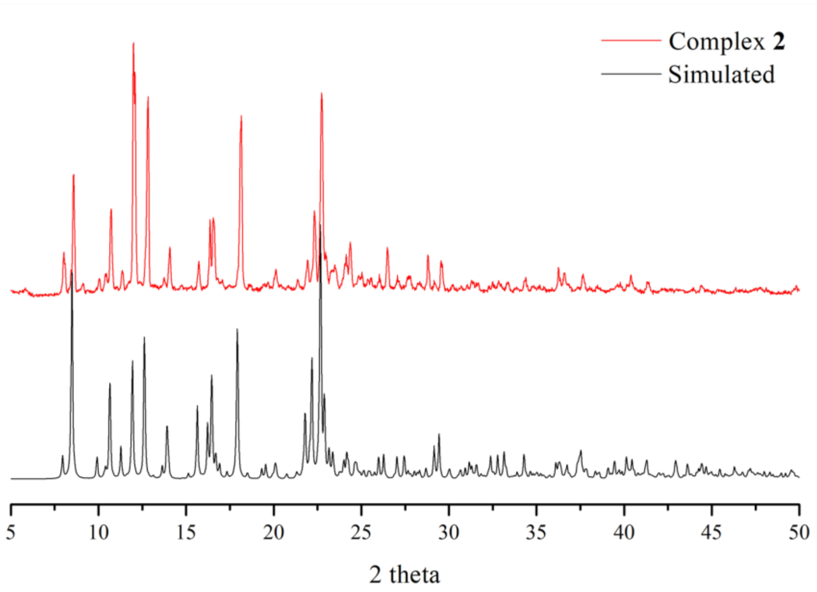

Fig. S9. Simulated and experimental PXRD patterns of **3**.

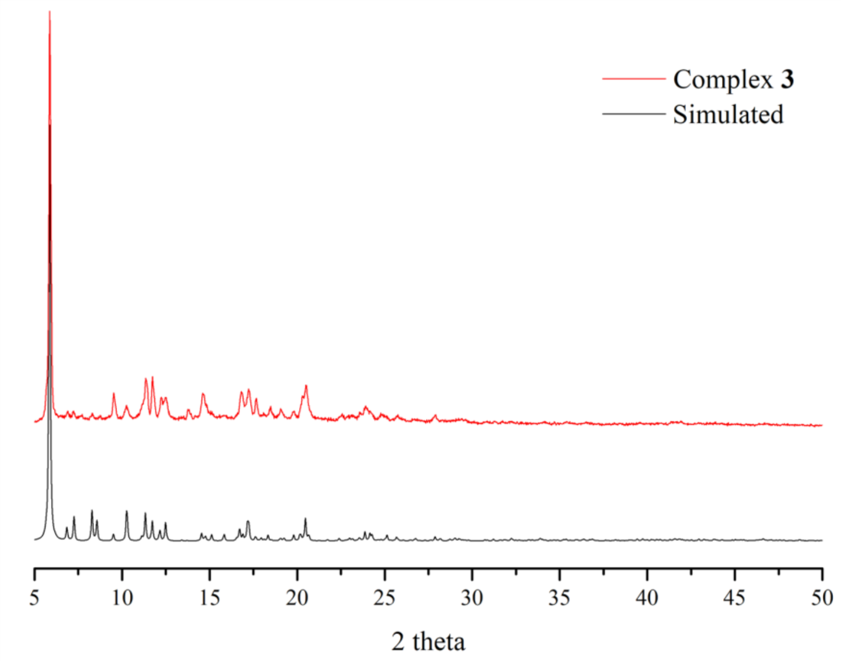

Fig. S10. Simulated and experimental PXRD patterns of **4**.

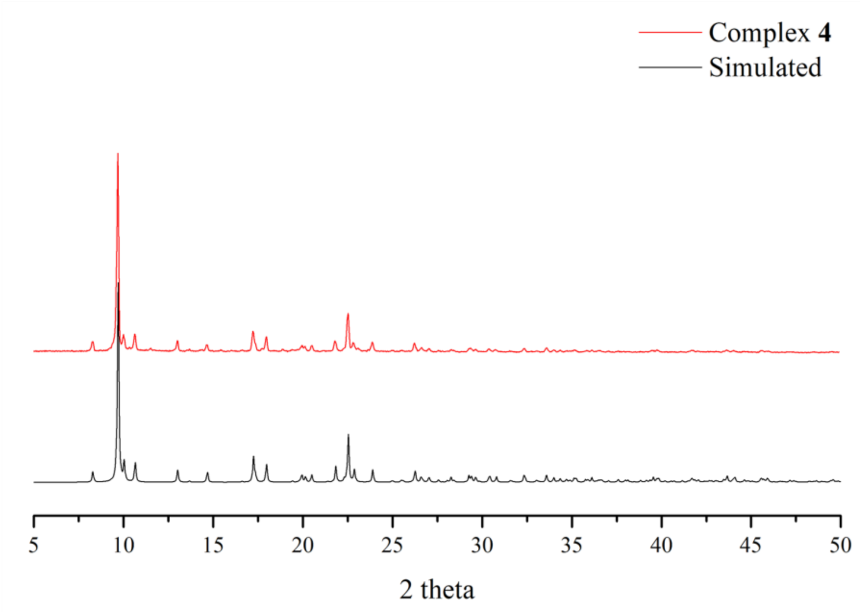

Fig. S11. Simulated and experimental PXRD patterns of **5**.

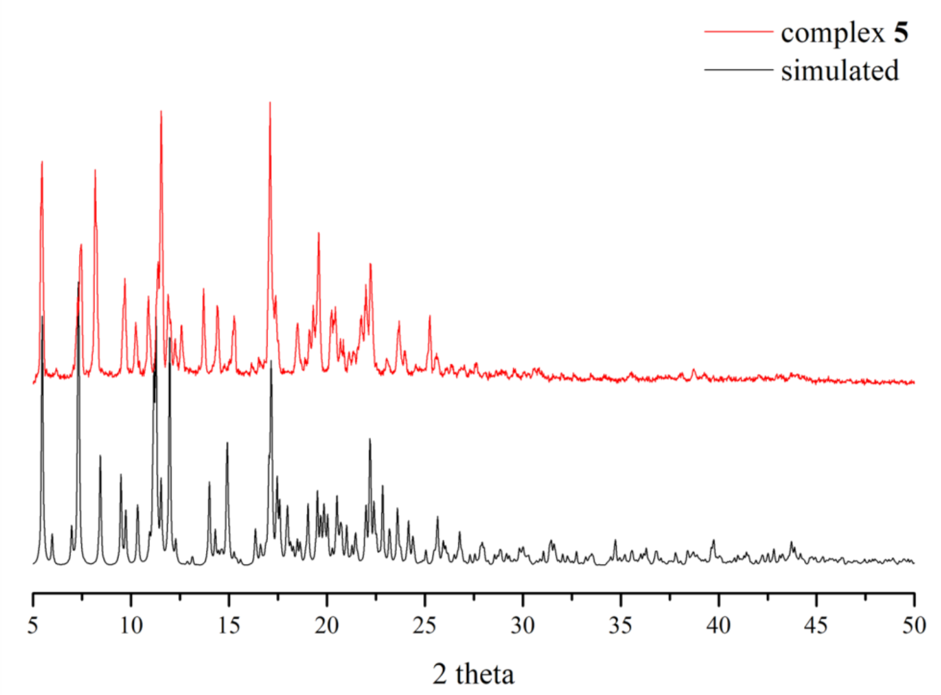

Fig. S12. Simulated and experimental PXRD patterns of **6**.

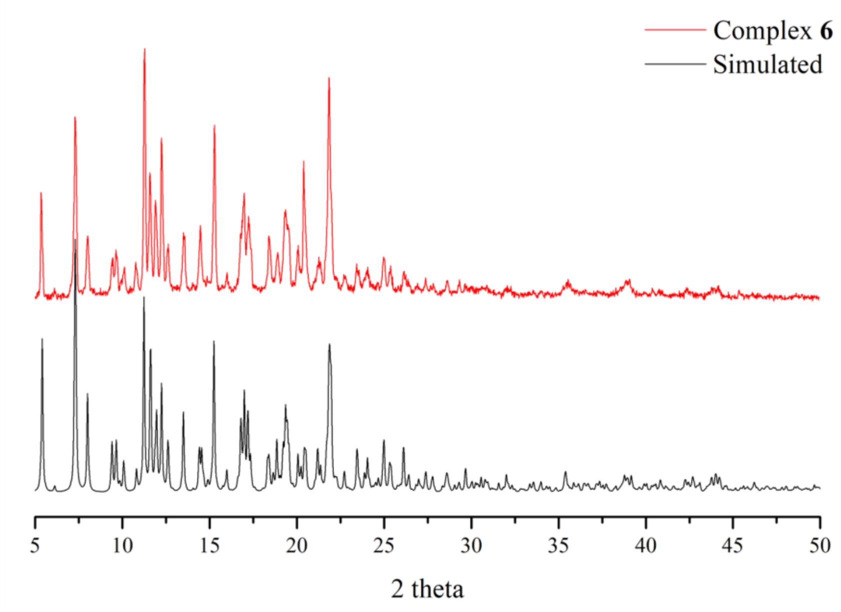

Fig. S13. The TGA curve for complex **1**.

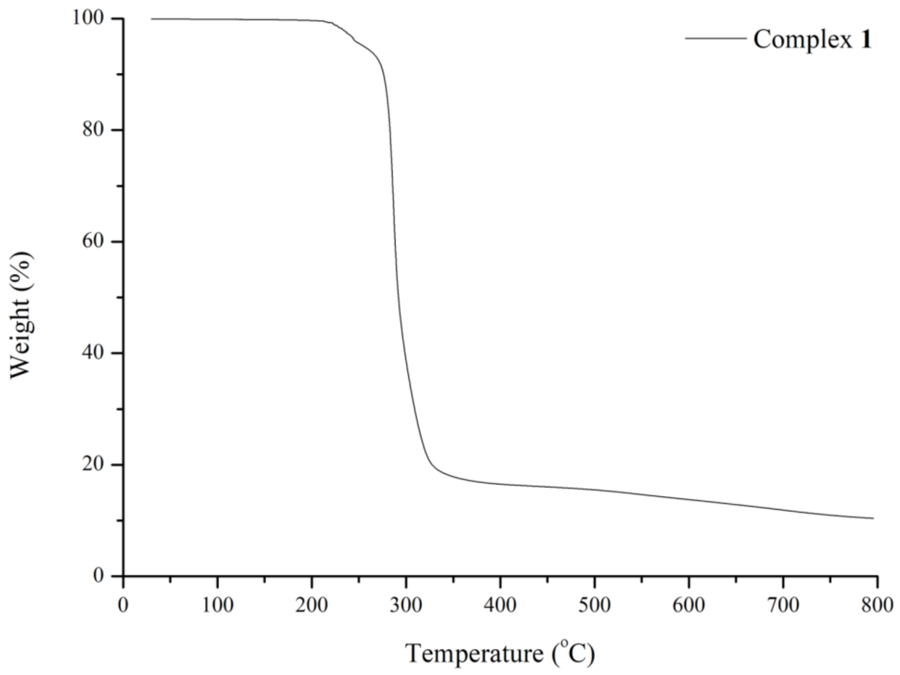

Fig. S14. The TGA curve for complex **2**.

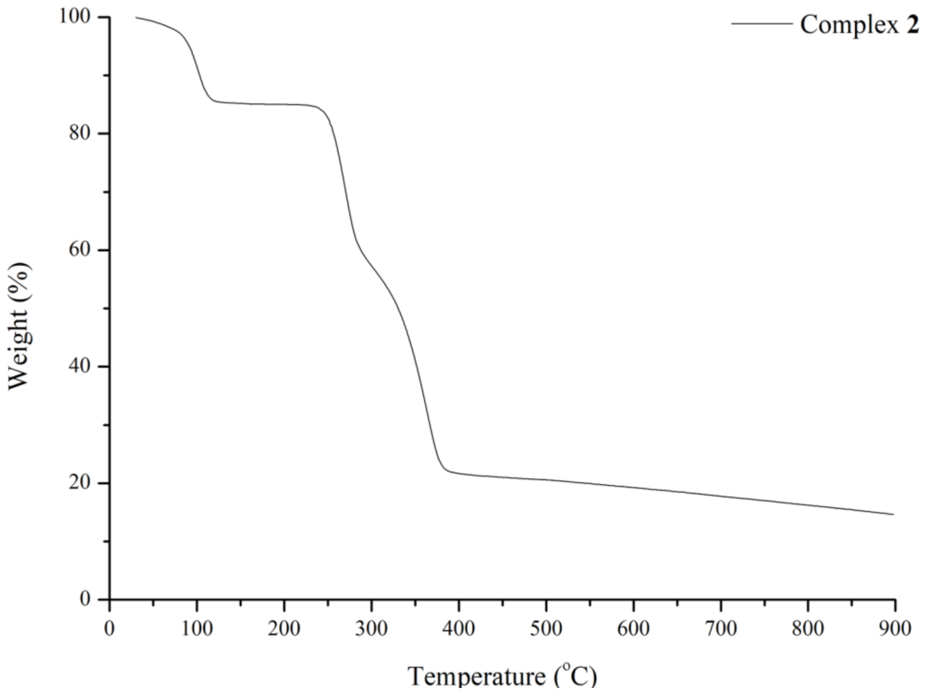

Fig. S15. The TGA curve for complex **3**.

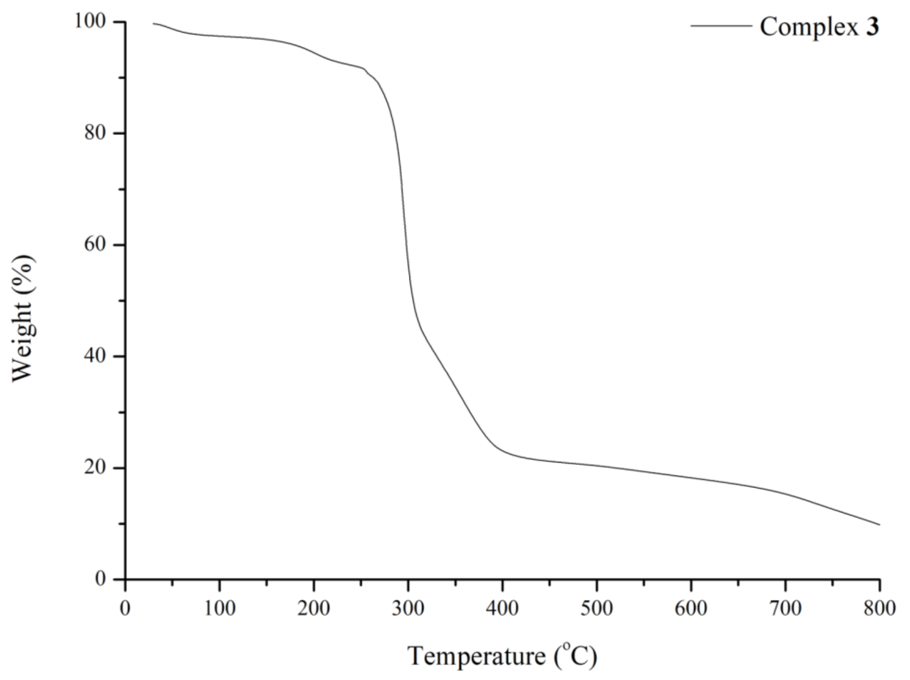

Fig. S16. The TGA curve for complex **4**.

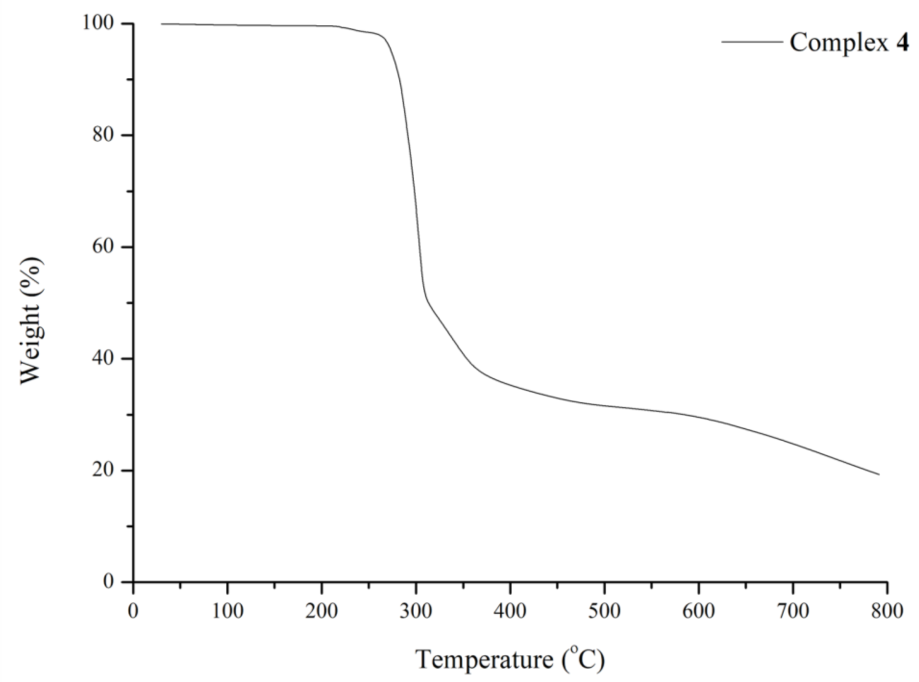

Fig. S17. The TGA curve for complex **5**.

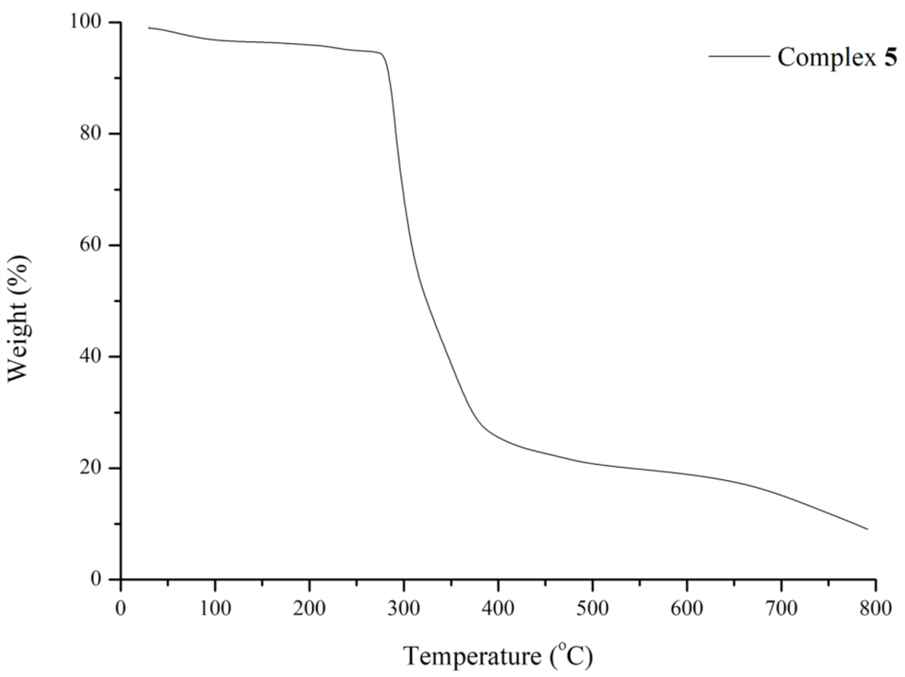

Fig. S18. The TGA curve for complex **6**.

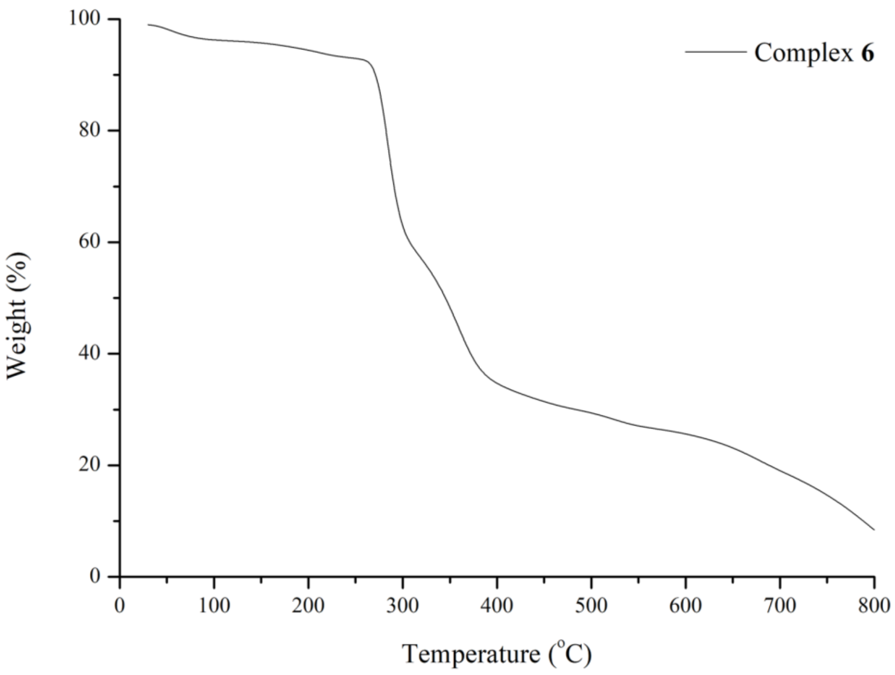

Fig. S19. Color change for the iodine-adsorbed samples of **1** at 25 °C. (a) Original **1**, and **1** adsorbed iodine for (b) 30 mins, (c) 60 mins, (d) 120 mins, (e) 180 mins and (f) 360 mins.

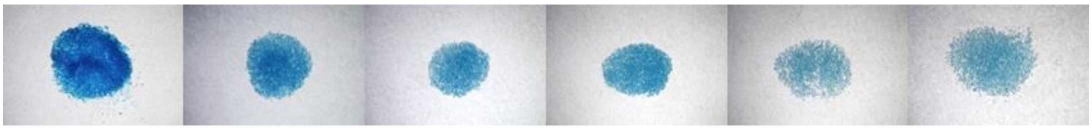

(a) (b) (c) (d) (e) (f)

Fig. S20. Color change for the iodine-adsorbed samples of **1** at 60 °C. (a) Original **1**, and **1** adsorbed iodine for (b) 30 mins, (c) 60 mins, (d) 120 mins, (e) 180 mins and (f) 360 mins.

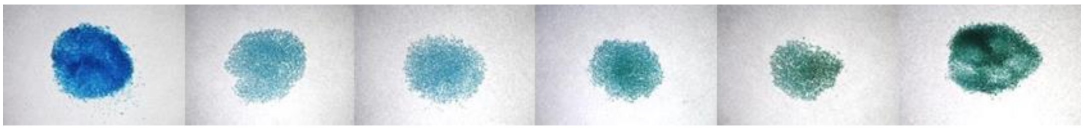

(a) (b) (c) (d) (e) (f)

Fig. S21. Color change for the iodine-adsorbed samples of **2** at 25 °C. (a) Original **2**, and **2** adsorbed iodine for (b) 30 mins, (c) 60 mins, (d) 120 mins, (e) 180 mins and (f) 360 mins.

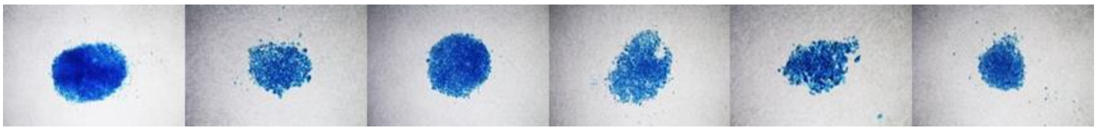

(a) (b) (c) (d) (e) (f)

Fig. S22. Color change for the iodine-adsorbed samples of **2** at 60 °C. (a) Original **2**, and **2** adsorbed iodine for (b) 30 mins, (c) 60 mins, (d) 120 mins, (e) 180 mins and (f) 360 mins.

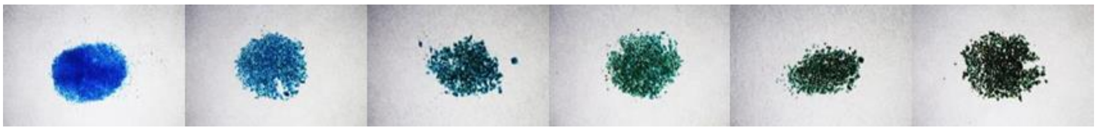

Fig. S23. Color change for the iodine-adsorbed samples of **3** at 25 °C. (a) Original **3**, and **3** adsorbed iodine for (b) 30 mins, (c) 60 mins, (d) 120 mins, (e) 180 mins and (f) 360 mins.

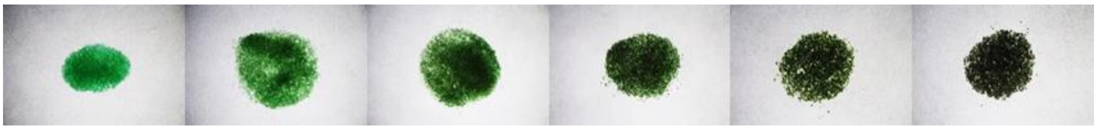

(a) (b) (c) (d) (e) (f)

Fig. S24. Color change for the iodine-adsorbed samples of **3** at 60 °C. (a) Original **3**, and **3** adsorbed iodine for (b) 30 mins, (c) 60 mins, (d) 120 mins, (e) 180 mins and (f) 360 mins.

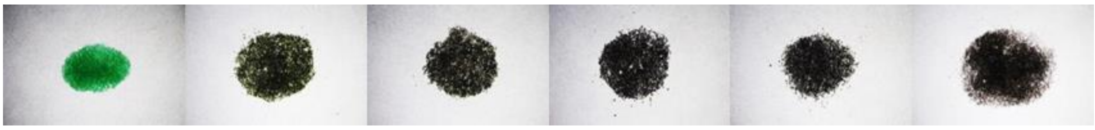

(a) (b) (c) (d) (e) (f)

Fig. S25. Color change for the iodine-adsorbed samples of **4** at 25 °C. (a) Original **4**, and **4** adsorbed iodine for (b) 30 mins, (c) 60 mins, (d) 120 mins, (e) 180 mins and (f) 360 mins.

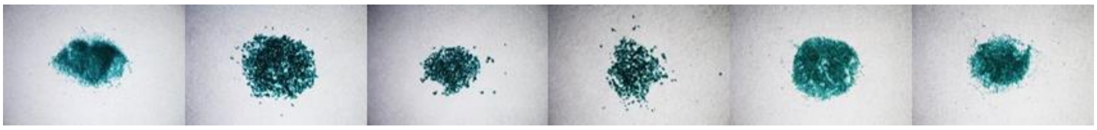

(a) (b) (c) (d) (e) (f)

Fig. S26. Color change for the iodine-adsorbed samples of **4** at 60 °C. (a) Original **4**, and **4** adsorbed iodine for (b) 30 mins, (c) 60 mins, (d) 120 mins, (e) 180 mins and (f) 360 mins.

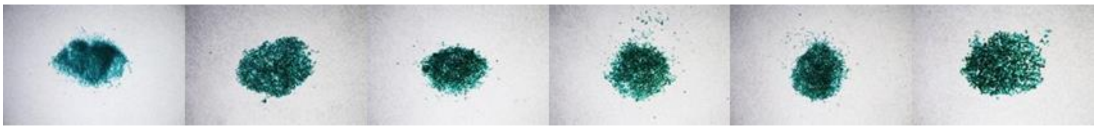

(a) (b) (c) (d) (e) (f)

Fig. S27. Color change for the iodine-adsorbed samples of **5** at 25 °C. (a) Original **5**, and **5** adsorbed iodine for (b) 30 mins, (c) 60 mins, (d) 120 mins, (e) 180 mins and (f) 360 mins.

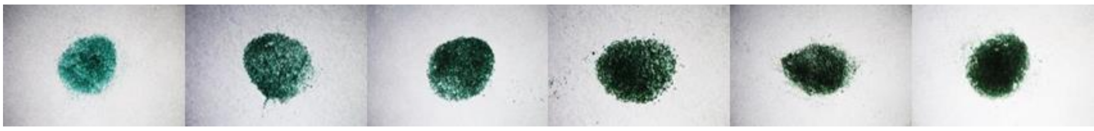

(a) (b) (c) (d) (e) (f)

Fig. S28. Color change for the iodine-adsorbed samples of **5** at 60 °C. (a) Original **5**, and **5** adsorbed iodine for (b) 30 mins, (c) 60 mins, (d) 120 mins, (e) 180 mins and (f) 360 mins.

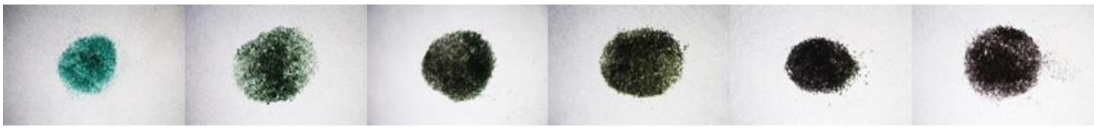

(a) (b) (c) (d) (e) (f)

Fig. S29. Color change for the iodine-adsorbed samples of **6** at 25 °C. (a) Original **6**, and **6** adsorbed iodine for (b) 30 mins, (c) 60 mins, (d) 120 mins, (e) 180 mins and (f) 360 mins.

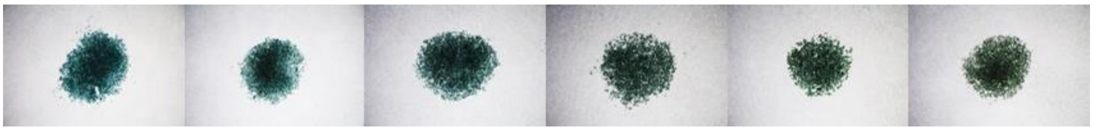

(a) (b) (c) (d) (e) (f)

Fig. S30. Color change for the iodine-adsorbed samples of **6** at 60 °C. (a) Original **6**, and **6** adsorbed iodine for (b) 30 mins, (c) 60 mins, (d) 120 mins, (e) 180 mins and (f) 360 mins.

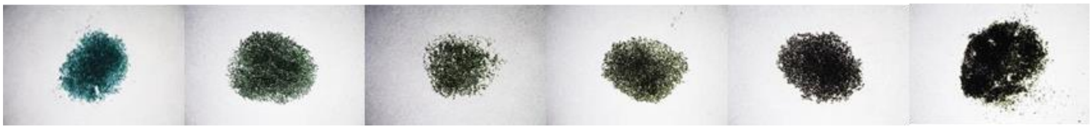

(a) (b) (c) (d) (e) (f)

Table S1. Iodine adsorption for complex **1** at 25 °C.

adsorption times (mins)	times	initial weight (mg)	final weight (mg)	iodine content (mg)	adsorbability (mg g^{-1})	average (mg g^{-1})
30	1	10.0	10.0	0.0	0.0	0.0
	2	10.0	10.0	0.0	0.0	
	3	10.0	10.0	0.0	0.0	
60	1	10.0	10.1	0.1	10.0	3.3
	2	10.0	10.0	0.0	0.0	
	3	10.0	10.0	0.0	0.0	
120	1	10.0	10.1	0.1	10.0	6.7
	2	10.0	10.1	0.1	10.0	
	3	10.0	10.0	0.0	0.0	
180	1	10.0	10.2	0.2	20.0	13.3
	2	10.0	10.1	0.1	10.0	
	3	10.0	10.1	0.1	10.0	
360	1	10.0	10.2	0.2	20.0	13.3
	2	10.0	10.1	0.1	10.0	
	3	10.0	10.1	0.1	10.0	

Table S2. Iodine adsorption for complex **1** at 60 °C.

adsorption times (mins)	times	initial weight (mg)	final weight (mg)	iodine content (mg)	adsorbability (mg g^{-1})	average (mg g^{-1})
30	1	10.0	10.0	0.0	0.0	0.0
	2	10.0	10.0	0.0	0.0	
	3	10.0	10.0	0.0	0.0	
60	1	10.0	10.1	0.1	10.0	10.0
	2	10.0	10.1	0.1	10.0	
	3	10.0	10.1	0.1	10.0	
120	1	10.0	10.2	0.2	20.0	13.3
	2	10.0	10.1	0.1	10.0	
	3	10.0	10.1	0.1	10.0	
180	1	10.0	10.2	0.2	20.0	16.7
	2	10.0	10.1	0.1	10.0	
	3	10.0	10.2	0.2	20.0	
360	1	10.0	10.2	0.2	20.0	20.0
	2	10.0	10.3	0.3	30.0	
	3	10.0	10.1	0.1	10.0	

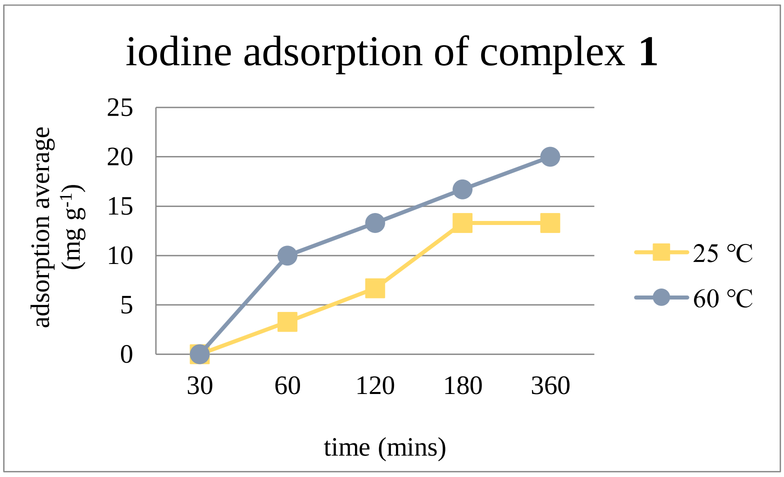

Table S3. Iodine adsorption for complex **2** at 25 °C.

adsorption times (mins)	times	initial weight (mg)	final weight (mg)	iodine content (mg)	adsorbability (mg g^{-1})	average (mg g^{-1})
30	1	10.0	10.1	0.1	10.0	3.3
	2	10.0	10.0	0.0	0.0	
	3	10.0	10.0	0.0	0.0	
60	1	10.0	10.1	0.1	10.0	10.0
	2	10.0	10.1	0.1	10.0	
	3	10.0	10.1	0.1	10.0	
120	1	10.0	10.1	0.1	10.0	13.3
	2	10.0	10.1	0.1	10.0	
	3	10.0	10.2	0.2	20.0	
180	1	10.0	10.1	0.1	10.0	13.3
	2	10.0	10.2	0.2	20.0	
	3	10.0	10.1	0.1	10.0	
360	1	10.0	10.4	0.4	40.0	33.3
	2	10.0	10.3	0.3	30.0	
	3	10.0	10.3	0.3	30.0	

Table S4. Iodine adsorption for complex **2** at 60 °C.

adsorption times (mins)	times	initial weight (mg)	final weight (mg)	iodine content (mg)	adsorbability (mg g^{-1})	average (mg g^{-1})
30	1	10.0	10.2	0.2	20.0	16.7
	2	10.0	10.1	0.1	10.0	
	3	10.0	10.2	0.2	20.0	
60	1	10.0	10.2	0.2	20.0	20.0
	2	10.0	10.3	0.3	30.0	
	3	10.0	10.1	0.1	10.0	
120	1	10.0	10.2	0.2	20.0	30.0
	2	10.0	10.4	0.4	40.0	
	3	10.0	10.3	0.3	30.0	
180	1	10.0	10.4	0.4	40.0	43.3
	2	10.0	10.5	0.5	50.0	
	3	10.0	10.4	0.4	40.0	
360	1	10.0	10.7	0.7	70.0	70.0
	2	10.0	10.8	0.8	80.0	
	3	10.0	10.6	0.6	60.0	

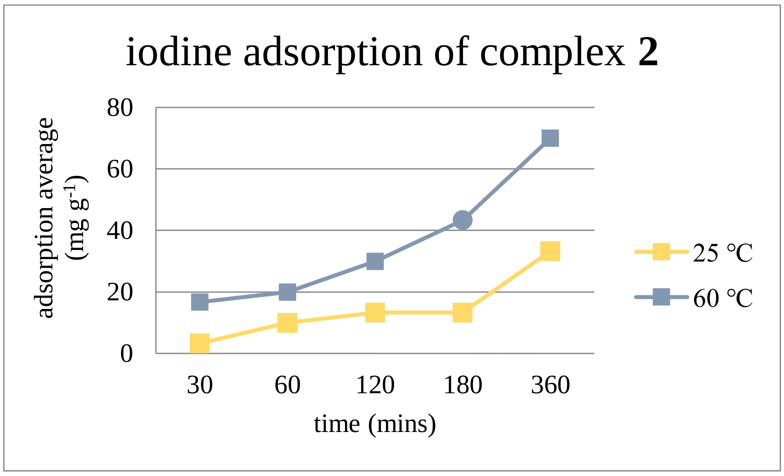

Table S5. Iodine adsorption for complex **3** at 25 °C.

adsorption times (mins)	times	initial weight (mg)	final weight (mg)	iodine content (mg)	adsorbability (mg g^{-1})	average (mg g^{-1})
	1	10.0	10.2	0.2	20.0	
30	2	10.0	10.1	0.1	10.0	13.3
	3	10.0	10.1	0.1	10.0	
	1	10.0	10.2	0.2	20.0	
60	2	10.0	10.1	0.1	10.0	13.3
	3	10.0	10.1	0.1	10.0	
	1	10.0	10.3	0.3	30.0	
120	2	10.0	10.2	0.2	20.0	30.0
	3	10.0	10.4	0.4	40.0	
	1	10.0	10.3	0.3	30.0	
180	2	10.0	10.3	0.3	30.0	30.0
	3	10.0	10.3	0.3	30.0	
	1	10.0	10.5	0.5	50.0	
360	2	10.0	10.4	0.4	40.0	46.7
	3	10.0	10.5	0.5	50.0	

Table S6. Iodine adsorption for complex **3** at 60 °C.

adsorption times (mins)	times	initial weight (mg)	final weight (mg)	iodine content (mg)	adsorbability (mg g^{-1})	average (mg g^{-1})
	1	10.0	10.1	0.1	10.0	
30	2	10.0	10.2	0.2	20.0	26.7
	3	10.0	10.5	0.5	50.0	
	1	10.0	10.2	0.2	20.0	
60	2	10.0	10.3	0.3	30.0	36.7
	3	10.0	10.6	0.6	60.0	
	1	10.0	11.3.	1.3	130.0	
120	2	10.0	10.8	0.8	80.0	93.3
	3	10.0	10.7	0.7	70.0	
	1	10.0	11.3	1.3	130.0	
180	2	10.0	11.0	1.0	100.0	116.7
	3	10.0	11.2	1.2	120.0	
	1	10.0	11.5	1.5	150.0	
360	2	10.0	11.5	1.5	150.0	156.7
	3	10.0	11.7	1.7	170.0	

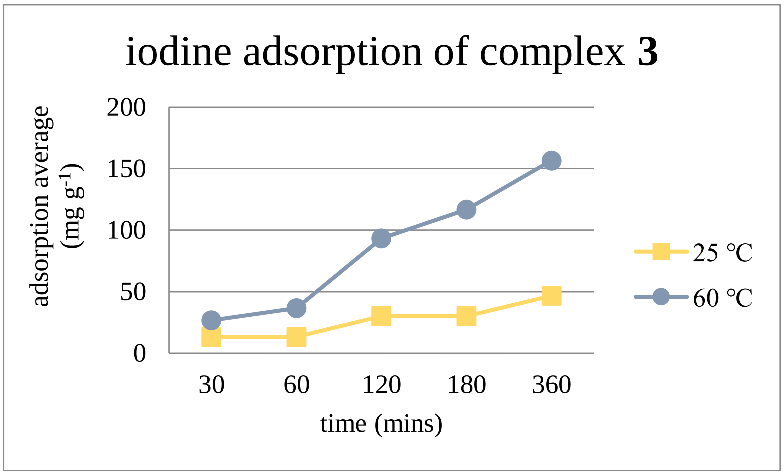

Table S7. Iodine adsorption for complex **4** at 25 °C.

adsorption times (mins)	times	initial weight (mg)	final weight (mg)	iodine content (mg)	adsorbability (mg g^{-1})	average (mg g^{-1})
	1	10.0	10.1	0.1	10.0	
30	2	10.0	10.0	0.0	0.0	3.3
	3	10.0	10.0	0.0	0.0	
	1	10.0	10.1	0.1	10.0	
60	2	10.0	10.0	0.0	0.0	6.7
	3	10.0	10.1	0.1	10.0	
	1	10.0	10.2	0.2	20.0	
120	2	10.0	10.2	0.2	20.0	20.0
	3	10.0	10.2	0.2	20.0	
	1	10.0	10.2	0.2	20.0	
180	2	10.0	10.2	0.2	20.0	20.0
	3	10.0	10.2	0.2	20.0	
	1	10.0	10.2	0.2	20.0	
360	2	10.0	10.2	0.2	20.0	20.0
	3	10.0	10.2	0.2	20.0	

Table S8. Iodine adsorption for complex **4** at 60 °C.

adsorption times (mins)	times	initial weight (mg)	final weight (mg)	iodine content (mg)	adsorbability (mg g^{-1})	average (mg g^{-1})
30	1	10.0	10.1	0.1	10.0	10.0
	2	10.0	10.0	0.0	0.0	
	3	10.0	10.2	0.2	20.0	
60	1	10.0	10.2	0.2	20.0	20.0
	2	10.0	10.1	0.1	10.0	
	3	10.0	10.3	0.3	30.0	
120	1	10.0	10.4	0.4	40.0	26.7
	2	10.0	10.3	0.3	30.0	
	3	10.0	10.1	0.1	10.0	
180	1	10.0	10.4	0.4	40.0	26.7
	2	10.0	10.1	0.1	10.0	
	3	10.0	10.3	0.3	30.0	
360	1	10.0	10.4	0.4	40.0	40.0
	2	10.0	10.5	0.5	50.0	
	3	10.0	10.3	0.3	30.0	

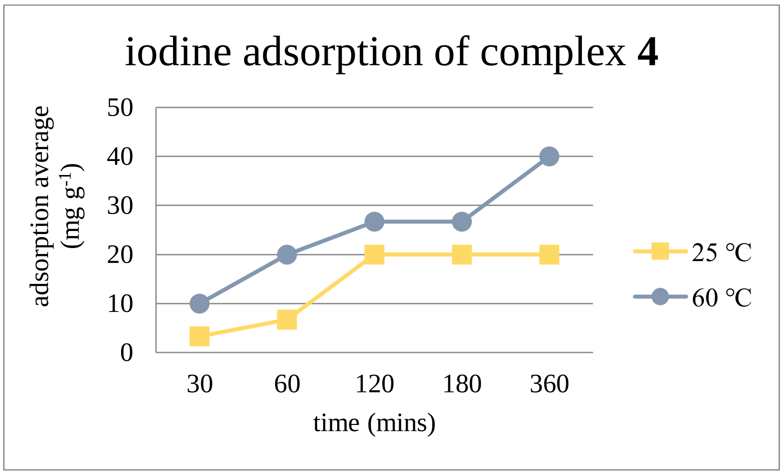

Table S9. Iodine adsorption for complex **5** at 25 °C.

adsorption times (mins)	times	initial weight (mg)	final weight (mg)	iodine content (mg)	adsorbability (mg g^{-1})	average (mg g^{-1})
	1	10.0	10.2	0.2	20.0	
30	2	10.0	10.0	0.0	0.0	10.0
	3	10.0	10.1	0.1	10.0	
	1	10.0	10.2	0.2	20.0	
60	2	10.0	10.1	0.1	10.0	16.7
	3	10.0	10.2	0.2	20.0	
	1	10.0	10.3	0.3	30.0	
120	2	10.0	10.1	0.1	10.0	20.0
	3	10.0	10.2	0.2	20.0	
	1	10.0	10.3	0.3	30.0	
180	2	10.0	10.2	0.2	20.0	30.0
	3	10.0	10.4	0.4	40.0	
	1	10.0	10.4	0.4	40.0	
360	2	10.0	10.3	0.3	30.0	36.7
	3	10.0	10.4	0.4	40.0	

Table S10. Iodine adsorption for complex **5** at 60 °C.

adsorption times (mins)	times	initial weight (mg)	final weight (mg)	iodine content (mg)	adsorbability (mg g^{-1})	average (mg g^{-1})
30	1	10.0	10.7	0.7	70.0	73.3
	2	10.0	10.5	0.5	50.0	
	3	10.0	11.0	1.0	100.0	
60	1	10.0	11.4	1.4	140.0	133.3
	2	10.0	11.3	1.3	130.0	
	3	10.0	11.3	1.3	130.0	
120	1	10.0	12.2	2.2	220.0	190.0
	2	10.0	11.9	1.9	190.0	
	3	10.0	11.6	1.6	160.0	
180	1	10.0	12.2	2.2	220.0	193.3
	2	10.0	11.8	1.8	180.0	
	3	10.0	11.8	1.8	180.0	
360	1	10.0	12.9	2.9	290.0	290.0
	2	10.0	13.0	3.0	300.0	
	3	10.0	12.8	2.8	280.0	

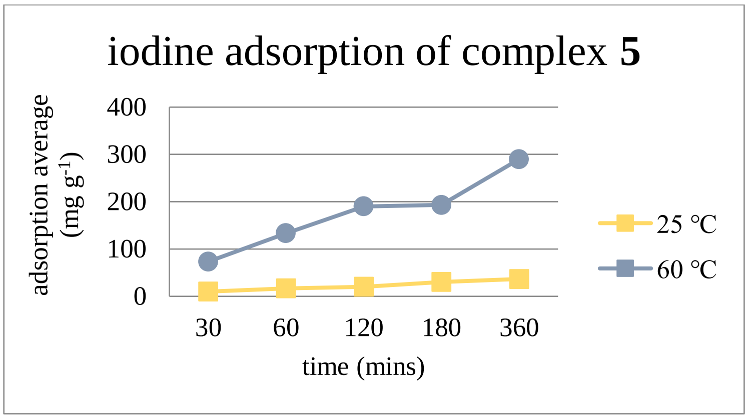

Table S11. Iodine adsorption for complex **6** at 25 °C.

adsorption times (mins)	times	initial weight (mg)	final weight (mg)	iodine content (mg)	adsorbability (mg g^{-1})	average (mg g^{-1})
30	1	10.0	10.0	0.0	0.0	0.0
	2	10.0	10.0	0.0	0.0	
	3	10.0	10.0	0.0	0.0	
60	1	10.0	10.1	0.1	10.0	10.0
	2	10.0	10.1	0.1	10.0	
	3	10.0	10.1	0.1	10.0	
120	1	10.0	10.4	0.4	40.0	33.3
	2	10.0	10.3	0.3	30.0	
	3	10.0	10.3	0.3	30.0	
180	1	10.0	10.4	0.4	40.0	40.0
	2	10.0	10.5	0.5	50.0	
	3	10.0	10.3	0.3	30.0	
360	1	10.0	10.7	0.7	70.0	53.3
	2	10.0	10.5	0.5	50.0	
	3	10.0	10.4	0.4	40.0	

Table S12. Iodine adsorption for complex **6** at 60 °C.

adsorption times (mins)	times	initial weight (mg)	final weight (mg)	iodine content (mg)	adsorbability (mg g^{-1})	average (mg g^{-1})
30	1	10.0	10.5	0.5	50.0	53.3
	2	10.0	10.2	0.2	20.0	
	3	10.0	10.9	0.9	90.0	
60	1	10.0	10.9	0.9	90.0	100.0
	2	10.0	11.0	1.0	100.0	
	3	10.0	11.1	1.1	110.0	
120	1	10.0	11.9	1.9	190.0	180.0
	2	10.0	11.6	1.6	160.0	
	3	10.0	11.9	1.9	190.0	
180	1	10.0	11.9	1.9	190.0	206.7
	2	10.0	12.3	2.3	230.0	
	3	10.0	12.0	2.0	200.0	
360	1	10.0	12.3	2.3	230.0	243.3
	2	10.0	12.5	2.5	250.0	
	3	10.0	12.5	2.5	250.0	

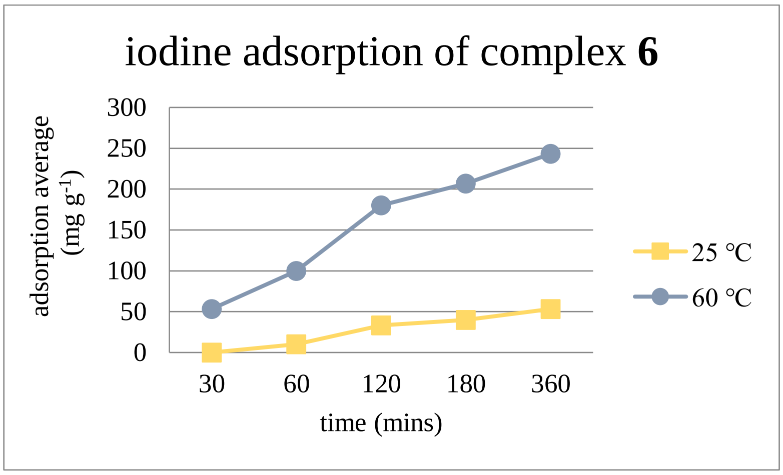

Fig. S31. PXRD patterns for **1** after iodine adsorption at 25 °C.

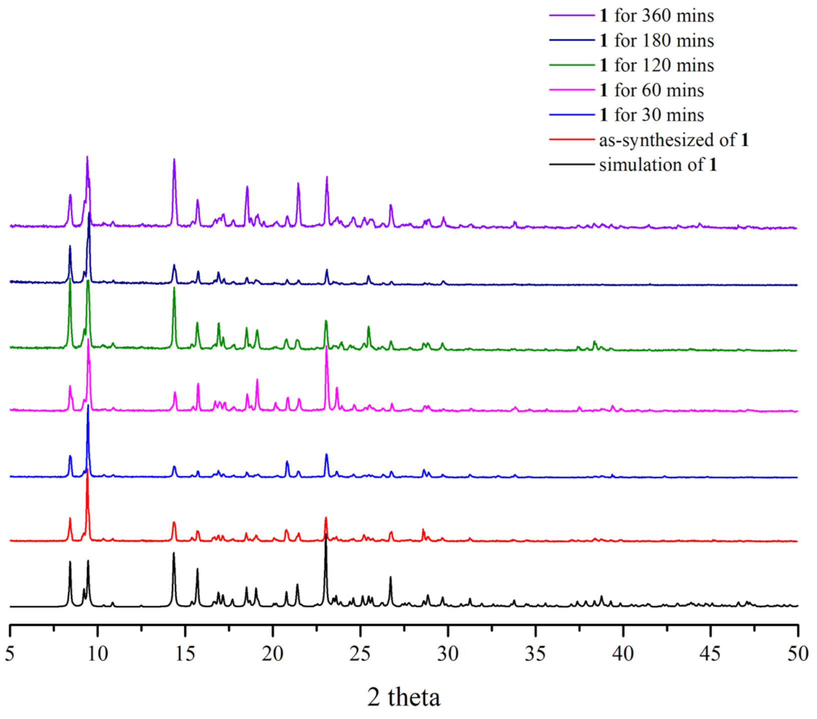

Fig. S32. PXRD patterns for **1** after iodine adsorption at 60 °C.

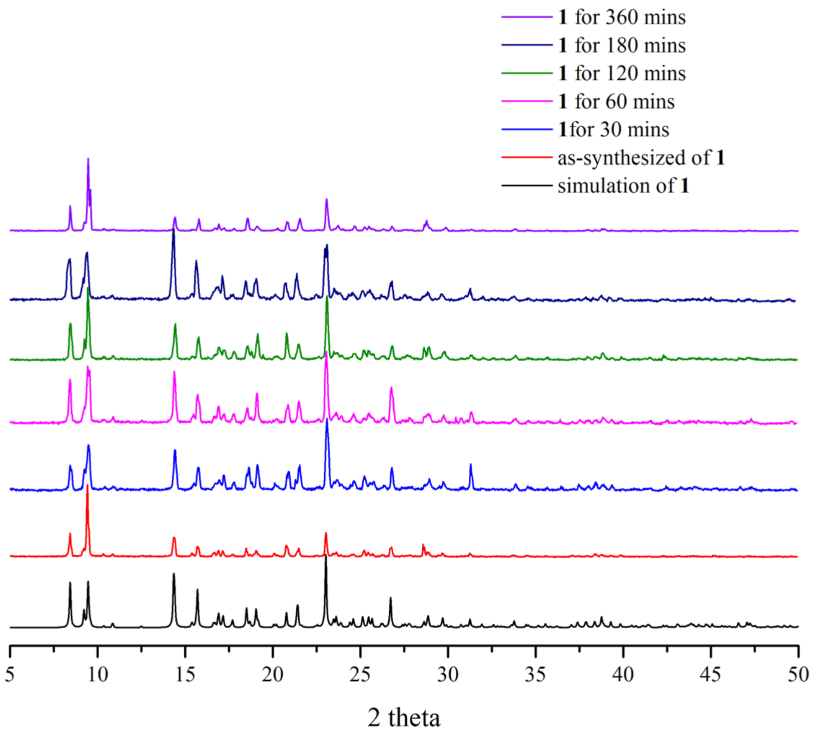

Fig. S33. PXRD patterns for **2** after iodine adsorption at 25 °C.

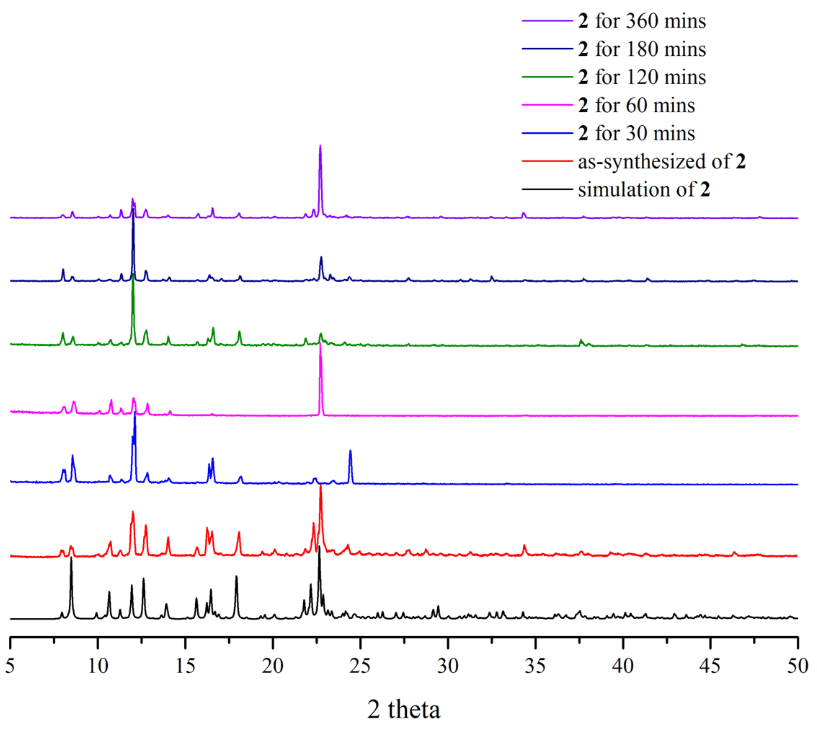

Fig. S34. PXRD patterns for **2** after iodine adsorption at 60 °C.

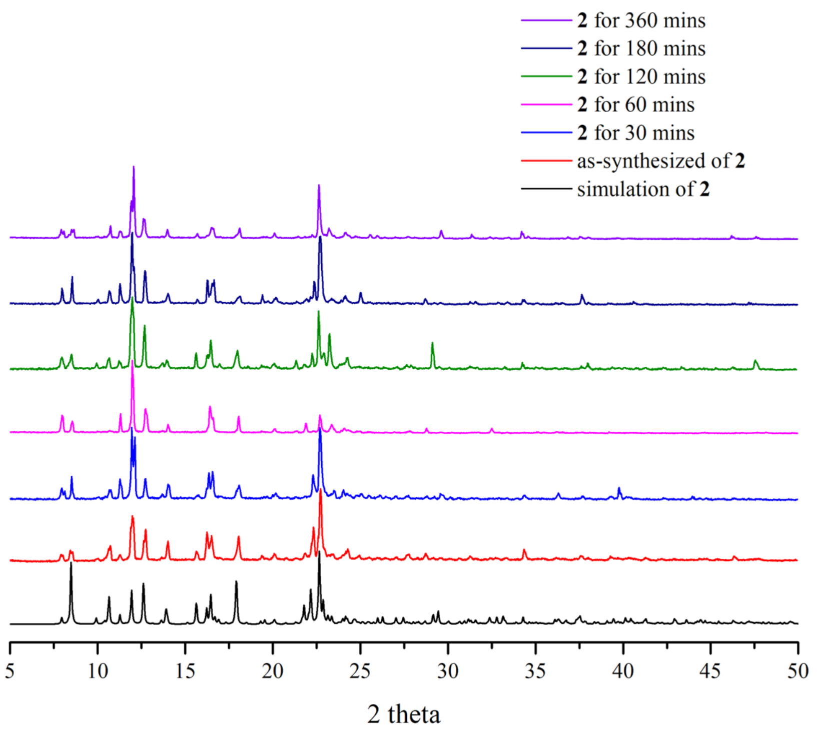

Fig. S35. PXRD patterns for **3** after iodine adsorption at 25 °C.

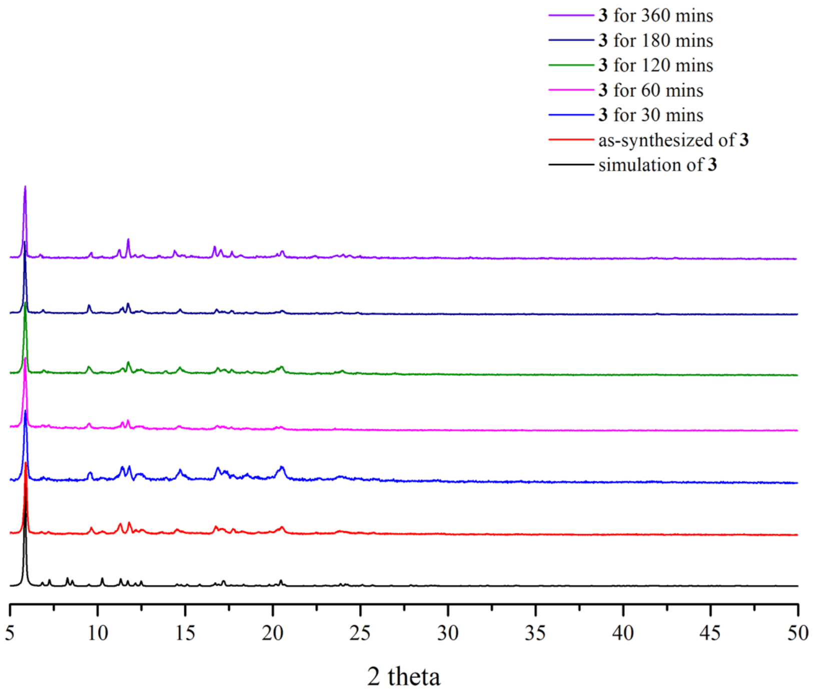

Fig. S36. PXRD patterns for **3** after iodine adsorption at 60 °C.

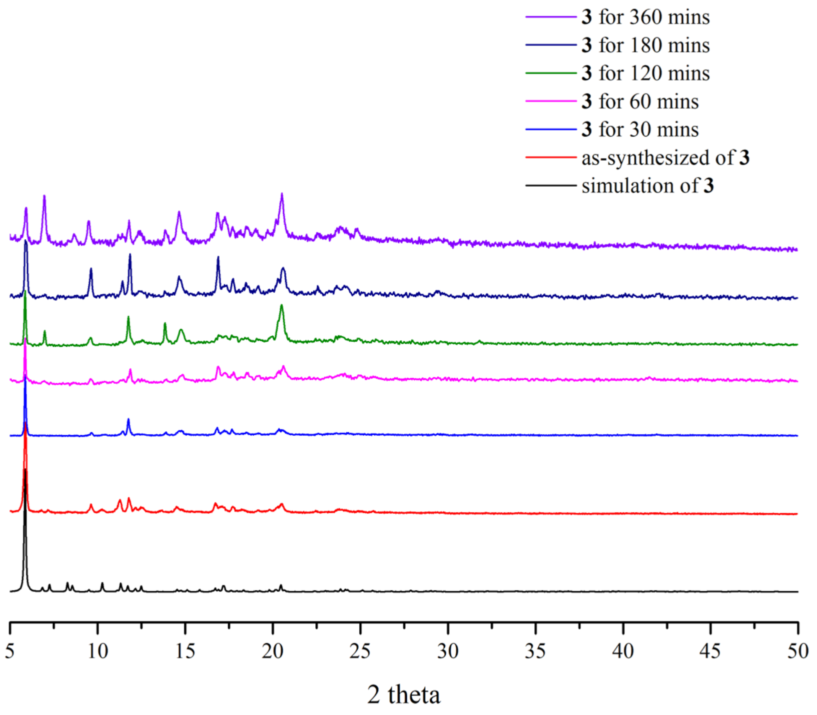

Fig. S37. PXRD patterns for **4** after iodine adsorption at 25 °C.

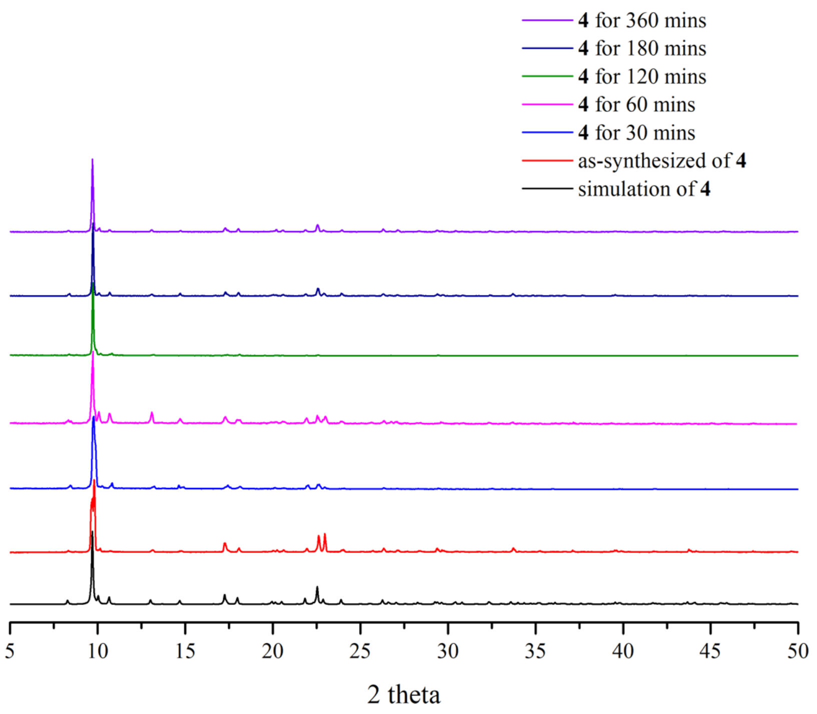

Fig. S38. PXRD patterns for **4** after iodine adsorption at 60 °C.

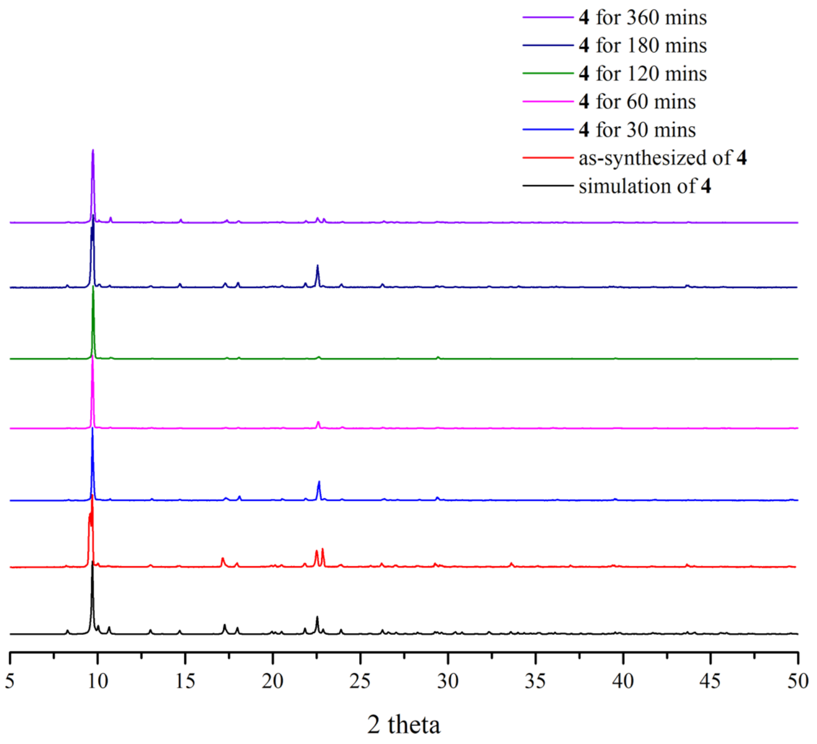

Fig. S39. PXRD patterns for **5** after iodine adsorption at 25 °C.

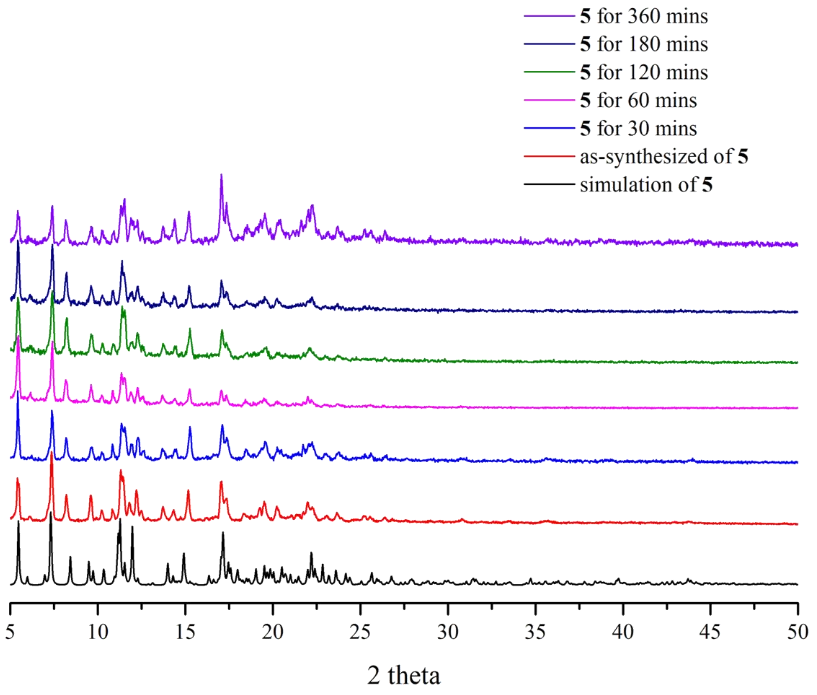

Fig. S40. PXRD patterns for **5** after iodine adsorption at 60 °C.

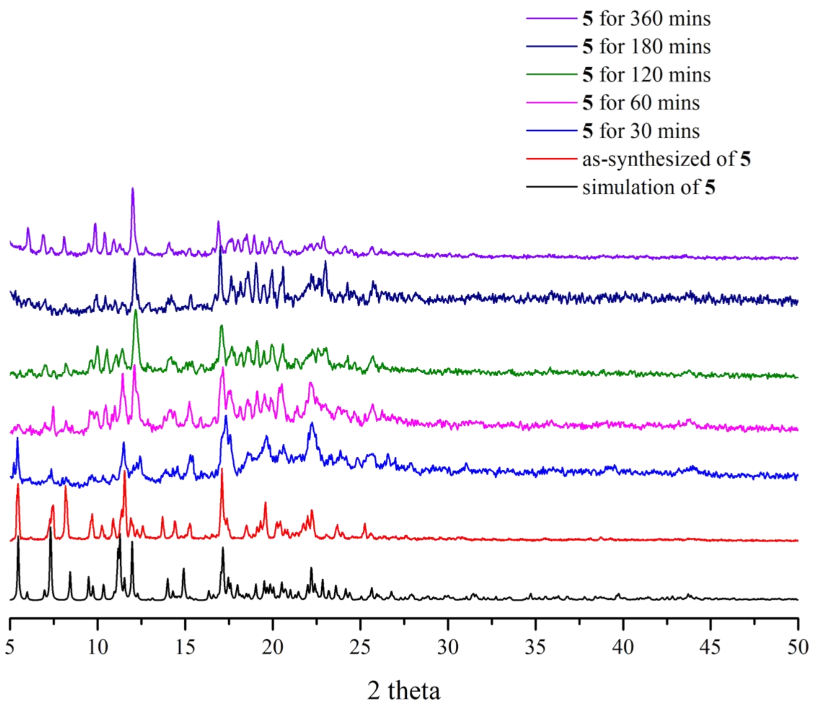

Fig. S41. PXRD patterns for **6** after iodine adsorption at 25 °C.

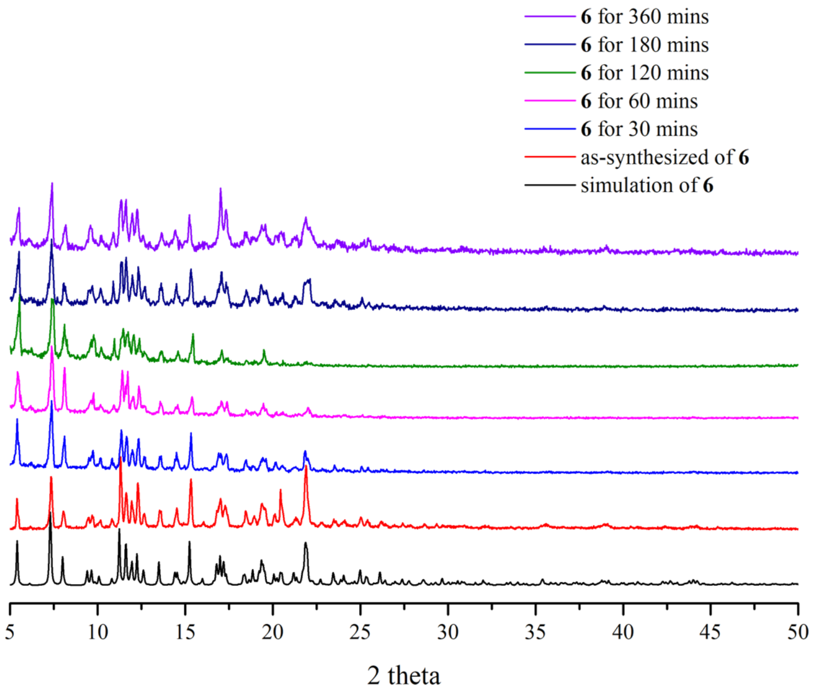

Fig. S42. PXRD patterns for **6** after iodine adsorption at 60 °C.

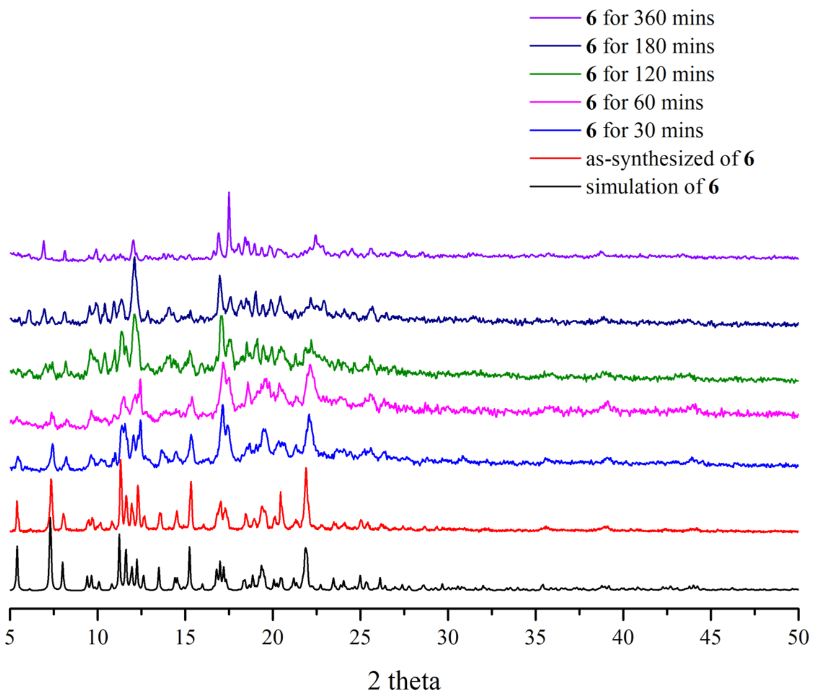

Fig. S43. EDX spectrum and data of complex **1**.

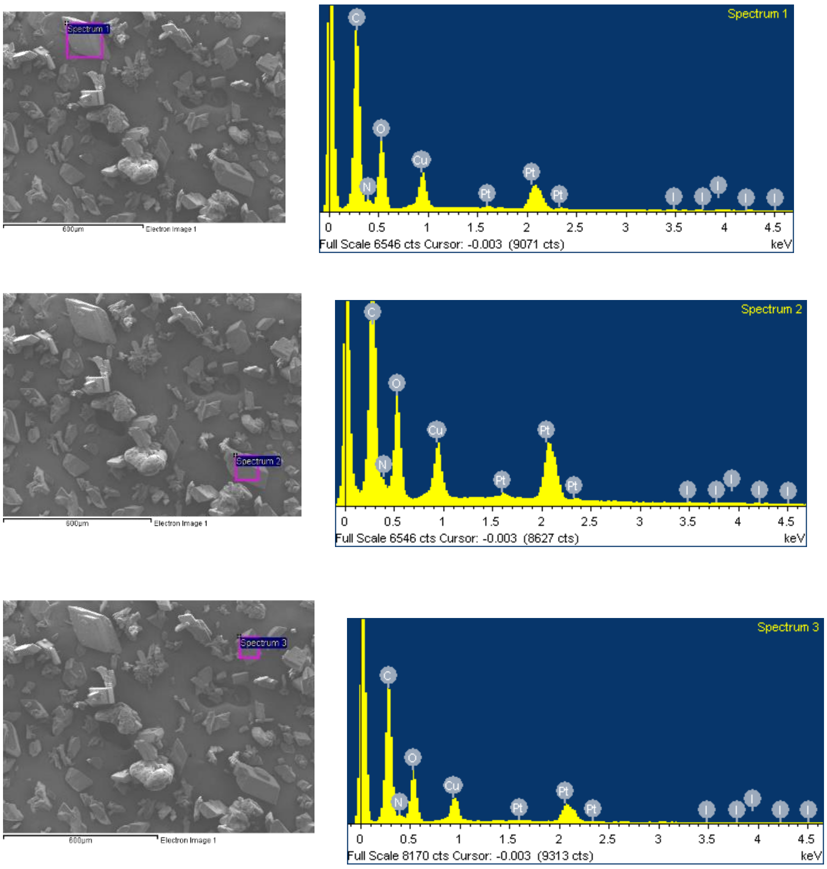

EDX data at three different positions for complex **1**.

	1st		2nd		3rd	
Element	Weight%	Atomic%	Weight%	Atomic%	Weight%	Atomic%
C K	52.76	65.02	48.48	59.19	54.73	66.59
O K	25.85	23.93	25.25	23.05	26.77	24.45
N K	7.57	8.01	13.97	14.50	5.95	6.20
Cu L	12.33	2.87	11.55	2.51	11.44	2.63
I L	1.49	0.17	0.75	0.75	1.11	0.13
Totals	100.00		100.00		100.00	

Average data

Element	Weight%	Atomic%
C K	51.99	63.60
O K	25.96	23.81
N K	9.16	9.57
Cu L	11.77	2.67
I L	1.12	0.35
Totals	100.00	

Fig. S44. EDX spectrum and data of complex **2**.

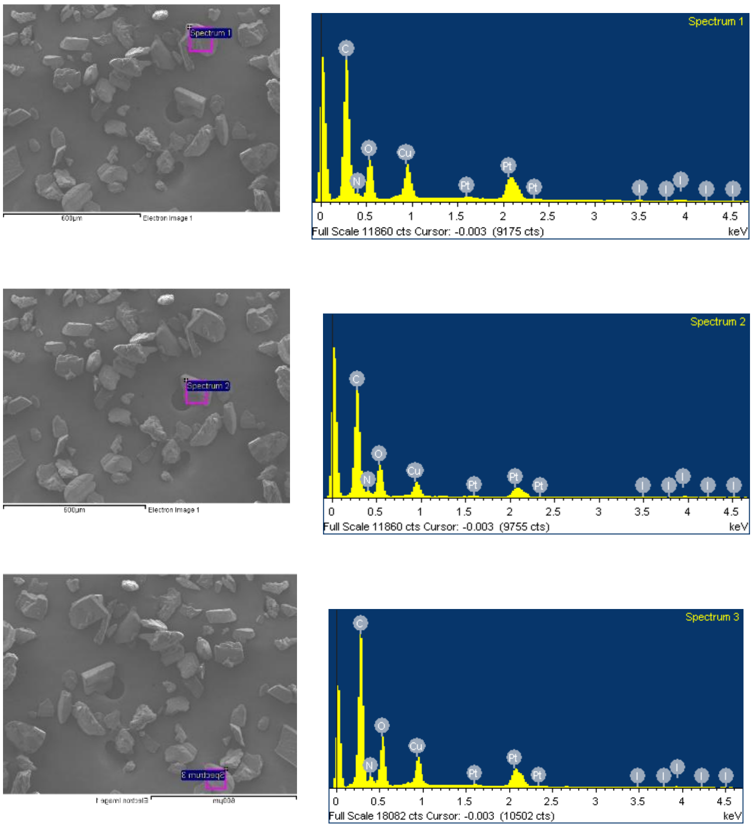

EDX data at three different positions for complex **2**.

	1st		2nd		3rd	
Element	Weight%	Atomic%	Weight%	Atomic%	Weight%	Atomic%
C K	55.27	69.73	57.34	70.61	53.18	65.46
O K	19.68	18.64	23.21	21.46	22.69	20.97
N K	7.13	7.71	4.74	5.00	9.99	10.55
Cu L	14.94	3.56	10.40	2.42	11.87	2.76
I L	2.98	0.36	4.31	0.51	2.27	0.26
Totals	100.00		100.00		100.00	

Average data

Element	Weight%	Atomic%
C K	55.26	68.60
O K	21.86	20.36
N K	7.29	7.75
Cu L	12.40	2.91
I L	3.19	0.38
Totals	100.00	

Fig. S45. EDX spectrum and data of complex **3**.

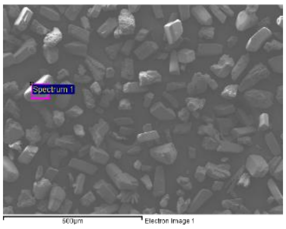

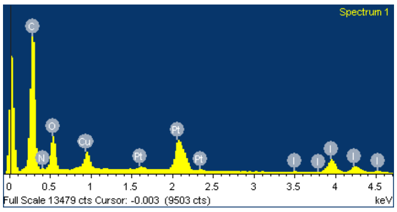

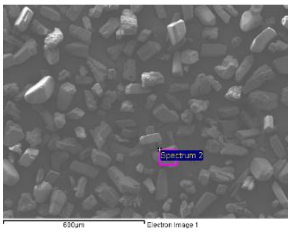

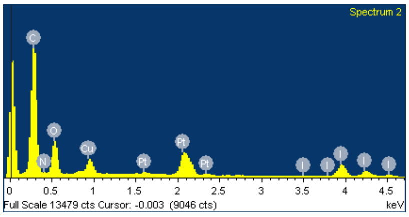

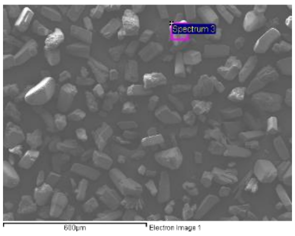

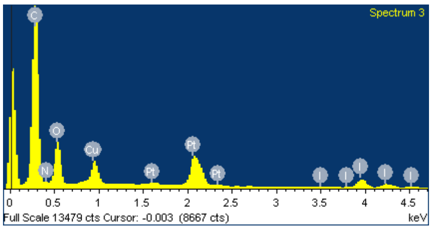

EDX data at three different positions for complex **3**.

	1st		2nd		3rd	
Element	Weight%	Atomic%	Weight%	Atomic%	Weight%	Atomic%
C K	49.91	76.29	49.98	76.91	58.72	79.11
O K	13.37	15.36	14.55	16.80	16.35	16.54
N K	1.64	2.15	0.00	0.00	0.00	0.00
Cu L	7.83	2.26	7.69	2.24	9.18	2.34
I L	27.25	3.94	27.78	4.05	15.75	2.01
Totals	100.00		100.00		100.00	

Average data

Element	Weight%	Atomic%
C K	52.87	77.44
O K	14.76	16.23
N K	0.55	0.72
Cu L	8.23	2.28
I L	23.59	3.33
Totals	100.00	

Fig. S46. EDX spectrum and data of complex **4**.

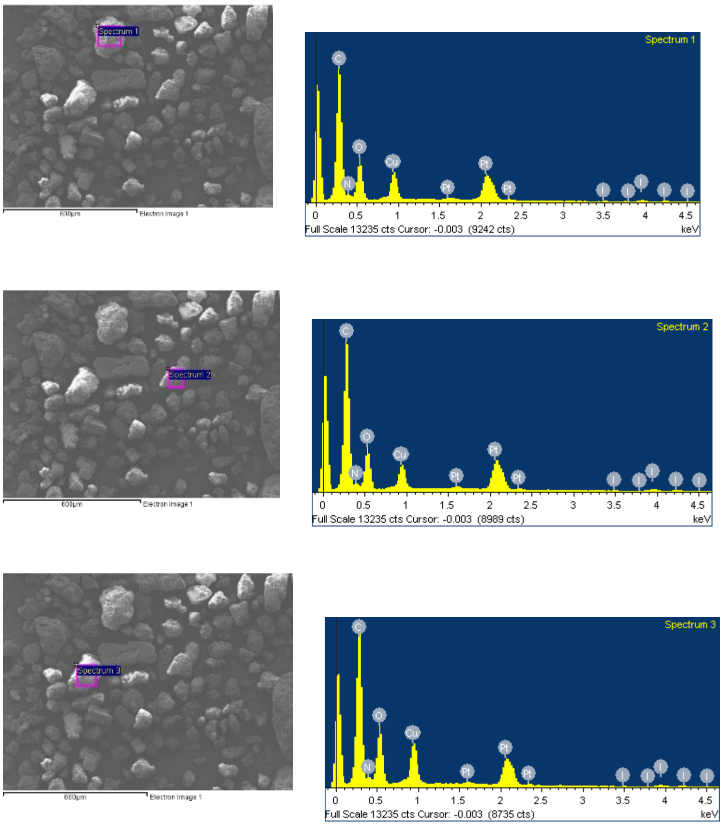

EDX data at three different positions for complex **4**.

	1st		2nd		3rd	
Element	Weight%	Atomic%	Weight%	Atomic%	Weight%	Atomic%
C K	54.74	68.61	58.82	72.46	52.58	66.57
O K	21.44	20.18	20.98	19.40	23.05	21.92
N K	7.19	7.73	4.82	5.08	7.07	7.66
Cu L	12.73	3.01	10.88	2.53	14.80	3.55
I L	3.90	0.47	4.50	0.53	2.50	0.30
Totals	100.00		100.00		100.00	

Average data

Element	Weight%	Atomic%
C K	55.38	69.21
O K	21.82	20.50
N K	6.36	6.82
Cu L	12.80	3.03
I L	3.64	0.434
Totals	100.00	

Fig. S47. EDX spectrum and data of complex **5**.

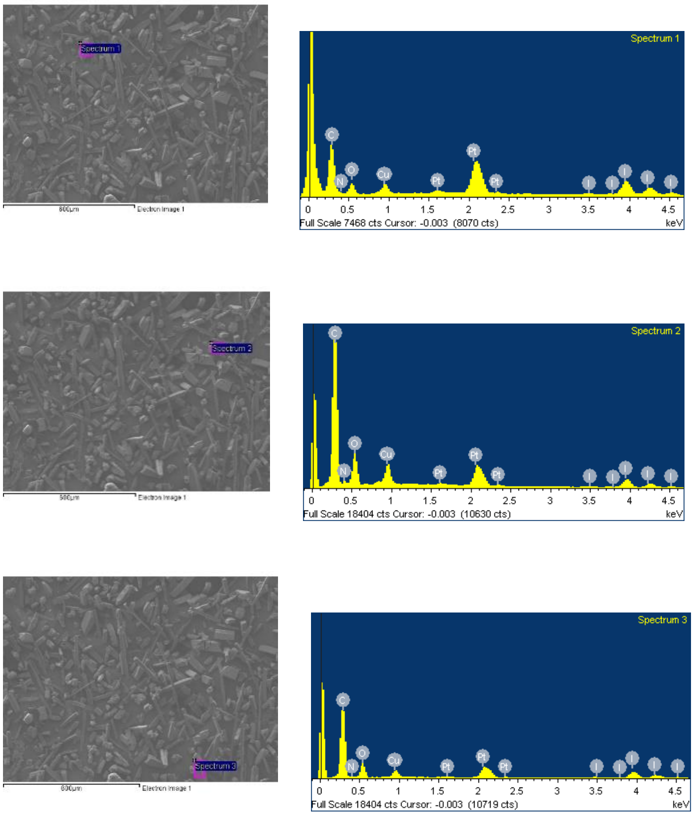

EDX data at three different positions for complex **5**.

	1st		2nd		3rd	
Element	Weight%	Atomic%	Weight%	Atomic%	Weight%	Atomic%
C K	35.28	67.64	54.46	77.07	51.25	79.71
O K	7.36	11.26	13.67	14.5	11.91	13.89
N K	6.85	10.59	2.69	3.25	0.00	0.00
Cu L	7.44	2.69	9.28	2.49	6.65	1.95
I L	43.07	7.82	19.90	2.67	30.19	4.45
Totals	100.00		100.00		100.00	

Average data

Element	Weight%	Atomic%
C K	47.00	74.81
O K	10.98	13.22
N K	3.18	4.61
Cu L	7.79	2.38
I L	31.05	4.98
Totals	100.00	

Fig. S48. EDX spectrum and data of complex **6**.

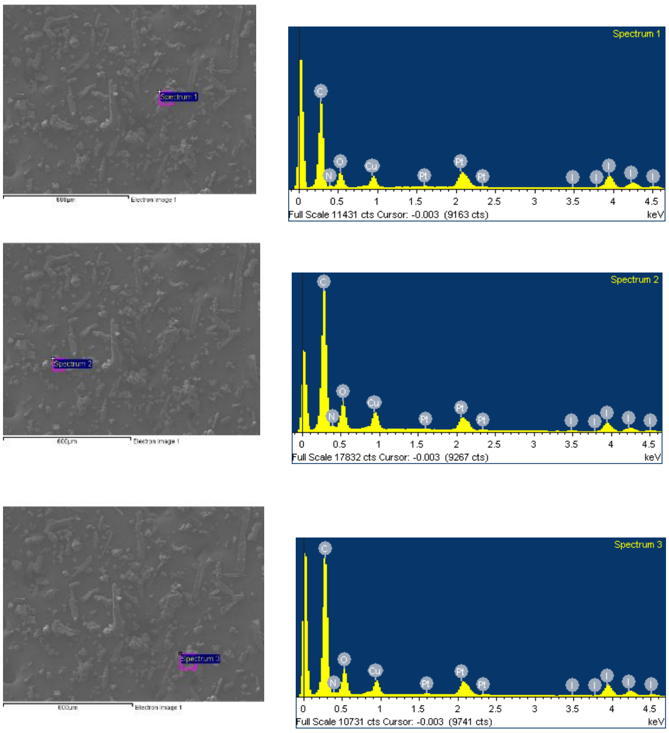

EDX data at three different positions for complex **6**.

	1st		2nd		3rd	
Element	Weight%	Atomic%	Weight%	Atomic%	Weight%	Atomic%
C K	45.95	79.62	55.97	80.19	52.40	80.24
O K	9.01	11.72	13.44	14.46	11.74	13.49
Cu L	7.80	2.55	8.92	2.41	7.44	2.15
I L	37.24	6.11	21.67	2.94	28.42	4.12
Totals	100.00		100.00		100.00	

Average data

Element	Weight%	Atomic%
C K	51.44	80.02
O K	11.40	13.223
Cu L	8.05	2.37
I L	29.11	4.39
Totals	100.00	

Fig. S49. PXRD patterns of complex **1** heated at 120 °C for 24 h.

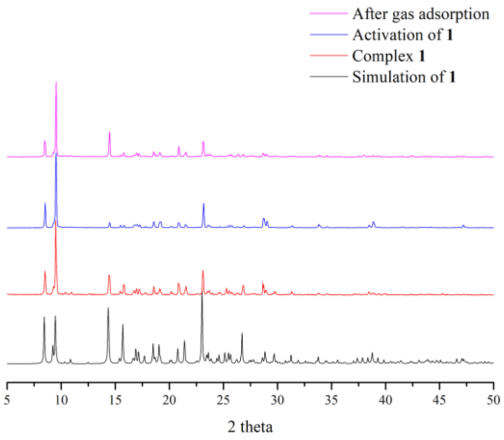

Fig. S50. PXRD patterns of complex **2** heated at 120 °C for 24 h.

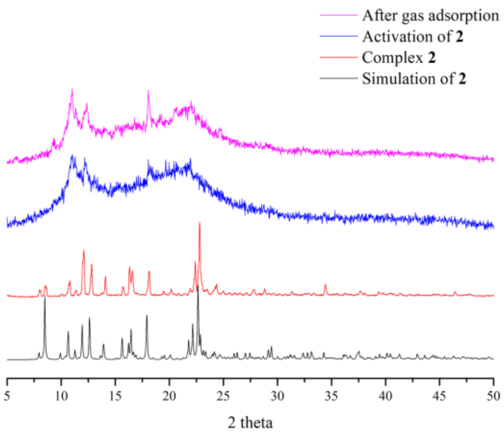

Fig. S51. PXRD patterns of complex **3** heated at 120 °C for 24 h.

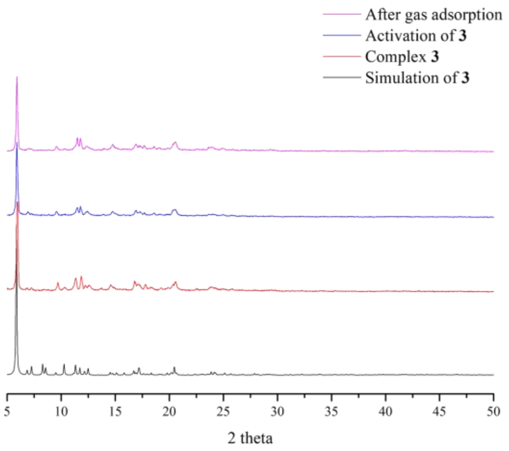

Fig. S52. PXRD patterns of complex **4** heated at 120 °C for 24 h.

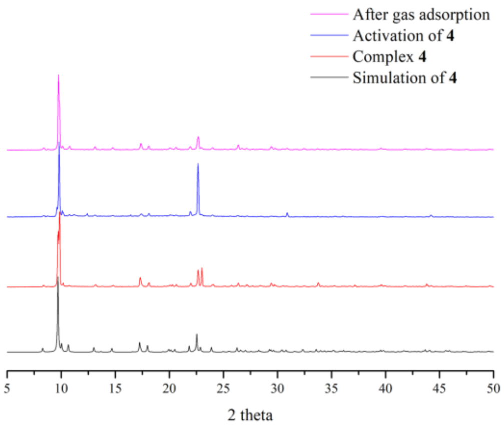

Fig. S53. PXRD patterns of complex **5** heated at 120 °C for 24 h.

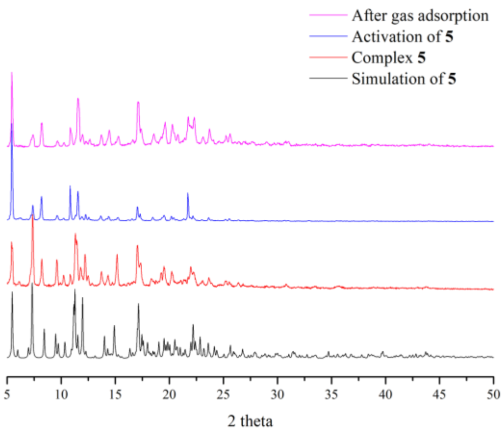

Fig. S54. PXRD patterns of complex **6** heated at 120 °C for 24 h.

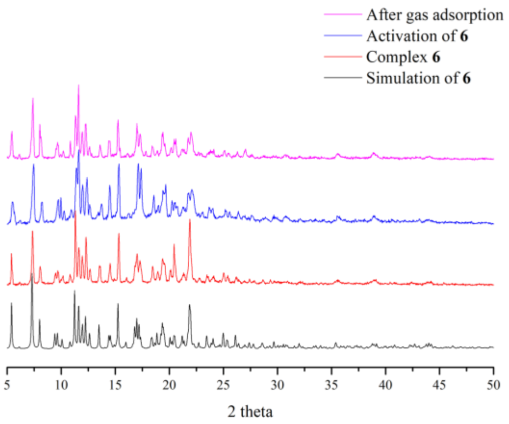

Fig. S55. N_2 adsorption−desorption isotherms for complex **1** at 77 K.

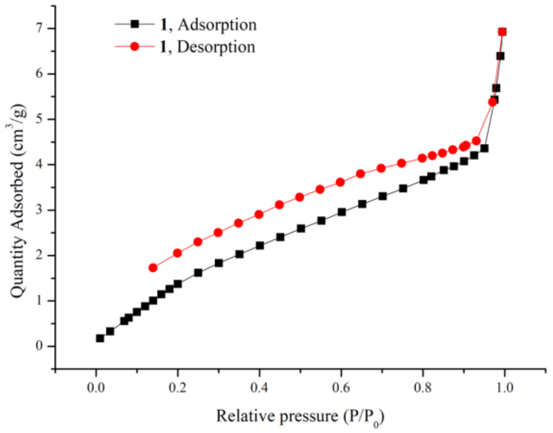

Fig. S56. N_2 adsorption−desorption isotherms for complex **2** at 77 K.

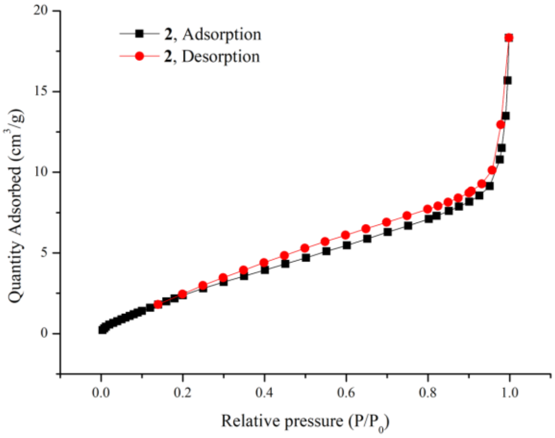

Fig. S57. N_2 adsorption−desorption isotherms for complex **3** at 77 K.

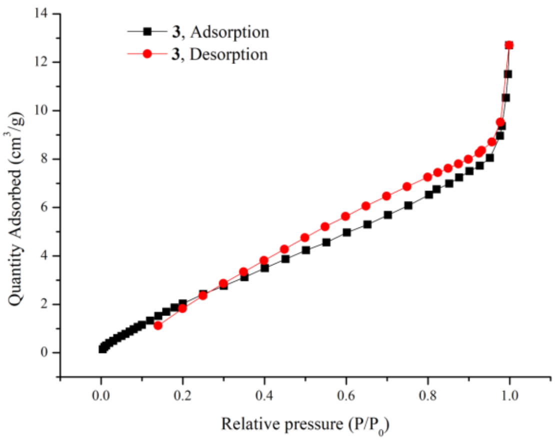

Fig. S58. N_2 adsorption−desorption isotherms for complex **4** at 77 K.

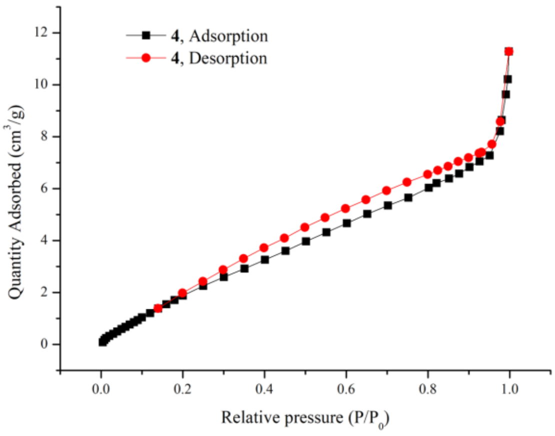

Fig. S59. N_2 adsorption−desorption isotherms for complex **5** at 77 K.

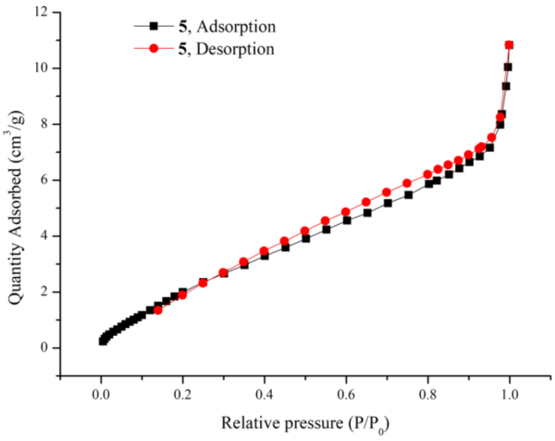

Fig. S60. N_2 adsorption−desorption isotherms for complex **6** at 77 K.

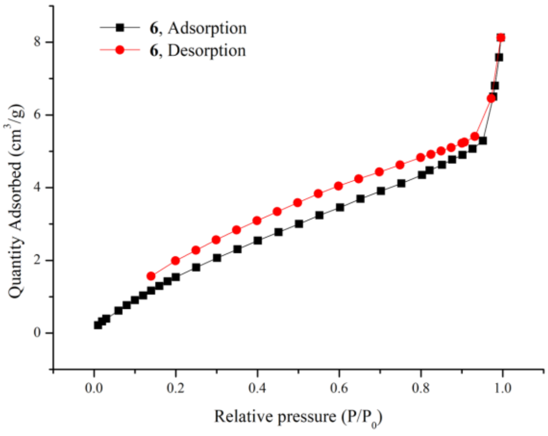

Fig. S61. Pore-size distribution curve for complex **1**.

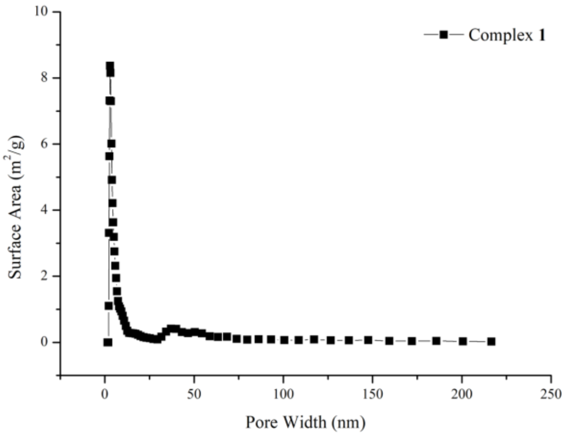

Fig. S62. Pore-size distribution curve for complex **2**.

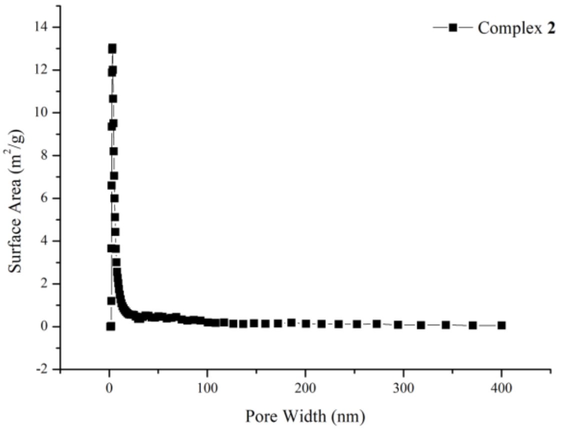

Fig. S63. Pore-size distribution curve for complex **3**.

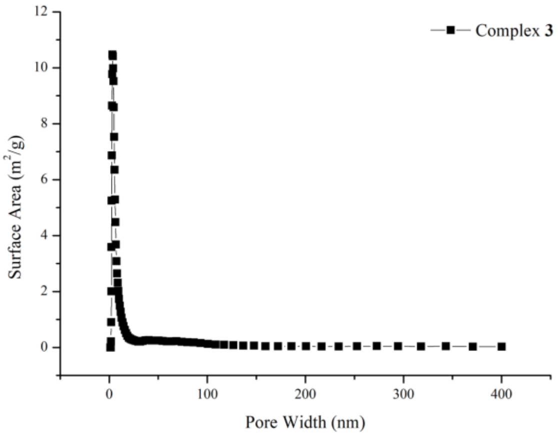

Fig. S64. Pore-size distribution curve for complex **4**.

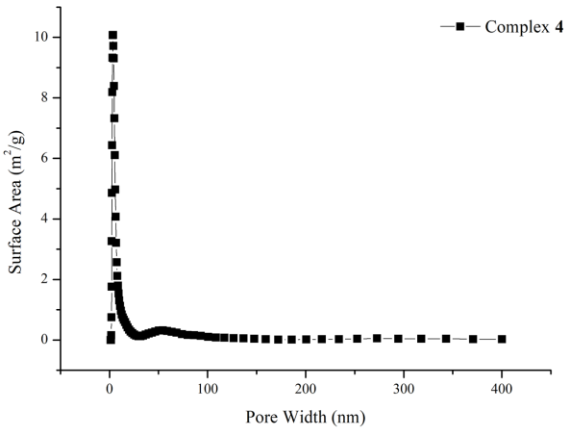

Fig. S65. Pore-size distribution curve for complex **5**.

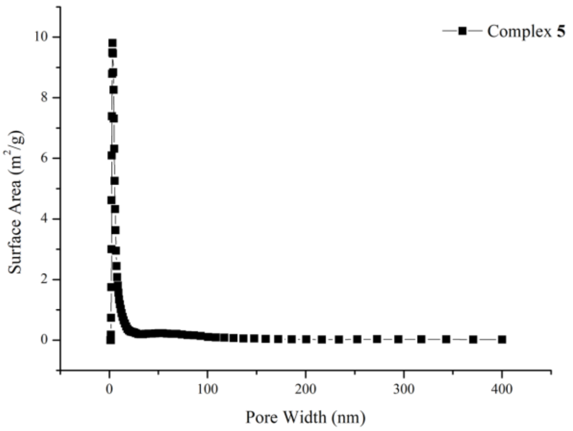

Fig. S66. Pore-size distribution curve for complex **6**.

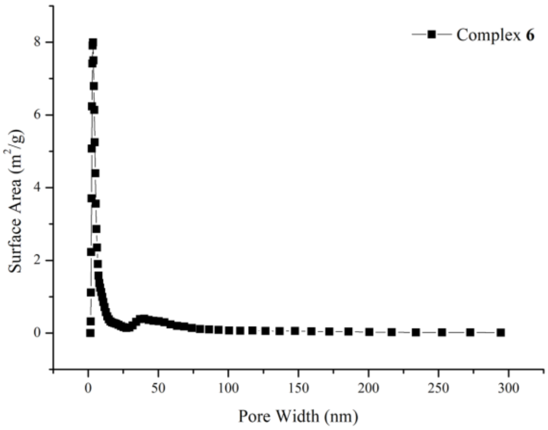

Supplement: Supplementary file 1 [file molecules-29-00311-s001.zip › supplementary_materials.pdf]
